# The Effects of Somatic Hypermutation on Neutralization and Binding in the PGT121 Family of Broadly Neutralizing HIV Antibodies

**DOI:** 10.1371/journal.ppat.1003754

**Published:** 2013-11-21

**Authors:** Devin Sok, Uri Laserson, Jonathan Laserson, Yi Liu, Francois Vigneault, Jean-Philippe Julien, Bryan Briney, Alejandra Ramos, Karen F. Saye, Khoa Le, Alison Mahan, Shenshen Wang, Mehran Kardar, Gur Yaari, Laura M. Walker, Birgitte B. Simen, Elizabeth P. St. John, Po-Ying Chan-Hui, Kristine Swiderek, Stephen H. Kleinstein, Galit Alter, Michael S. Seaman, Arup K. Chakraborty, Daphne Koller, Ian A. Wilson, George M. Church, Dennis R. Burton, Pascal Poignard

**Affiliations:** 1 Department of Immunology and Microbial Science, The Scripps Research Institute, La Jolla, California, United States of America; 2 IAVI Neutralizing Antibody Center, The Scripps Research Institute, La Jolla, California, United States of America; 3 Scripps Center for HIV/AIDS Vaccine Immunology and Immunogen Discovery, The Scripps Research Institute, La Jolla, California, United States of America; 4 Department of Mathematics, Massachusetts Institute of Technology, Cambridge, Massachusetts, United States of America; 5 Harvard-MIT Division of Health Sciences and Technology, Massachusetts Institute of Technology, Cambridge, Massachusetts, United States of America; 6 Department of Genetics, Harvard Medical School, Boston, Massachusetts, United States of America; 7 Department of Computer Science, Stanford University, Stanford, California, United States of America; 8 Biomedical Informatics Training Program, Stanford University School of Medicine, Stanford, California, United States of America; 9 AbVitro Inc., Boston, Massachusetts, United States of America; 10 Department of Integrative Structural and Computational Biology, The Scripps Research Institute, La Jolla, California, United States of America; 11 International AIDS Vaccine Initiative, New York, New York, United States of America; 12 Ragon Institute of Massachusetts General Hospital, Massachusetts Institute of Technology, and Harvard University, Boston, Massachusetts, United States of America; 13 Department of Chemical Engineering, Massachusetts Institute of Technology, Cambridge, Massachusetts, United States of America; 14 Department of Physics, Massachusetts Institute of Technology, Cambridge, Massachusetts, United States of America; 15 Department of Pathology, Yale University School of Medicine, New Haven, Connecticut, United States of America; 16 454 Life Sciences – A Roche Company, Branford, Connecticut, United States of America; 17 Theraclone Sciences, Inc., Seattle, Washington, United States of America; 18 Beth Israel Deaconess Medical Center, Boston, Massachusetts, United States of America; 19 Department of Chemistry, Institute for Medical Engineering & Science, Massachusetts Institute of Technology, Cambridge, Massachusetts, United States of America; University of Zurich, Switzerland

## Abstract

Broadly neutralizing HIV antibodies (bnAbs) are typically highly somatically mutated, raising doubts as to whether they can be elicited by vaccination. We used 454 sequencing and designed a novel phylogenetic method to model lineage evolution of the bnAbs PGT121–134 and found a positive correlation between the level of somatic hypermutation (SHM) and the development of neutralization breadth and potency. Strikingly, putative intermediates were characterized that show approximately half the mutation level of PGT121–134 but were still capable of neutralizing roughly 40–80% of PGT121–134 sensitive viruses in a 74-virus panel at median titers between 15- and 3-fold higher than PGT121–134. Such antibodies with lower levels of SHM may be more amenable to elicitation through vaccination while still providing noteworthy coverage. Binding characterization indicated a preference of inferred intermediates for native Env binding over monomeric gp120, suggesting that the PGT121–134 lineage may have been selected for binding to native Env at some point during maturation. Analysis of glycan-dependent neutralization for inferred intermediates identified additional adjacent glycans that comprise the epitope and suggests changes in glycan dependency or recognition over the course of affinity maturation for this lineage. Finally, patterns of neutralization of inferred bnAb intermediates suggest hypotheses as to how SHM may lead to potent and broad HIV neutralization and provide important clues for immunogen design.

## Author Summary

A majority of the over 30 million HIV-1 infected individuals worldwide live in poorly resourced areas where multiple boost strategies, which are likely needed to generate highly mutated antibodies, present formidable logistical challenges. Accordingly, developing new vaccination strategies that are capable of generating highly mutated antibodies should be an active area of research. Another approach, that is not mutually exclusive, is to identify new bnAbs that are both broad and potent in neutralization, but are much less mutated than the bnAbs that currently exist. Here, we have identified bnAbs that are approximately half the mutation frequency of known bnAbs, but maintain high potency and moderate breadth. These less mutated bnAbs offer an important advantage in that they would likely be easier to induce through vaccination than more mutated antibodies. By characterizing these putative intermediates, we can also better estimate how affinity maturation proceeded to result in an antibody with broad and potent neutralization activity and offer more focused strategies for designing immunogens capable of eliciting these less mutated bnAbs.

## Introduction

A successful vaccine against HIV will likely require the elicitation of antibody responses capable of neutralizing a majority of global isolates. Recent work has suggested that 5–20% of HIV chronically-infected individuals naturally develop broadly neutralizing responses to some degree, but how these responses emerge and mature are unclear [1]–[8]. A common observation among bnAbs is their unusually high level of somatic hypermutation (SHM), which on average constitutes around 20% divergence (range: 7–32%) from the putative germline nucleotide sequence (nt) for the variable heavy chain (V_H_J_H_) region (D_H_-genes were left out of these analyses because of ambiguity associated with D-gene assignment) [2]–[7], [9]. For example, the CD4 binding site bnAb VRC01 is 30% and 19% mutated in its variable heavy (V_H_J_H_) and light (V_L_J_L_) chain sequence, respectively [4], [6]. The V2 quaternary epitope-specific bnAbs PG9 and PGT145 are relatively less mutated with 14–19% mutation frequency in V_H_J_H_ and 11–17% in V_L_J_L_, but both have unusually long CDRH3s of 30–33 amino acids [2], [5]. Finally, the recently described PGT121, 128 and 135 antibodies, which bind to protein-glycan epitopes in the variable V3 and V4 regions and demonstrate the highest potency yet observed against a broad panel of HIV isolates, are 17–23% divergent in V_H_J_H_ and 11–28% divergent in V_L_J_L_ [5], [8], [10]. Of note, a number of the bnAbs also have insertions or deletions (indels) in their variable regions and recent crystal structures have identified indels as critical for protein or glycan contacts on HIV Env [4], [7], [11]. Interestingly, gp120-reactive antibodies that show no or low neutralizing activity from chronically HIV-infected individuals demonstrate a relatively high but lesser degree of SHM than bnAbs, in the range of 9–12% in V_H_J_H_ [12], [13].

In contrast to HIV bnAbs, antibodies from vaccination in general have an average nt mutation frequency of 6% (range: 1–30%) in the V_H_, which has cast doubts on the likelihood of eliciting bnAbs through vaccination [14]–[21]. We note that while these previous studies are caveated by insufficient sampling of antibody responses, due in large part to technological limitations, they nonetheless provide an approximation of the large discrepancy in mutation frequency between antibodies typically elicited through vaccination and HIV bnAbs. Currently, no immunogen has reliably elicited significant levels of HIV bnAbs to circulating viruses. Part of this failure might be due to inadequate immunogen design, but assuming high mutation levels play a significant role in enabling neutralizing breadth and potency, it is also possible that current immunization approaches are failing because of an inability to elicit sufficient levels of SHM. In one report, immunization with monomeric gp120 in humans was only capable of eliciting antibodies with an average mutation frequency of ∼6% [18]. Similarly, Env trimer immunization in rhesus macaques produced an average V_H_ mutation frequency of 10% [19]. A further complexity is the association of broad neutralization with indels, sometimes relatively long, in some bnAbs as described above. The frequency of indels is associated with the extent of SHM; typical immunization procedures that produce moderate levels of SHM produce a relatively low number of indels and the frequency of longer indels is particularly uncommon. The frequency of indels among bnAbs may therefore present another obstacle for their elicitation by vaccination [22].

Given the large gap between the average mutation frequency of antibodies elicited by vaccination and bnAbs, it will prove helpful to identify broad and potent neutralizing antibodies with lower mutation frequencies as viable candidates for bnAb elicitation by vaccination. The levels of SHM that are necessary for broad and potent neutralization is poorly defined. Much work has been done to demonstrate that complete reversion of V_H_D_H_J_H_ and V_L_J_L_ sequences of HIV bnAbs to the corresponding germline sequences generally abrogates neutralizing and binding activity, which suggests that some degree of SHM plays an important role in the development of neutralizing breadth and potency [23]–[26]. More recent studies involving reversion of all framework (FR) mutations was shown to affect binding and neutralization for bnAbs but not for non-broadly neutralizing antibodies, suggesting that high levels of SHM are necessary for neutralization breadth and potency [9]. Similarly, a 12 amino acid reversion in the V_H_ of VRC01 was sufficient to negatively affect neutralization breadth [4]. In a different study, characterization of inferred intermediates from longitudinal samples identified less-mutated CD4bs bnAb intermediates, though these putative precursors were greatly limited in neutralization breadth and potency [27]. Based on longitudinal serum neutralization data, it appears that neutralization breadth generally requires up to 4 years post-infection in order to develop, further supporting the idea that antibodies are going though many rounds of maturation before gaining breadth of neutralization [8], [10]. Longitudinal analysis of a SHIV-infected macaque, however, demonstrated the rapid emergence of neutralization breadth and potency against the N332 epitope suggesting potential differences in the dependence on SHM levels [8], [28], [29].

Previous work has attempted to map the maturation process from germline to affinity matured antibody for a group of highly broad and potent CD4 binding-site antibodies using deep sequencing analysis [6]. However, less mutated antibody intermediates identified by traditional phylogeny in that study did not demonstrate neutralization activity. To identify less-mutated intermediates with broad and potent neutralization, we used deep sequencing to study the development of neutralization breadth in the donor from whom bnAbs PGT121–123 were isolated. These antibodies are among the most potent bnAbs described to date and provide protection in macaques at relatively low serum levels [30]. Thus, the PGT121–123 family of antibodies are promising vaccine leads because their high potency suggests that even modest titers of antibody in the serum might be capable of contributing to protection. Ideally, the evolution of a bnAb response would be followed in longitudinal samples as infection progressed. However, since such samples were not available for this donor, we used 454 pyrosequencing to probe the memory B cell antibody repertoire and model the lineage evolution of the PGT121–123 family. We performed this analysis on PBMCs drawn at the same time point as that previously used to isolate PGT121–123 [5]. To analyze the resulting set of sequences, we developed a novel method called ImmuniTree, which is an alternative approach to conventional phylogenetic analyses and is designed specifically to model antibody SHM. Our results suggest that the PGT121 family of antibodies is capable of demonstrating appreciable breadth and high potency at relatively modest levels of SHM.

## Results

### ImmuniTree models SHM of the PGT121–123 lineage

**Figure 1:**
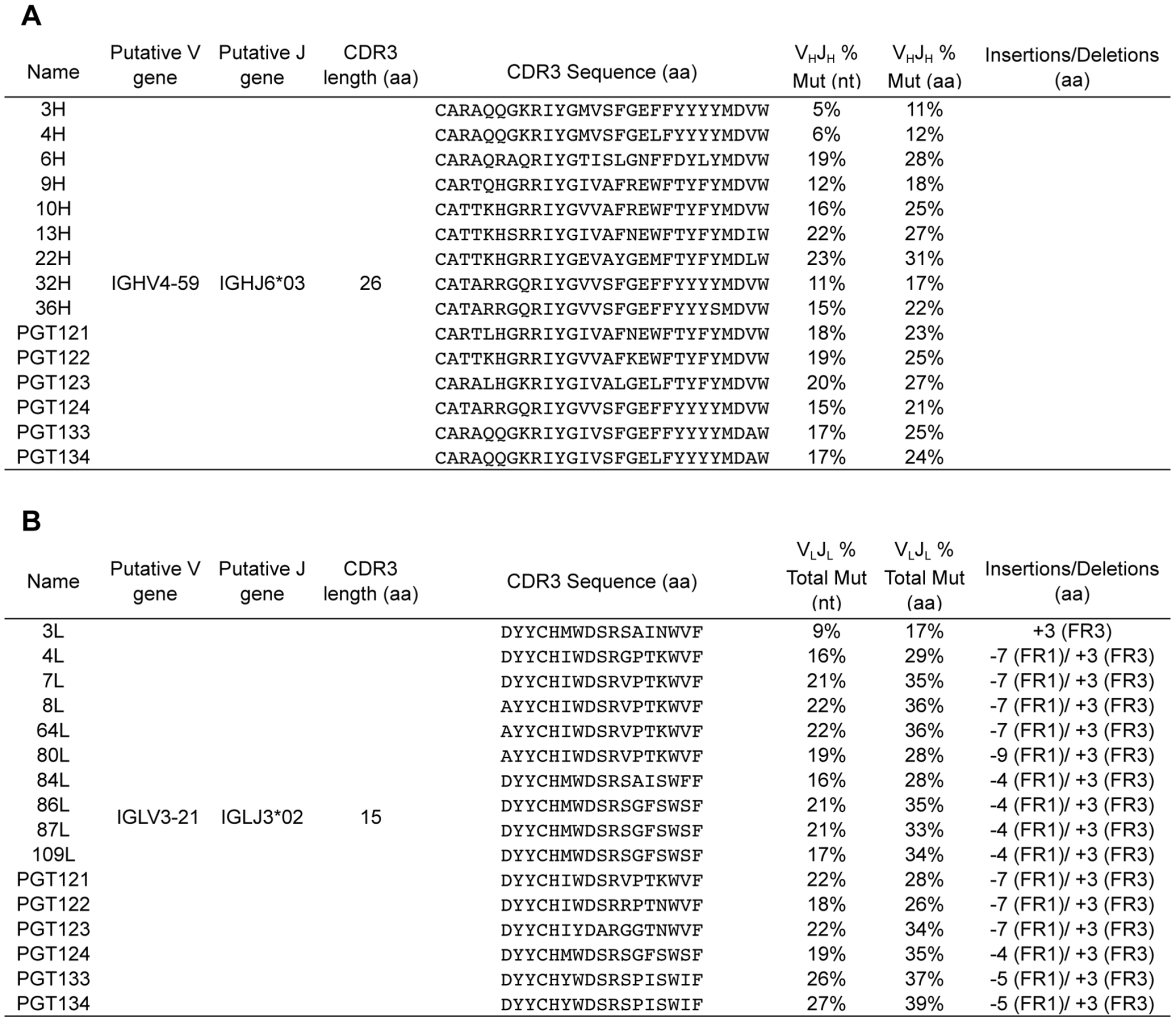
Mutation summary of selected heavy and light chain clones. Mutation frequency was calculated over the V-gene and J-gene as nucleotides or amino acids differing from the putative germline sequence for (**A**) heavy chain sequences and (**B**) light chain sequences. The CDR3 regions and insertions and deletions were excluded from the analysis. CDR3 lengths were determined according to the IMGT definition. Analyses were performed with the SciPy stack [53] and figures were generated using matplotlib [54] and graphviz [55].

To produce libraries for 454 sequencing, gene-specific primers were used on 54,000 sorted IgG+ memory B cells to amplify >>IGHV4-59 and IGLV3-21 gene families from which the PGT121–124 and PGT133–134 antibodies were derived [5]. PGT124, PGT133, and PGT134 are somatic variants of PGT121–123 monoclonal antibodies (mAbs) that were recently isolated through direct functional screening and by antigen-based cell sorting (Figure 1). These libraries were then submitted to 454 sequencing to yield a total of 376,114 heavy-chain and 530,197 light-chain reads.

**Figure 2:**
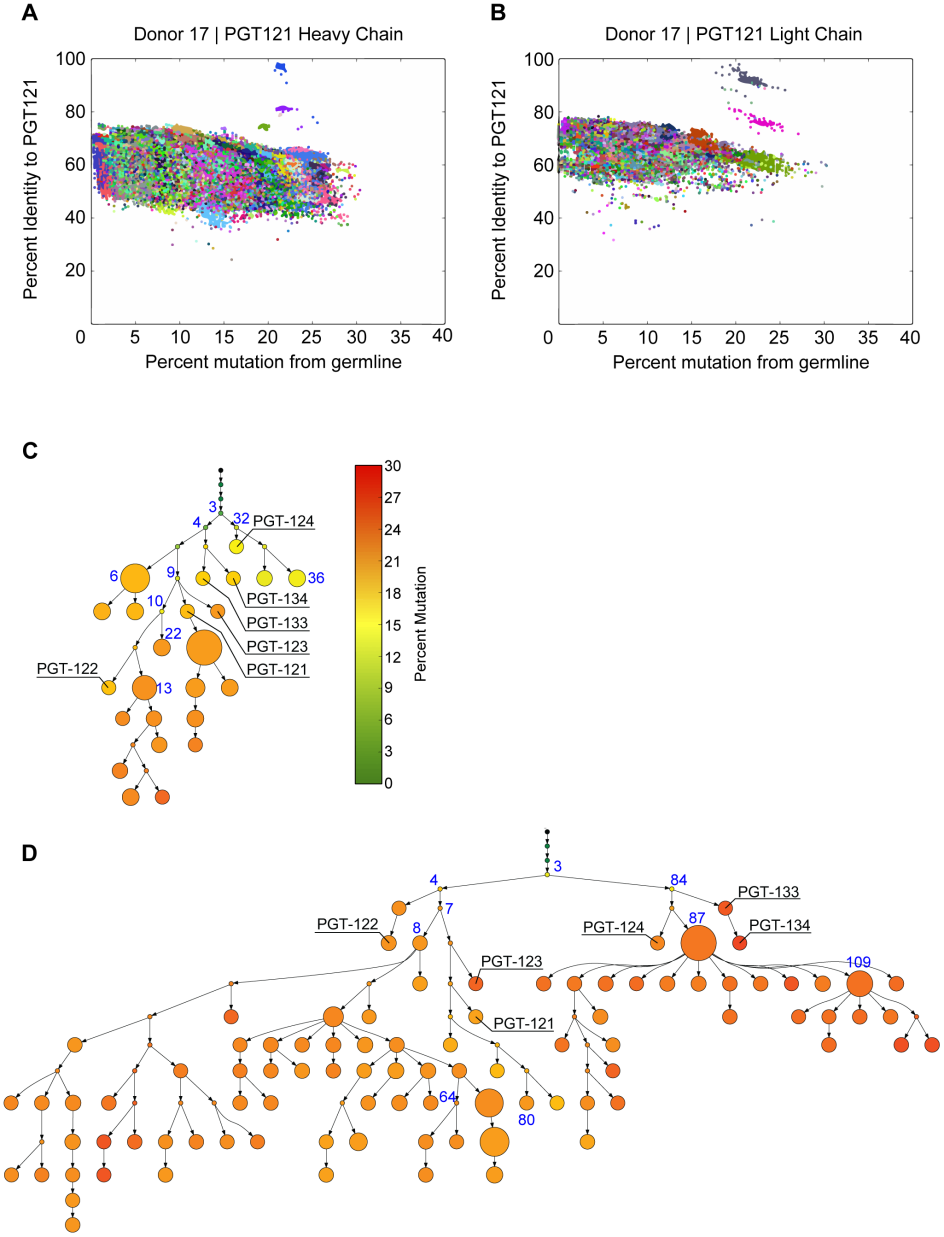
PGT121–123 variants identified by deep sequencing were used to build phylogenetic trees using ImmuniTree. (**A**) An example identity (y-axis) and mutation (x-axis) plot for PGT121 used to identify PGT121-variants for heavy chain and for (**B**) light chain; each color represents a unique clone. (**C**) Heavy chain and (**D**) light chain SHM phylogenies inferred by the ImmuniTree algorithm. Nodes that were inferred by the algorithm are represented as small circles. Nodes representing observed sequences are depicted as larger circles; node size is proportional to the number of reads assigned to that node. The trees are colored based on level of mutation from germline. Previously isolated affinity matured mAbs are labeled (e.g. PGT122) and nodes selected for synthesis and characterization are labeled blue.

To define unique clones, the V and J gene for each read was determined along with its percent mutation from the corresponding germline sequence. We then analyzed the clustering quality using multiple values for the cutoff range. The distribution of cophenetic distances in the hierarchical clustering linkage tree showed that a cutoff of 4–5 “edits” was optimal, because at that distance, the distribution had a qualitative change most likely associated with sequencing error. Given the average length of the International Immunogenetics (IMGT)-defined complementary determining region 3 (CDR3), 4–5 edits corresponds to a 90% identity using USEARCH's iddef 1 option [31]. Target PGT antibody sequences were manually selected for further analysis as follows. Each read was scored on its identity to a PGT antibody and on its mutation level from the IMGT reference germline sequence. The vast majority of reads formed several large, low-identity clusters in a divergence-mutation scatter plot; in some cases, there existed small clusters of high-identity reads separate from the large clusters (Figure 2A–B and Figure S1, S2). These small clusters were manually extracted and carried forward for phylogeny. This approach identified a total of 97 heavy-chain and 530 light-chain sequences as PGT121–134 variants. The greater number of light chain variants is likely due partly to more raw reads from the sequencing run and partly to library preparation, which has also been observed by other deep sequencing studies of HIV antibody repertoires [6], [32]. Indeed, the primers we used to amplify the heavy chains were not as specific as the light chain primers and so there may have been less enrichment for heavy chain sequences. Sequence variants were then used to model the history of the PGT121–134 antibody lineage so as to experimentally characterize early less-mutated variants. We designed a new Bayesian phylogeny algorithm, ImmuniTree, to model the SHM of this lineage (Text S1).

Existing approaches to phylogeny were suboptimal for our experiment because of two principal issues. Firstly, existing algorithms treat 454 read errors as true somatic mutations, thereby leading to excessive branching in the phylogeny and difficulties in interpretation. ImmuniTree addresses this obstacle by taking advantage of the distinctly different nucleotide transition characteristics between clonal SHM and 454 sequencing errors. Secondly, traditional phylogeny algorithms do not explicitly model whether or not to generate an internal node. Instead, these algorithms either always infer an internal unobserved intermediate (e.g. neighbor joining), or never do so (e.g. minimal spanning trees). This poor representation inaccurately models reality, where noisy yet highly clonal data are observed from only a subset of leaves and internal clones.

Though emerging methods address only one or neither of these issues [33], ImmuniTree addresses both explicitly (Text S1). For each antibody clone, the algorithm models SHM as a birth-death model, where reads are generated from clonal populations using a known sequencing error model. The algorithm jointly models sequencing error alongside somatic mutation and collapses reads that differ due to sequencing error. The approach reduces inference variance and facilitates human interpretation when choosing sequences for the subsequent evaluation of binding and neutralization.

The ImmuniTree algorithm relies on a probabilistic model that describes both the expansion process of a clone and the sequencing process. It iteratively explores this probabilistic space using the Markov chain Monte Carlo framework [34], producing a new hypothetical tree in each iteration, aimed at describing how the data were generated. In the hypothesized tree, each node is associated with one sequence, and the reads are associated with the nodes of the tree. The disagreements between the sequence of a node and its parent represent new hypermutations. The disagreements between a read and the sequence of its node represent sequencing errors. The mechanics of the algorithm guide the search to high-likelihood areas of the probabilistic space. During each iteration the tree is constantly manipulated, intermediate nodes are stochastically added and deleted, and if such nodes contribute to a tree that better explains the data, they are likely to stay. Then, the DNA sequences of all the nodes in the hypothesized tree, including intermediates, are jointly updated by an exact inference algorithm [35]. At the end of the search, the algorithm returns the tree with the highest likelihood.

Given the tree topology, and the association of reads to nodes in the tree (the “evidence”), we use belief propagation algorithm to infer the sequences of all the nodes, including intermediate nodes. The belief-propagation algorithm is a message-passing algorithms, a generalization of the backward-forward algorithm for hidden Markov models (HMMs), which computes the set of sequences that yield the highest likelihood according to the mutation model.

Each node in the tree receives “messages” from its children stating their best estimate for its sequence. Each child's estimate is based on the evidence in its subtree, taking into account the mutation model. Those messages are integrated with the evidence at the node itself into a new message that is sent to its own parent, estimating the parent's sequence. Once the root of the tree received all the messages from its children, it sends to each of its children its best estimate, now fully informed, for their sequence. This process is repeated until all the nodes in the tree are instantiated with their optimal sequences, given to them by their parent.

When applied to the PGT121–134 variants, ImmuniTree generated trees for heavy and light chain sequences with a total of 37 nodes (named 1H through 37H) and 120 nodes (named 1L through 120L), respectively (Figure 2C–D). Within these two trees, 13 internal nodes in the heavy chain tree and 28 internal nodes of the light chain tree were inferred statistically, and did not have direct sequencing data (Figure 2C–D). As expected, trees built without correcting for sequencing error resulted in a larger number of nodes and branches, which confounds lineage analysis (Figure S3). Overall, compared to the inferred internal nodes, the observed nodes in both heavy and light chain trees were relatively highly mutated with an average divergence from germline of 18% for V_H_ and 24% for V_L_. The observation that only highly mutated sequences were found suggests that early precursors might be absent in the B cell memory compartment or are present at very low levels, possibly because the donor blood samples were obtained several years into the infection. Indeed, this observation is consistent with other antibody lineage studies for which inferred intermediates were calculated because less-mutated sequences were absent or were present at very low frequencies in the sequencing dataset in both late time point samples and even in early blood draws from longitudinal studies [6], [27], [32]. We were particularly interested in whether these less-mutated inferred precursors could also exhibit broad neutralization.

### Neutralization activity positively correlates with degree of SHM

**Figure 3:**
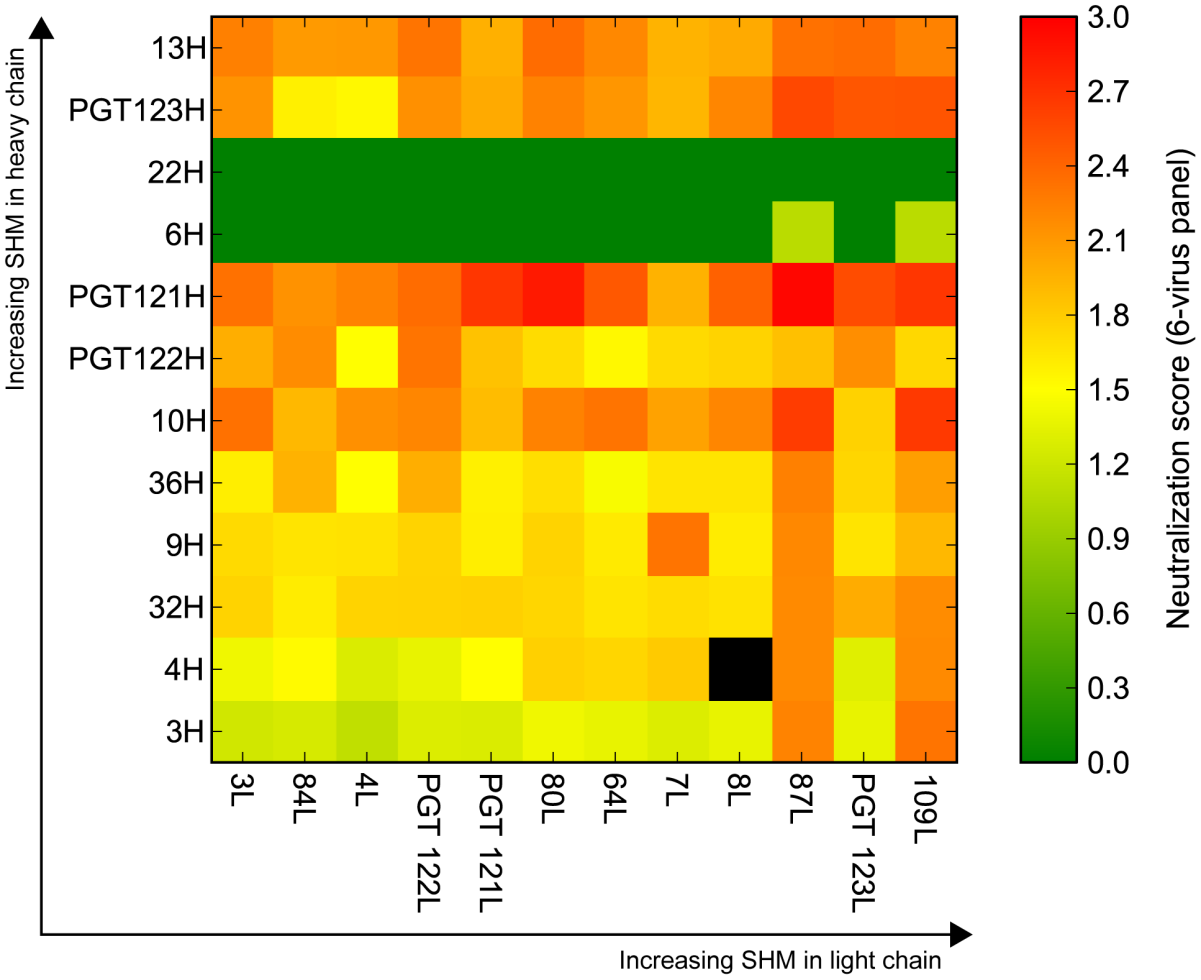
Selected heavy and light chain clones were paired and tested for neutralization breadth and potency on a cross-clade 6-virus panel. Neutralization breadth and potency summary of all the paired clones. The clones are arranged from least (bottom-left) to most (top-right) mutated with heavy chain listed on the left and light chain listed across the bottom. Boxes are colored according to neutralization score, which is defined as mean(log_10_(10/IC_50_)); black boxes represent pairs whose neutralization scores were not determined.

To see if observed and inferred sequences were capable of generating functional antibodies, we paired heavy and light chain sequences from nodes of varying mutation levels and measured neutralization activity on a cross-clade 6-virus panel to evaluate neutralization breadth and potency (Figure 3) [36]. As shown in a recent study, the PGT121 family of antibodies retained full function only when heavy and light chains within the lineage were paired and were not functional or suffered a great reduction in neutralization and binding when paired with heavy or light chains from other N332-specific antibodies [37]. Moreover, the structural similarity between somatic variants suggests that heavy and light combinations within the lineage should give rise to antibodies capable of binding and/or neutralization [38], [39]. Indeed, the variants and putative intermediates selected in this study share many of the amino acid motifs characteristic of this family of antibodies and are therefore presumed to fold into similar antibody structures. Thus, while the heavy and light chain pairs presented here may not recapitulate natural *in vivo* pairing, the differences in SHM levels between chimeras can still offer insight into the contribution of SHM to the antibody lineage's neutralization breadth and potency.

The mutation levels of the selected clones in comparison to known heavy and light chain pairs from this donor are listed in Figure 1. We selected the intermediates based only on mutation frequency. For example, we chose the least mutated nodes 3H for heavy chain and 3L for light chain to determine if neutralization activity was achievable at these levels of SHM. Additionally, we chose nodes that followed consecutively in SHM levels (e.g. 9H to 10H to 13H for heavy chain and 84L to 87L to 109L for light chain) to determine if the level of SHM correlates with neutralization breadth and/or potency. Surprisingly, pairing of the least mutated nodes (3H+3L) resulted in a variant that demonstrated notable neutralization activity (Figure 3). Also, antibody clones comprising heavy and/or light chain sequences that are more divergent from germline consistently demonstrated greater neutralization potency and/or wider breadth on the 6-virus panel (Figure 3 and Figure S4). Some combinations from observed sequences, however, were unable to demonstrate neutralizing activity, which could be due to either incorrect chain pairing or the accumulation of deleterious mutations during SHM that rendered them no longer functional.

To investigate further, we chose to characterize putative intermediates for the affinity matured PGT121 and PGT124 antibodies, which lie on different branches of the evolutionary tree and have slightly different neutralization profiles (Figure 2C–D and Figure S5, S6). As an inferred precursor to both antibodies, we chose the least mutated nodes 3H from the heavy chain tree and 3L from the light chain tree. To investigate intermediate levels of SHM, we chose 9H as a heavy chain precursor of PGT121–123 and 32H as a precursor of PGT124. Finally, we also tested pairing with node 87L from the light chain tree as it demonstrated very high neutralization breadth and potency when paired with various heavy chains.

**Figure 4:**
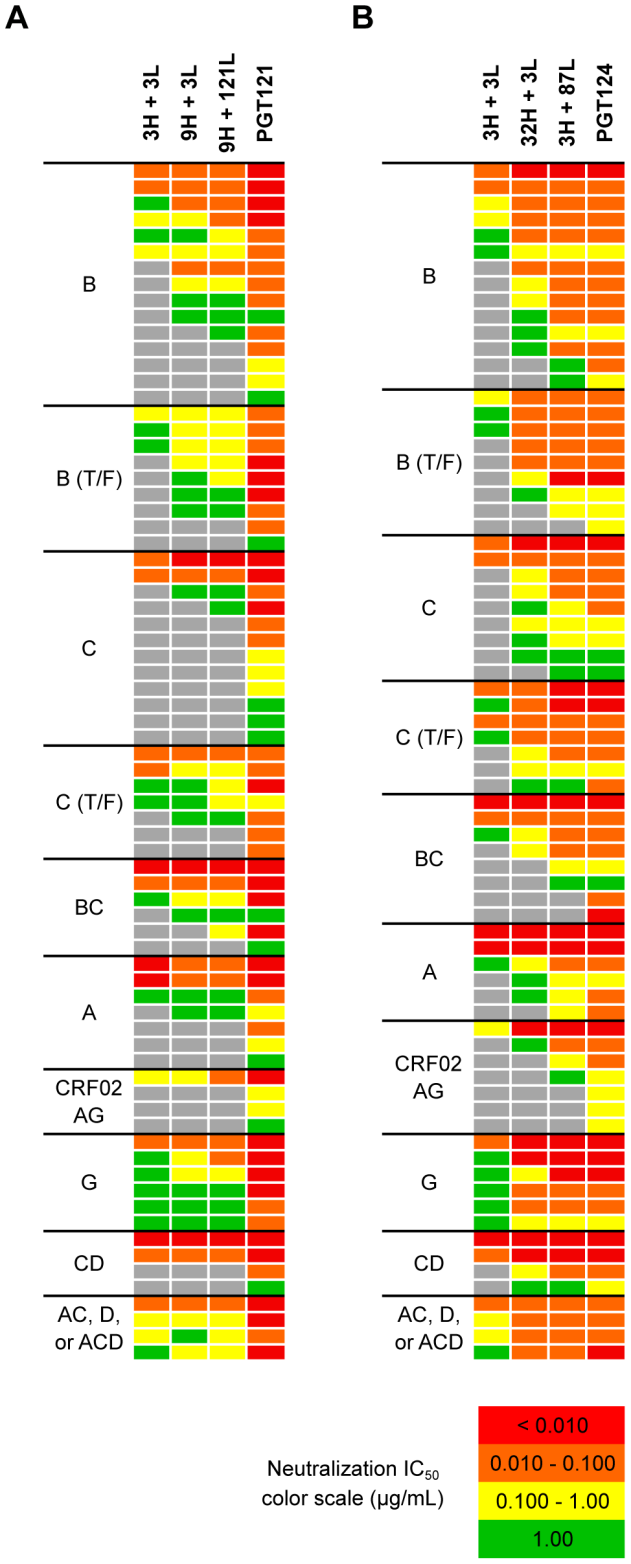
Selected heavy and light chain clones were paired and tested for neutralization breadth and potency on a cross-clade 6-virus panel. (**A**) Heavy and light chain nodes leading to mAb PGT121 and (**B**) PGT124 were paired and tested on a 74-virus panel of PGT121- or PGT124-sensitive viruses. Boxes are colored by IC_50_ values (µg/ml) of each isolate neutralized.

**Figure 5:**
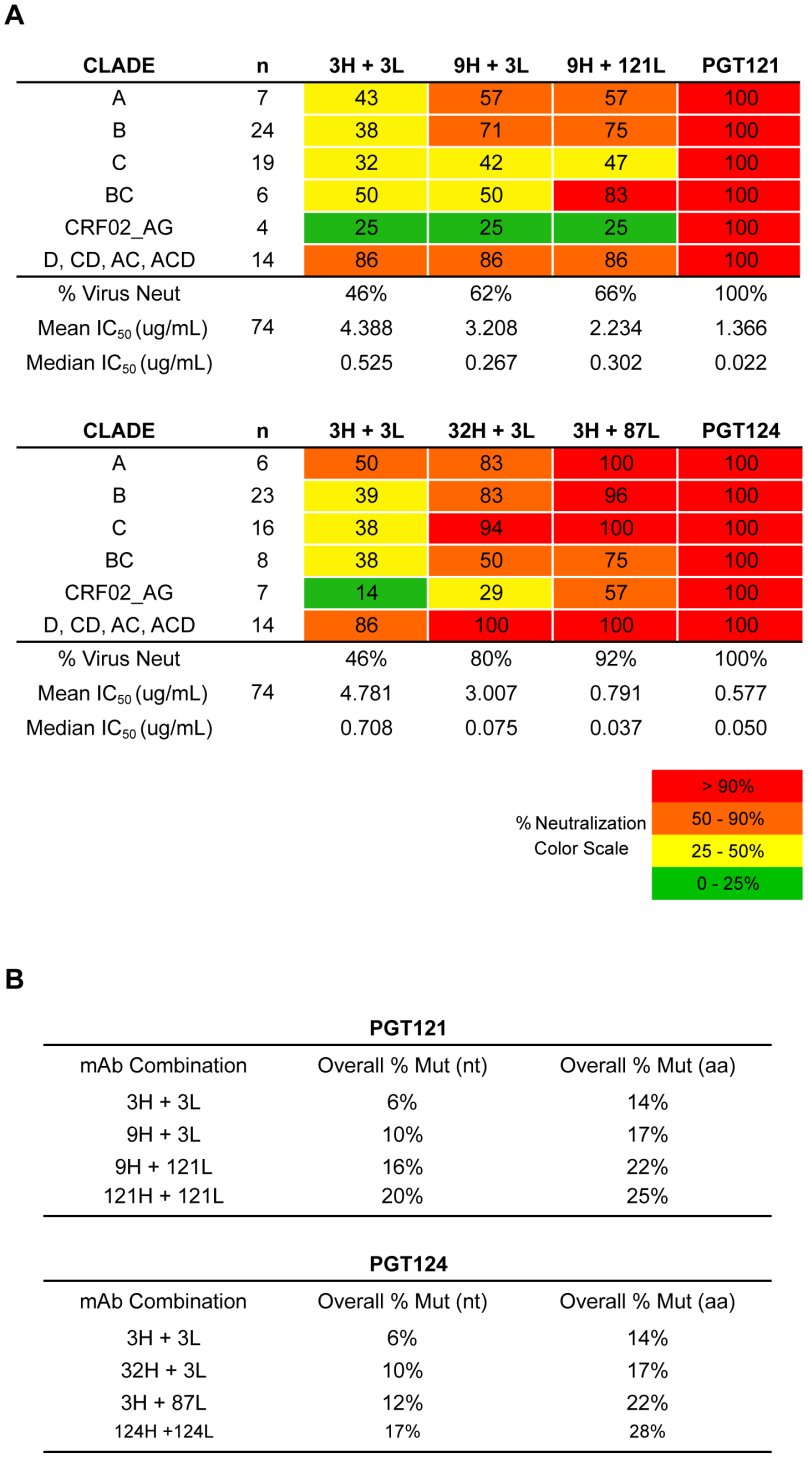
Higher levels of somatic hypermutation correlates with greater neutralization breadth and potency. (**A**) Summary of neutralization data in Figure 4A–B by each clade neutralized. Listed in colored boxes are percentage values. (**B**) Overall mutation frequencies of mAb combinations, which were calculated by overall number of nucleotide mutations in both heavy and light chains divided by combined heavy and light chain lengths. The mutation frequency of each heavy and light chain was calculated over both V- and J-genes.

With a mutation frequency of 5% in V_H_J_H_ and 9% in V_L_J_L_ (overall nt mutation frequency of 6%), the antibody 3H+3L nevertheless neutralized 46% of a broader panel of 74 viruses sensitive to PGT121/PGT124 with a median IC_50_ only 24- and 14-fold higher than PGT121 and PGT124, respectively (Figure 4A–B, Figure 5A and Figure S7, S8). With a few additional nucleotide mutations in the heavy chain (11% in V_H_J_H_, overall nt mutation frequency of 10%), the 32H+3L pair neutralized 80% of viruses that are sensitive to PGT124 with only 1.5-fold higher median IC_50_ than PGT124. The inferred intermediate for PGT121, 9H+3L, is 12% mutated in V_H_J_H_ (also 10% nt mutation frequency overall), but demonstrated less breadth and potency neutralizing 62% of PGT121-sensitive viruses with a 12-fold higher median IC_50_ than PGT121. Overall, the results confirm that, while SHM does enhance neutralization breadth and potency for this family of antibodies (Figure 4A–B), we are still able to observe relatively high levels of broad neutralization activity with substantially lower levels of SHM than found for the most affinity-matured antibodies.

To better evaluate the neutralization activity of the antibodies of intermediate divergence, 3H+3L and 32H+3L were tested on a 103 cross-clade virus panel in comparison with b12 and PGT121 (Figure S9, S10). b12 was one of the first CD4bs bNAbs isolated and demonstrates moderate neutralization breadth compared to more recently isolated bNAbs [3], [5]. The results show that the least mutated pair 3H+3L is 6% less mutated than b12, but demonstrated equivalent breadth (26%) and its median IC_50_ was still 3-fold more potent than b12 and 15-fold less potent than PGT121. The pair 32H+3L, meanwhile, was capable of neutralizing 48% of viruses on this panel and its median IC_50_ is 15-fold more potent than b12 and only 3-fold less potent than PGT121. PGT121 was capable of neutralizing 58% of viruses on this panel. Thus, while SHM does enhance neutralization breadth and potency for this family of antibodies, we are still able to observe relatively high levels of neutralization activity with substantially lower levels of SHM than the fully matured clones.

### Residues arising through SHM are important for neutralization at different stages of antibody maturation

In order to determine if residues in the variable region are a result of SHM or are due to polymorphisms in the donor, genomic DNA from the PGT121–123 donor was extracted and V and J genes from both heavy and the light chains were amplified and Sanger sequenced (Figure S11). Based on the results, there are no unique polymorphisms in the donor that distinguish the germline sequence from those reference sequences found in the IMGT database. Additionally, the sequence results indicate that the deletion in FR1 and the insertion in FR3 of the light chain, which are present in the PGT121–134 mAbs, were features that developed following VDJ or VJ recombination.

**Figure 6:**
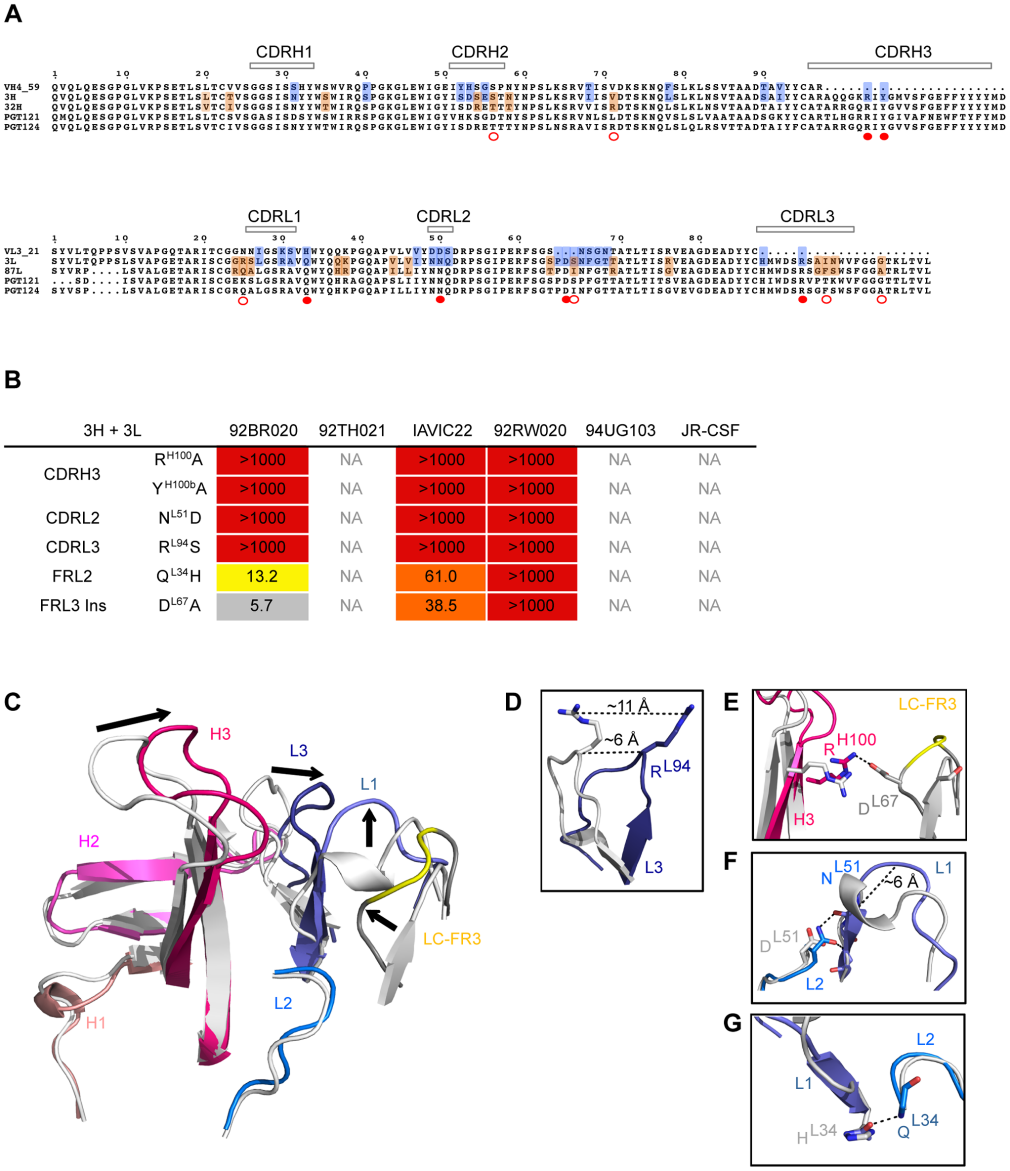
Role of somatic hypermutation in neutralization and antibody structure. (**A**) Residues from heavy chain intermediates and light chain intermediates were reverted to germline while indel and CDR3 residues were individually mutated to alanines and tested on a cross-clade virus panel. Highlighted in blue are residues that were reverted to germline. Highlighted in orange are residues reverted between intermediates. Filled red dots represent residues reversions that resulted in significant loss of neutralization as single reversions. Empty red dots represent residue reversions that resulted in significant loss of neutralization as pairwise reversions. Reported fold changes in IC_50_ are shown in Figure S12. (**B**) Reported fold changes in IC_50_ for residues reverted to germline in putative intermediate 3H+3L. (**C**) Residues that were found to be critical for neutralization in (**B**) were mapped on a putative PGT121 germline crystal structure. Global structural shifts were observed between germline (gray) and PGT121 (colored). Arrows indicate shifts of CDR loops. (**D**) 11 Å shift of R^L94^ in the CDRL3, which was identified to be important for neutralization activity. (**E**) Salt bridge between R^H100^ in the CDRH3 and D^L67^ in the FRL3 insertion that contributes to structural shifts. (**F**) Effect of N51D mutation in CDRL2. N^L51^ is highly conserved among all antibody variants and substitution completely abrogates neutralization. The introduction of two hydrogen bonds between the side chain of N^L51^ and backbone atoms might facilitate a helix to loop change in CDRL1. (**G**) A H34Q substitution in CDRL1 contributes to stabilizing the proximal positions of CDRL1 and CDRL2.

We next determined how individual mutations at different stages of antibody maturation enabled greater neutralization breadth or potency to gain insight into the most critical steps of SHM for this family of antibodies. For the antibody 3H+3L, single amino acids were individually reverted to the corresponding germline residue and residues comprising a framework light chain 3 (FRL3) insertion and CDR3 regions were individually substituted by alanine (Figure 6A–G). The parent 3H+3L mAb and mAb variants were then tested for neutralization on a 6-virus panel and the fold changes in IC_50_ are reported in Figure 6B and Figure S12. For the heavy chain of antibody 3H+3L, most of the neutralization activity for the majority of isolates is apparently mediated through the CDRH3; simultaneous reversion of all the heavy chain residues of 3H to germline, except for the CDRH3, resulted in loss of neutralization or reduced potency for only 20% of isolates (Figure S13). For the light chain of antibody 3H+3L, residues critical for neutralization activity were found in both framework and CDR regions, although these residues become less critical when paired with more mature heavy chains (Figure S14). Crystallographic studies of a putative PGT121 germline Fab, which is similar (0.4 Å core RMSD) to a previously reported crystal structure of another PGT121 putative germline antibody, suggested that the mutations from germline not only involved likely antigen contact residues, but also involved structural rearrangements in the heavy and, most notably, light chains as a result of somatic mutation in CDR and framework regions (Figure 6C–G, Figure S14, and Table S1) [39].

Having identified the residues mutated from germline that are most crucial to neutralization by antibody 3H+3L, we were interested in determining those that were most important to the increased breadth of antibodies 32H+3L and 3H+87L as compared to 3H+3L. Accordingly, the more mutated intermediate heavy chain 32H was reverted to the less mutated 3H heavy chain, paired with light chain 3L, and tested for neutralization breadth. A similar approach was taken for reversion of light chain 87L to 3L, paired with heavy chain 3H. Surprisingly, single amino acid changes did not show demonstrable effects on neutralization IC_50_ for the 32H reversion or for the 87L reversion (Figure S15). Strikingly, only variants for which at least two amino acids were reverted did we see a large increase in IC_50_ between parent and variant mAb in an isolate-specific manner. Such a pattern may present extra challenges to the guided evolution of an antibody response through vaccination.

**Figure 7:**
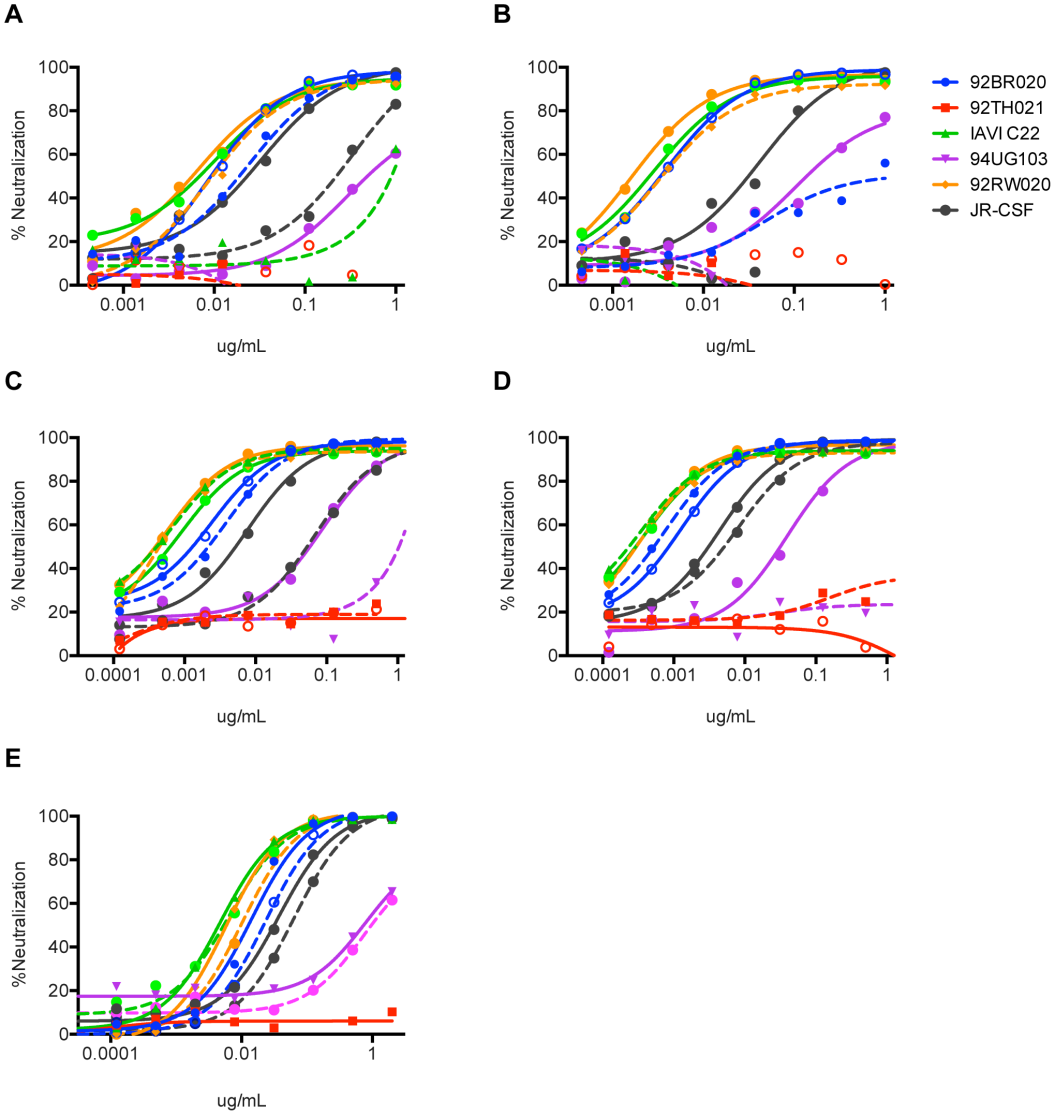
Neutralization activity of PGT122–133 mAbs without the FRL3 insertion and restoring the N-terminus deletion. The three amino acid insertion in FR3 of the light chain of (**A**) PGT122, (**B**) PGT123, (**C**) PGT124, and (**D**) PGT133 was removed and paired with the corresponding heavy chains and tested for neutralization activity on a 6-virus panel. Solid lines represent wild-type antibodies with the insertion and dashed lines represent the antibodies without the FR3 insertion. (**E**) The 7 amino acid N-terminus sequence of IGHV4-59 germline was placed back into the N-terminus of PGT121 and tested for neutralization breadth and potency on a cross-clade 6 virus panel. Solid lines represent WT PGT121 (with N-terminal deletion) and dashed lines represent PGT121 with the N-terminus restored.

To gain further insight into the effects of the insertion and deletions on neutralization activity, we tested neutralization breadth and potency of the PGT122–133 antibodies without the FRL3 insertion and restoring the framework light chain 1 (FRL1) deletion on a cross-clade 6 -virus panel. Upon removal of the FRL3 insertion, we observed a partial loss of neutralization in an isolate-specific manner that varied somewhat among the somatic variants of the lineage (Figure 7A–D). The isolate 92BR020, for example, became resistant to neutralization by PGT123 lacking the FRL3 insertion, but was still sensitive to the corresponding PGT122, PGT124, and PGT133 variants. Meanwhile, the isolate IAVI C22 became resistant to PGT122 lacking the FRL3 insertion, but remained sensitive to PGT123, PGT124, and PGT133 variants. Notably, the isolate 92RW020 remained sensitive to all the antibodies in the absence of the FRL3 insertion. These results suggest that the FRL3 insertion is beneficial but not absolutely crucial for neutralization breadth and that the antibody maturation process may have proceeded some way before the insertion occurred. These results also argue that, if an immunogen is capable of eliciting this lineage of antibodies without the FRL3 insertion, neutralization breadth may be achieved through the complementary neutralization profiles of somatic variants. Finally, we were unable to find large effects on neutralization breadth or potency upon restoration of the 7-amino acid N-terminus deletion in FRL1 suggesting that this feature is not crucial for bnAb activity (Figure 7E).

### Intermediate antibody binds cell surface Env more effectively than monomeric gp120 and shows greater dependence on recognition of multiple glycans than mature PG121

**Figure 8:**
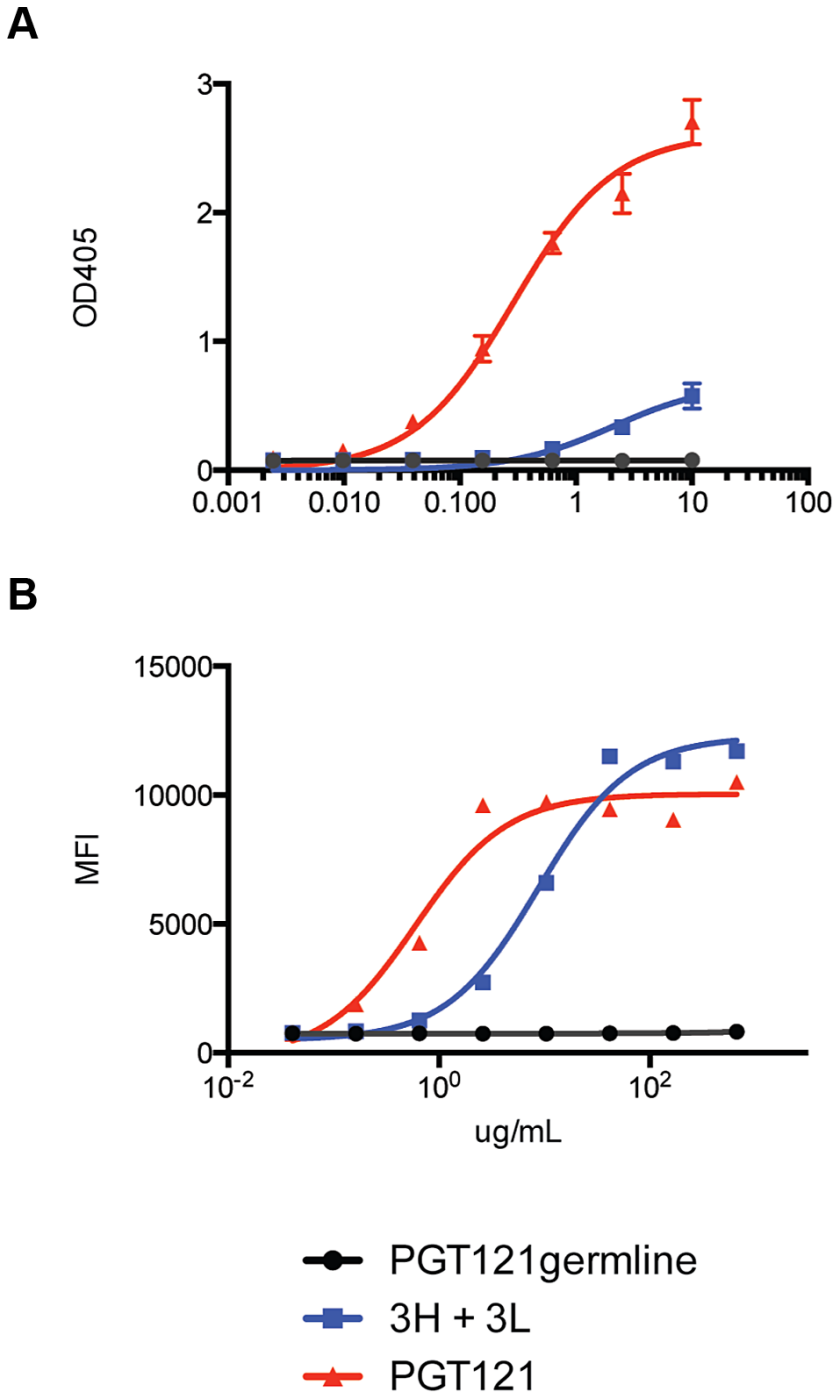
Putative germline of PGT121 does not bind monomeric gp120 or cell surface Env. (**A**) PGT121germline does not bind recombinant gp120 (92BR020). Recombinant gp120s were produced in 293F cells and purified by lectin column before use in ELISA binding assays. ELISA values are reported in optical density at 405 nm (OD405). (**B**) PGT121germline does not cell surface Env (92BR020). Cell surface Env was produced by transfecting pseudovirus in 293T cells and binding was measured by flow cytometry (reported in mean fluorescence intensity or MFI).

**Figure 9:**
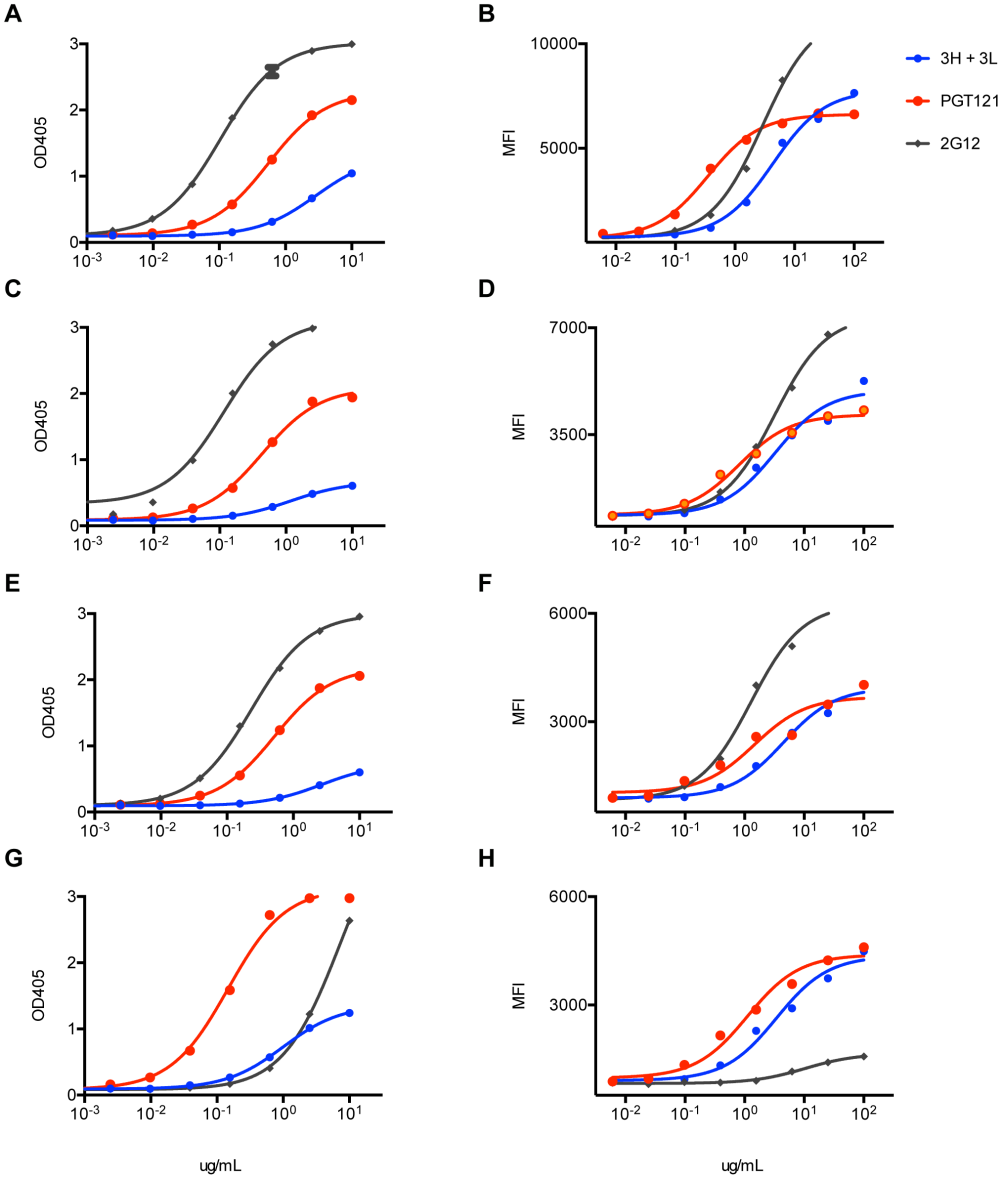
Inferred intermediate antibodies preferentially bind native Env relative to monomeric gp120. mAbs 3H+3L (blue) and PGT121 (red) were tested for binding by ELISA to monomeric gp120, which was extracted from lysed virus supernatants: (**A**) 92BR020, (**C**) 92RW020, (**E**) JR-FL E168K/N192A, (**G**) IAVI C22. Antibodies were also tested for cell surface Env binding by flow cytometry: (**B**) 92BR020, (**D**) 92RW020, (**F**) JR-FL E168K/N192A, (**H**) IAVI C22. mAb 2G12 was included as a control (gray). ELISA values are reported as optical density at 405 nm (OD405) and flow cytometry values are reported as mean fluorescence intensity (MFI).

**Figure 10:**
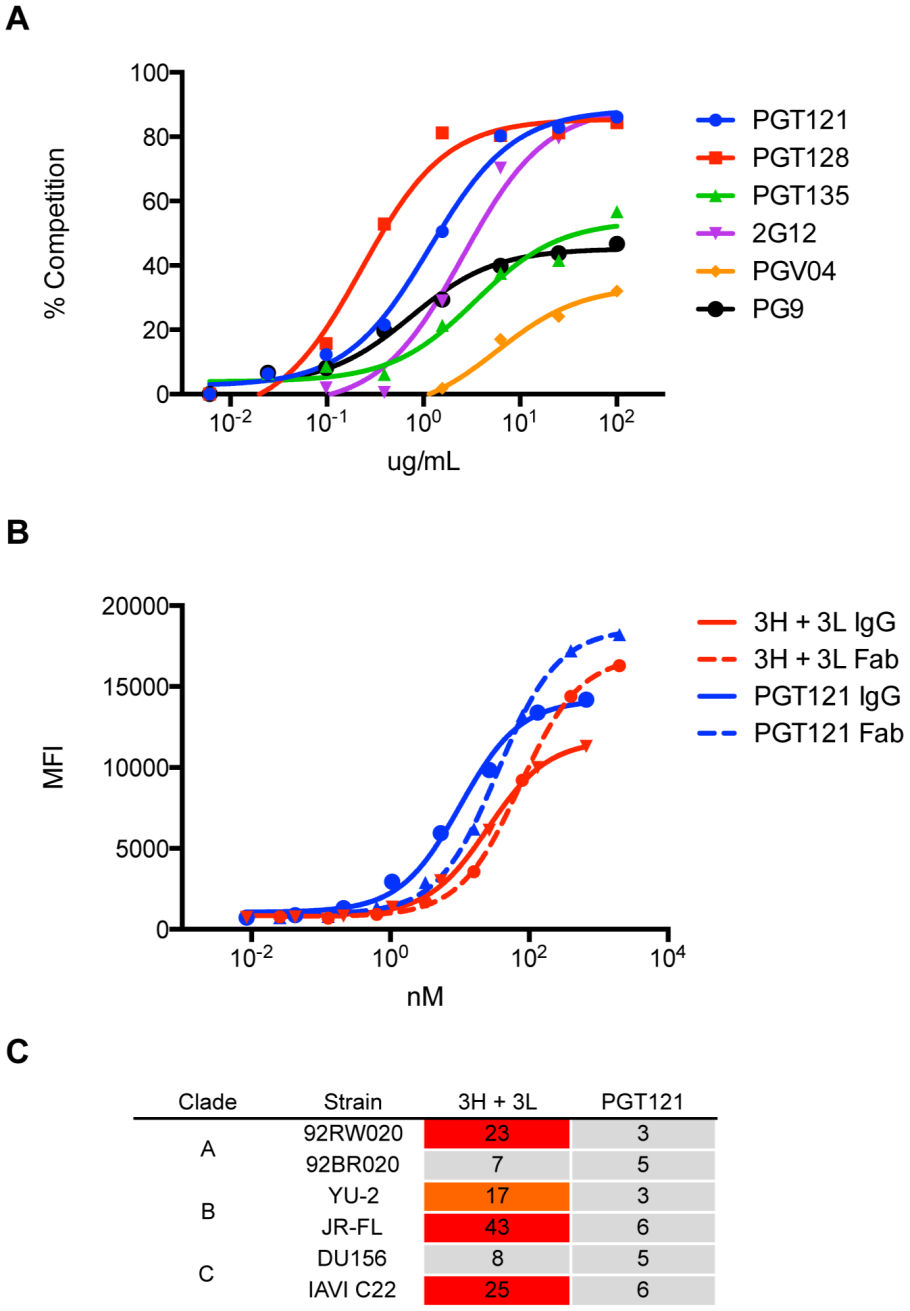
Antibody 3H+3L likely crosslinks between trimers. (**A**) 3H+3L binds an epitope overlapping with those of PGT121, PGT128, and 2G12 as shown by competition of biotinylated antibody 3H+3L with an antibody panel. Binding assays were performed by flow cytometry on JR-FLΔCT isolate transfected in 293T cells. (**B**) IgG and Fab fragments were tested for binding on JR-FLΔCT isolate expressed on transfected 293T cells and no substantial differences in avidity were observed. Solid lines represent IgG and dashed lines represent Fab fragments. (**C**) Purified IgGs of 3H+3L and PGT121 were digested into Fab fragments using Lys-C, purified, and tested for neutralization on a cross-clade panel. Loss of neutralization was found for 3H+3L Fab, but not for PGT121 Fab. Reported values are IC_50_ ratios of Fab compared to IgG using the equation: (IC_50_ Fab)∶(IC_50_ IgG).

Many research groups have generated germline derivatives or close equivalents (unmutated germline ancestors) of known bnAbs, but these constructs do not bind most recombinant gp120s or neutralize most virus strains [23], [25], [26], [40]. Indeed, the putative germline of PGT121 was unable to bind gp120 (Figure 8A–B). However, the intermediate 3H+3L antibody did bind weakly to monomeric gp120 from multiple isolates. Most notably, the 3H+3L antibody bound to monomeric gp120 with notably lower apparent affinity than to trimeric cell surface Env in contrast to mature PGT121, which bound with similar apparent affinities to monomeric gp120 and trimeric Env (Figure 9A–B and Figure S16). We confirmed by competition assays that, despite these binding differences, antibodies 3H+3L and PGT121 do share overlapping epitopes (Figure 10A). To determine if the preference of antibody 3H+3L for trimeric Env was due to avidity effects, we generated Fab fragments and tested neutralization activity and binding by flow cytometry. The results demonstrate that 3H+3L loses neutralization potency as an Fab compared to IgG, despite little difference in apparent affinity for cell surface trimer between Fab and IgG (Figure 10B–C). Conversely, PGT121 maintains neutralization potency as a Fab. These results suggest that the early intermediate antibodies may be crosslinking between Env on virions in order to achieve neutralization [7], but the lineage becomes less dependent on crosslinking following affinity maturation. Overall, these results indicate that the PGT121–134 antibody lineage may have been selected for native Env binding at some point during maturation, possibly on whole virions.

**Figure 11:**
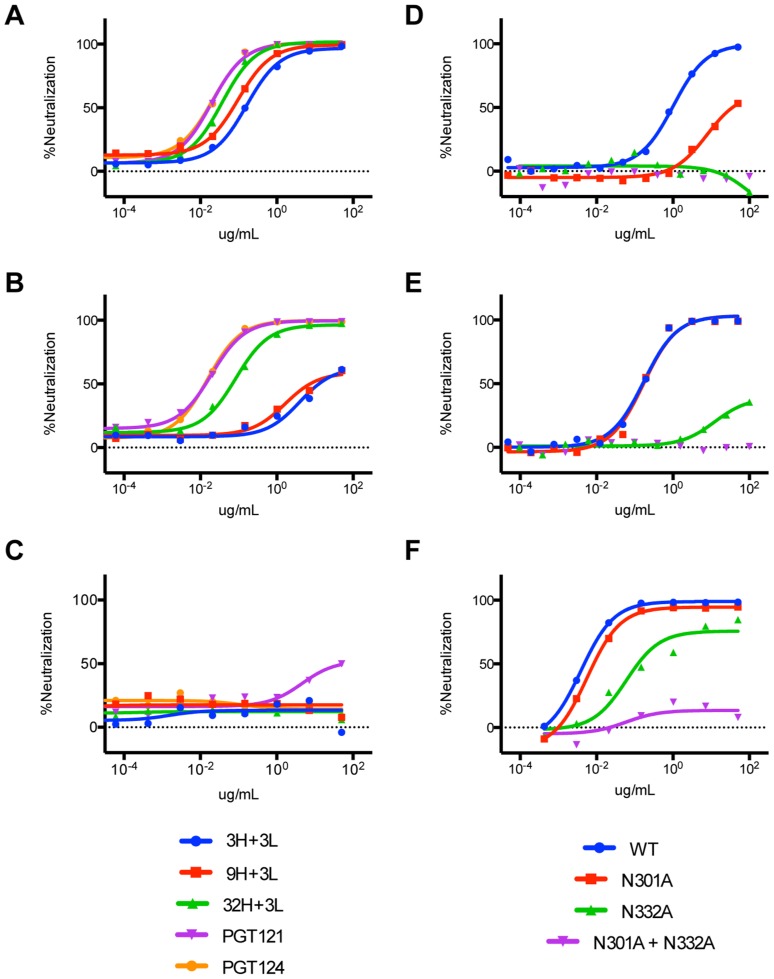
Neutralization assays on JR-FL glycan mutants indicate binding of inferred intermediate antibodies to both N301 and N332 on HIV-1 Env. (**A**) Neutralization curves of inferred intermediate antibodies on wild-type JR-FL virus (**B**) JR-FL N301A mutant virus, and (**C**) JR-FL N332A mutant virus. (**D**) Neutralization curves of 3H+3L and (**E**) PGT121 on wild-type JR-FL virus compared to single and double glycan mutant viruses. (**F**) Neutralization curves of PGT121 on wild-type 92BR020 virus compared to single and double glycan mutant viruses.

Previous work identified only the N332 glycan as critical for PGT121 neutralization and binding [5], [41]. However, because all of the other known glycan-binding antibodies require concomitant binding of adjacent glycans, we tested our putative precursors for involvement of other glycans in neutralization [5], [7], [42]–[44]. Reduced neutralization on N301A viruses and complete loss of neutralization on N332A viruses was found for 3H+3L, 32H+3L, and 9H+3L mAbs (Figure 11A–C). For PGT121, we observed no effects for N301A viruses, a substantial loss of neutralization for N332A viruses, and an even greater loss of neutralization for N332A+N301A mutant viruses (Figure 11D–F). Overall, the results suggest that early antibodies of the PGT121–123 lineage are dependent on both N301 and N332 glycans and that the dependency on the N301 glycan is reduced upon maturation.

## Discussion

The conclusions described here can help guide immunogen design to elicit PGT121-like antibodies. Specifically, the presentation of native Env instead of monomeric gp120 in germinal centers appears to be important for the maturation of this family of antibodies. This preference for native Env over monomeric gp120 may be due to avidity effects or to the presentation of epitope residues on native Env that are not fully recapitulated on monomeric gp120. Additionally, we observed an evolution in the dependency of glycan binding between putative intermediates and fully mature antibody. As conventional protein-glycan interactions are usually in the micromolar range, the avidity offered by binding to multiple glycans may be an important factor in triggering glycan-dependent bnAb lineages. By comparing the differences in glycan contact residues between putative precursor and affinity-matured antibody, it may be possible to rationally target residues for maturation in order to achieve greater neutralization breadth.

Compared to less-mutated bnAbs that target the CD4 binding site [27] the less-mutated intermediates presented here are notably broader and more potent suggesting potentially important differences between the CD4 binding site and the N332 region as vaccine targets that may give some advantage to the latter region. One possible explanation is that antibodies targeting the CD4bs may require more SHM in order to fold into specific conformations that enable binding into the CD4bs pocket. The epitope that PGT121 binds to, on the other hand, may be more exposed and may accommodate different antibody binding angles [7], [11], [37], [38]. Thus, the PGT121 lineage may not necessarily require extensive levels of SHM in order to bind its epitope. It would likely be informative to perform similar analyses for other known bnAbs in order to better define the minimal amount of mutations necessary to achieve neutralization breadth and potency for different bnAbs.

Despite the lower levels of SHM associated with PGT121-like, more VRC01-like antibodies than PGT121-like bnAbs have been isolated from various donors. This apparent discrepancy may be due to the fact that many B cell selection studies have used a bait (RSC3) engineered for isolating VRC01-like antibodies . Additionally, there are many donors in various cohorts who show N332 serum reactivity whose antibodies have not yet been isolated and thus new PGT121-like antibodies may be forthcoming. Finally, antibodies targeting the N332 epitope appear to derive from several V_H_D_H_J_H_ and V_L_J_L_ combinations, while VRC01-class antibodies utilize only a single V_H_ germline gene, the V_H_1-2 gene. Thus, given the greater diversity of antibody responses targeting the N332 epitope, the probability of identifying an exact or highly similar PGT121 response from other infected donors may be low.

Importantly, as shown here, deep sequencing and phylogeny with ImmuniTree has the potential to identify intermediate antibodies that may not be as broadly neutralizing as some individual affinity-matured Abs, but have considerably lower levels of SHM. In this regard, considerable neutralization breadth may be achieved by eliciting less-mutated antibodies with complementary neutralization profiles, probably targeting a range of epitopes. Such an approach may circumvent the need for long-term prime/boost strategies, which are likely required to elicit high levels of SHM and suggests that, given the right immunogens, more conventional vaccination strategies than have been contemplated may be sufficient for an HIV vaccine.

Strikingly, it appears that higher levels of SHM concurrently enhance neutralization breadth and increase potency against the majority of isolates recognized by putative precursors (Figure 4A–B and Figure S7, S8). These observations are consistent with the hypothesis that SHM results in some antibodies becoming both very broad and potent by “homing in” on a conserved site of vulnerability (“sweet spot”) from a response that initially recognizes a somewhat different footprint on the virus Env. One hypothesis suggests that Env escape variants drive maturation to avoid variable residues and/or glycans adjacent to the most conserved residues by directing contacts to converge on the smallest and most conserved binding site on Env (compare ref. [45]). An alternative hypothesis suggests that the antibody matures to avoid variable residues and/or glycans without substantially changing the size of its footprint but by refining its angle of approach (compare ref. [46]). By either of these mechanisms, antibody selection can occur on the basis of affinity for diverse isolates with a concomitant increase in neutralization breadth and potency (Figure 4A–B and Figure S17). Indeed, calculations of an antigen-antibody interaction model are consistent with the idea that bnAbs are characterized as antibodies that form a small number of stronger interactions with conserved residues compared to antibodies that are able to make many contacts with many variable residues (Figure S18). Further dynamic modeling of antibody SHM in response to Env escape in concert with targeted immunization studies will help to design vaccines and vaccine strategies that hone antibody responses onto the the PGT121 epitope.

## Materials and Methods

### Ethics statement

Peripheral blood mononuclear cells (PBMCs) were obtained from donor 17, an HIV-1 infected donor from the IAVI Protocol G cohort [36]. All human samples were collected with written informed consent under clinical protocols approved by the Republic of Rwanda National Ethics Committee, the Emory University Institutional Review Board, the University of Zambia Research Ethics Committee, the Charing Cross Research Ethics Committee, the UVRI Science and Ethics Committee, the University of New South Wales Research Ethics Committee. St. Vincent's Hospital and Eastern Sydney Area Health Service, Kenyatta National Hospital Ethics and Research Committee, University of Cape Town Research Ethics Committee, the International Institutional Review Board, the Mahidol University Ethics Committee, the Walter Reed Army Institute of Research (WRAIR) Institutional Review Board, and the Ivory Coast Comité National d'Ethique des Sciences de la Vie et de la Santé (CNESVS).

### 454 sequencing library preparation

Reverse transcription was performed with 10 µL total RNA and 2 µL RT primer mix (50 µM oligo-dT and 25 µM random hexamer). The mixture was heated at 95°C for 1 min, 65°C for 5 min, then cooled on ice for 1 min. For each reaction, a mix was prepared with 4 µL 5× FS buffer, 1 µL 10 mM dNTP mix, 1 µL 0.1 M DTT, 1 µL RNase inhibitor (Enzymatics), and 1 µL SuperScript III RT (Invitrogen). This mix was added to the reaction and incubated at 25°C for 10 min, 35°C for 5 min, 55°C for 45 min, and 85°C for 5 min. RNA/DNA hybrid was removed by adding 4 µL E. coli RNase H (Enzymatics). PCR reactions were assembled using 13.75 µL water, 5 µL cDNA, 5 µL 5× HF buffer, 0.5 µL 10 mM dNTP, 0.25 µL of each 100 µM primer stock, and 0.25 µL Phusion Hot Start. The reaction was cycled at 98°C (60 s), 24 cycles of 98°C (10 s), 62°C (20 s), and 72°C (20 s), with a final extension at 72°C (5 min). Samples were purified on a QIAquick column and run on a 2% agarose E-gel. The desired bands were purified using the Qiagen MinElute gel extraction kit, eluted twice with 10 µL EB buffer, and quantitated on a 2100 Bioanalyzer. Samples were sent to SeqWright for 454 sequencing, which was performed per manufacturer's instructions.

### Pseudovirus production and neutralization assays

To produce pseudoviruses, plasmids encoding Env were co-transfected with an Env-deficient genomic backbone plasmid (pSG3ΔEnv) in a 1∶2 ratio with the transfection reagent Fugene 6 (Promega). Pseudoviruses were harvested 72 hours post transfection for use in neutralization assays. Neutralizing activity was assessed using a single round of replication pseudovirus assay and TZM-bl target cells, as described previously [5].

### Cell sorting and RNA extraction

Frozen vials of 10×10^6^ PBMCs were thawed and washed before staining with Pacific Blue labeled anti-CD3 (UCHT1), Pacific Blue labeled anti-CD14 (M5E2), FITC labeled anti-CD19 (HIB19), PE-Cy5 labeled anti-CD10 (HI10a), PE labeled anti-CD27 (M-T271), and APC labeled anti-CD21 (B-ly4), all from BD Biosciences. Sorts were performed on a high speed BD FACSAria into miRVana lysis buffer (Ambion). Immature B cells, exhausted tissue-like memory, activated mature B cells, resting memory B cells, and short-lived peripheral plasmablasts were stained using previously described markers [47]. Total RNA from the cells was then extracted using the mirVana RNA extraction kit (Ambion) according to manufacturer's instructions and quantitated on a 2100 Bioanalyzer (Agilent).

### Antibody and protein expression and purification

Antibody sequences were synthesized and cloned into previously described heavy and light chain vectors [3], [48]. The plasmids were co-transfected (1∶1 ratio) in either HEK 293T or 293 FreeStyle cells using Fugene 6 (Promega) or 293fectin (Invitrogen), respectively. Transfections were performed according to the manufacturer's protocol and antibody supernatants were harvested four days following transfection. Antibodies produced in 293T cells were quantified by ELISA and used directly in neutralization assays. Antibodies produced in 293 freestyle cells were further purified over a protein A column. Mutations were introduced by site-directed mutagenesis using the QuikChange site-directed mutagenesis kit (Stratagene). Recombinant gp120 proteins were transfected in 293 FreeStyle cells using 293fection (Invitrogen) and purified with *Galanthus nivalis* lectin column followed by size exclusion using Superdex 300 26/60 (GE Healthcare).

### Cell surface binding assays

Titrating amounts of mAbs were added to HIV-1 Env-transfected 293T cells and incubated for 1 h at 4°C in 1× PBS. Following washing, cells were fixed with 2% PFA (PolySciences) for 20 min at RT. The cells were then washed and stained with a 1∶200 dilution of phycoerythrin-conjugated goat anti-human IgG F(ab′)2 (Jackson) for 1 h at RT. Binding was analyzed using flow cytometry. Binding competitions were performed by titrating amounts of competitor monoclonal antibodies before adding biotinylated antibody at a concentration required to give IC_70_ and then measuring binding with phycoerythrin-labeled streptavidin (Invitrogen). FlowJo software was used for data interpretation.

### ELISA assays

Binding by ELISA was performed as described previously [5]. Briefly, plates were coated with goat anti-human IgG Fc (Pierce) or with gp120 and binding was detected using goat anti-human IgG F(ab′)2 conjugated to alkaline phosphatase (Pierce). For binding to gp120 extracted from lysed virions, plates were coated with 5 ng/uL of sheep D7324 anti-gp120 antibody (Aalto Bio reagents). Virus supernatents were lysed using a final concentration of 1% NP-40 and incubated on coated plates for 2 h at 37°C. Detection was measured using goat anti-human IgG F(ab′)2 conjugated to alkaline phosphatase (Pierce). Antibody concentration was calculated by linear regression using a standard concentration curve of purified IgG protein.

### PGT121 germline Fab expression, purification, crystallization and X-ray diffraction analysis

PGT121germline Fab was produced in HEK 293T cells and purified as previously described [7]. Briefly, three days after transfection with the heavy and light chain genes, the expression media was harvested and the secreted Fab was purified via an anti-human λ light chain affinity matrix (CaptureSelect Fab λ; BAC), followed by cation exchange chromatography and size-exclusion chromatography. X-ray diffraction quality crystals were obtained in a condition containing 0.2 M magnesium acetate, 20% w/v PEG 8000, 0.1 M sodium cacodylate, pH 6.5.Before mounting and flash freezing the crystals in liquid nitrogen, the mother liquor was supplemented with 20% glycerol for cryo-protection. A complete datasetwas collected at the APS 23-ID beamline. Data processing was performed using XDS [49]. The PGT121germline structure was solved using PHASER in space groups P2_1_2_1_2_1_ with the PGT123 Fab structure as a search model [50]. Refinement was performed using a combination of PHENIX and COOT [51], [52]. Refinement statistics for the final models are reported in Table S1.

### Raw data processing: VDJ alignment and clone definition

Raw sequencing data were processed using in-house tools written in python. Reads were split into barcodes, size-filtered, and aligned to IMGT's germline VDJ reference database. The scores were kept low, as we were interested in sequences that were very highly mutated. The V region is aligned first, then removed, followed by J, then removed, followed by D. The IMGT-defined CDR3 sequence of each read was then extracted. The CDR3 sequences were sorted by abundance and clustered with USEARCH5.1 with the options “–minlen 0 –global –usersort –iddef 1 –id 0.9”. Finally, each CDR3 sequence was aligned to the “target” antibody sequences of PGT121–123 to determine a “divergence” value from these antibodies.

### Antibody variant identification and analysis

The divergence-mutation plots are used as a tool to identify reads that are similar to the known PGT121–123 antibodies. High-identity clusters of sequences and clusters that are above background are manually identified and used as input for a phylogeny inference with Immunitree. Immunitree implements a Bayesian model of somatic hypermutation of clones, including probabilistic models of SHM and sequencing error and performs Markov chain Monte Carlo over the tree structure, birth/death times of the subclones, birth/death, mutation, and sequencing error rates, subclone consensus sequences, and assignment of reads to nodes. The tree structure is also used for multiple computations and to overlay different information. We estimate the selection pressure that a given node has experienced using the BASELINe algorithm. It performs a Bayesian estimation of selection pressure by comparing the observed number of replacement/silent mutations in the CDRs/FWRs of the node consensus sequence.

## Supporting Information

Figure S1 **PGT121–134 heavy chain divergence plots.** Data generated from 454 sequencing were graphed in identity (PGT bNAb) vs. divergence (germline) plots. Each color represents a unique clone, which was determined using the procedures outlined in the methods section. Divergence plots were created for (**A**) PGT121, (**B**) PGT122, (**C**) PGT123, (**D**) PGT124, (**E**) PGT133, and (**F**) PGT134.
(TIF)

Figure S2 **PGT121–134 light chain divergence plots.** Data generated from 454 sequencing were graphed in identity (PGT bNAb) vs. divergence (germline) plots. Each color represents a unique clone, which was determined using the procedures outlined in the methods section. Divergence plots were created for (**A**) PGT121, (**B**) PGT122, (**C**) PGT123, (**D**) PGT124, (**E**) PGT133, and (**F**) PGT134.
(TIF)

Figure S3 **ImmuniTree without 454 sequencing error correction.** (**A**) Heavy chain SHM phylogeny inferred by the ImmuniTree algorithm without correcting for 454 sequencing errors. (**B**) Light chain SHM phylogeny inferred by the ImmuniTree algorithm without correcting for 454 sequencing errors. Trees were generated using the same set of sequences to build the trees in Figure 2.
(TIF)

Figure S4 **Neutralization score of each pair tested on 6-virus panel.** Each antibody of different heavy and light chain pairs was produced in 293T cells and tested for neutralization activity on a cross-clade 6-virus panel. Neutralization score was calculated using the formula: mean(log_10_(10/IC_50_)). Boxes were colored as follows: score≤1.7, yellow; 1.8≤score ≤2.5, orange; score>2.5, red.
(TIF)

Figure S5 **Nodes can be classified as more PGT121-like or more PGT124-like based on ImmuniTree clustering.** (**A**) Heavy chain nodes selected for pairing and characterization are shown divided into PGT121-like or PGT124-like branches based on ImmuniTree. (**B**) Light chain nodes selected for pairing and characterization are shown divided into PGT121-like or PGT124-like branches based on ImmuniTree.
(TIF)

Figure S6 **Comparison of neutralization profile between PGT121 and PGT124.** PGT121 and PGT124 were tested on 87 cross-clade isolates to determine neutralization breadth and potency. Listed in colored boxes are IC_50_ values (µg/ml) of each isolate neutralized.
(TIF)

Figure S7 **Inferred intermediates of PGT121 were evaluated for neutralization breadth and potency.** Heavy and light chain nodes leading to mAb PGT121 were paired and tested on a 74-virus panel of PGT121-sensitive viruses. Listed in colored boxes are IC_50_ values (µg/ml) of each isolate neutralized.
(TIF)

Figure S8 **Inferred intermediates of PGT124 were evaluated for neutralization breadth and potency.** Heavy and light chain nodes leading to mAb PGT124 were paired and tested on a 74-virus panel of PGT124-sensitive viruses. Listed in colored boxes are IC_50_ values (µg/ml) of each isolate neutralized.
(TIF)

Figure S9 **Neutralization table summary of putative intermediates in comparison to b12.** Putative intermediates 3H+3L and 32H+3L were tested in comparision to PGT121 and b12 in TZM-bl neutralization assays on a cross-clade 103 pseudovirus panel. Listed in colored boxes are percentages of each clade neutralized. Boxes are colored as follows: percent of viruses neutralized <25% (green), percent of viruses neutralized: 25–50% (yellow), percent of viruses neutralized: 50–100 90% (red). ^a^Mutation frequency was calculated over the V-gene and J-gene as nucleotides (nt) or amino acids (aa) differing from the putative germline sequence. The CDR3 regions and insertions and deletions were excluded from the analysis.
(TIF)

Figure S10 **Neutralization panel of putative intermediates in comparison to b12.** Inferred intermediates 3H+3L, and 32H+3L were tested in comparision to PGT121 and b12 in TZM-bl neutralization assays on a cross-clade 103 pseudovirus panel. Listed IC_50_ values are in ug/mL.
(TIF)

Figure S11 **Alignment of intermediate and mature antibodies to germline.** Genomic DNA from the PGT121–123 donor was extracted from CD4+ T cells, which were enriched through anti-CD4 antibody coated magnetic beads. (**A**) Primers designed to amplify the IGHV4-59 and the IGHJ6*03 gene families were used to generate heavy chain libraries. (**B**) Primers designed to amplify the IGLV3-21 and IGLJ3*02 gene families were used to produce light chain libraries. Both libraries were then TOPO cloned into vectors and individual colonies were subsequently Sanger sequenced to determine germline sequences. Alignments were made using ClustalW.
(TIF)

Figure S12 **Paratope mapping of 3H and 3L paired with heavy and light chains of different maturation levels.** Alanine scanning mutagenesis of heavy chain 3H paired with less-mutated light chain 3L and highly-mutated light chain 87L as well as alanine scanning mutagenesis of 3L paired with less-mutated 3H, moderately-mutated 32H, and highly-mutated PGT121H. Values represent fold-changes in IC_50_ using formula: Mutant(IC_50_)/WT(IC_50_). Empty gray boxes represent isolates for which the antibody did not neutralize
(TIF)

Figure S13 **Reversion of all residues in 3H except CDRH3 results in loss of neutralization for 20% of isolates.** Alanine scanning mutagenesis of heavy chain 3H paired with 3L were tested in TZM-bl neutralization assays on viruses previously determined to be neutralized by 3H+3L. Listed IC_50_ values are in ug/mL.
(TIF)

Figure S14 **Many of the residues from SHM may play a role in stabilizing antibody structure and conformation.** Structural representation of the mutations that occurred in PGT123 during affinity maturation from a putative germline antibody. Residues thought to have mutated are shown in blue and red for the light and heavy chains, respectively. Mutations are seen to have taken place across all regions of the Fv domain and contribute to both paratope evolution and antibody architecture.
(TIF)

Figure S15 **Single and double mutant summary of inferred intermediate antibodies 3H+87L to 3H+3L and 32H+3L to 3H+3L.** Reversion of single and double residues of light chain 87L to light chain 3L and paired with heavy chain 3H. WT and reverted mutants were tested on isolate (**A**) 1006_11_C3_1601 and (**B**) SC422661.8. (**C**) Reversion of single and double residues of heavy chain 32H to heavy chain 3H and paired with light chain 3L. WT and reverted mutants were tested on isolate JR-CSF. Values represent fold-changes in IC_50_ using formula Mutant(IC_50_)/WT(IC_50_). ND represent double mutants that were not determined.
(TIF)

Figure S16 **The least mutated antibody variant 3H+3L binds recombinant gp120 less well than the affinity mature PGT121.** Recombinant gp120s were produced in 293F cells and purified by lectin column before use in ELISA binding assays. ELISA values are reported in optical density at 405 nm (OD405).
(TIF)

Figure S17 **Illustration of role of somatic hypermutation in the development of neutralization breadth.** Models for development of neutralization breadth via SHM by targeting the “sweet spot” on HIV Env. Breadth can either develop by converging onto or by reorienting onto a highly conserved region on HIV Env as illustrated. This convergence or reorientation is directed by virus escape mutations, which select for antibodies that interact with conserved regions (glycans, conserved side chains and main chain atoms) and less dependency on variable residues (variable side chains). By this process, affinity selection can lead to greater neutralization breadth and potency.
(TIF)

Figure S18 **In silico experiments show that bNAbs form stronger contacts with conserved sites than with variable sites on Env.** Each bNAb is represented by a pair of points, one red point and one blue point. The red point represents the average interaction strength of residues on the bNAb with variable sites of the antigen, and the blue point represents the average interaction strength with conserved antigen sites. The 63 red and blue points shown correspond to the 63 bNAbs that emerged in silico. The purple bars indicate the mean value averaged over all the selected antibodies. A significant difference between the mean values (purple bars) is apparent (*P*<0.001).
(TIF)

Figure S19 **A draw from the generative model of a synthetic clone producing 1000 reads.** Each node is a cell. The size of a cell is proportional to the number of reads it produced (with noise). which is also listed next to it. Each edge is a birth event and next to it is listed the mutation distance between parent and child. Cells with identical sequences are marked with same color.
(TIF)

Figure S20 **A binary tree by FastTree 2.0.** A binary tree was generated using 1000 synthetic reads of Figure S19 and visualized using Archeopteryx.
(TIF)

Figure S21 **Mutation trees for the synthetic clone are obtained by collapsing edges marked with 0.** (**A**) The true mutation tree for Figure S1. For tracking purposes, we color the reads of each cell by the cell's color. (**B**) The mutation tree returned by running ImmuniTree on the 1000 synthetic reads. Each cell is a pie chart showing the color composition of its reads. Almost each node in the reconstructed tree retained its identity, and the trees have an almost identical topology.
(TIF)

Figure S22 **Comparison of the average missing-branch (** ***k*** **) rate for different values of** ***k*** **between FastTree and ImmuniTree over 100 synthetic clones.** Comparison of the average missing-branch(k) rate, for different values of k, between FastTree and ImmuniTree, over 100 synthetic clones. From each synthetic clone we simulated 1000 reads, and let ImmuniTree and two other tree construction methods (FastTree and MrBayes) reconstruct the true tree that generated those reads. Each tree branch induces a partition of the reads into two sets (based on which side of the branch they are at). For each branch of the true tree we found the branch in the reconstructed tree with the minimum number of reads that need to switch sides in order for the induced partitions to match. Missing-branch(k) is the fraction of branches in the true tree for which the above minimum was greater than k.
(TIF)

Figure S23 **An illustration of the generative model.** First, a tree of cells is generated, each cell has a parent cell, a birth time and a death time. Then, the sequences of these cells are filled in from top to bottom, each sequence is a copy of its parent, with a slight chance of mutation. Last, reads are generated from the cells alive at the end of the process, with added sequencing noise.
(TIF)

Figure S24 **A subgraph of an internal node.** Each horizontal line is a cell. Let the top cell be called R. v is a birth event in R's life, and nodes u and w are the previous and next events (we assume here they are both birth events). The nodes f, e, b, c represent the next event in each cell's life, either birth or death. Similarly, the node a represents the event preceding u on R's life, which could be either another birth or R's own birth. One of our MCMC moves proposes to disconnect the edge v→c from the tree, and reconnect it to a new position on this subgraph.
(TIF)

Figure S25 **Comparison between binary tree and output from ImmuniTree.** (**A**) A binary tree sampled from the algorithm. Horizontal lines represent lifetimes of cells. Vertical lines represent birth events. Numbers indicate the mutation distance between the child and its parent (0 if not indicated). Cells with identical sequences are marked by the same color. (**B**) The reported canonical tree after collapsing identical sequences to a single node (of matching color). Number inside the nodes indicate the number of reads associated with that node.
(TIF)

Table S1 **X-ray crystallography statistics of PGT121germline.**
(TIF)

Text S1 **Supplementary text describing ImmuniTree.** Description of a novel Bayesian method called ImmuniTree, which models antibody somatic hypermutation while accounting for sequencing error. Supplementary text includes *in silico* experiments and additional details on how the algorithm was designed.
(PDF)
