# Correction: The Effects of Somatic Hypermutation on Neutralization and Binding in the PGT121 Family of Broadly Neutralizing HIV Antibodies

**DOI:** 10.1371/annotation/f1f8c791-61e9-45c6-a455-92c6dadf9f06

**Published:** 2013-12-30

**Authors:** Devin Sok, Uri Laserson, Jonathan Laserson, Yi Liu, Francois Vigneault, Jean-Philippe Julien, Bryan Briney, Alejandra Ramos, Karen F. Saye, Khoa Le, Alison Mahan, Shenshen Wang, Mehran Kardar, Gur Yaari, Laura M. Walker, Birgitte B. Simen, Elizabeth P. St. John, Po-Ying Chan-Hui, Kristine Swiderek, Stephen H. Kleinstein, Galit Alter, Michael S. Seaman, Arup K. Chakraborty, Daphne Koller, Ian A. Wilson, George M. Church, Dennis R. Burton, Pascal Poignard

In the author list, the spelling of Stephen H. Kleinstein's first name is incorrect.

The correct spelling is Steven H. Kleinstein.

This author also has an additional affiliation that is missing from the published article. The affiliation is:

Interdepartmental Program in Computational Biology and Bioinformatics, Yale University, New Haven, CT, USA.

Table S1 in the published article does not include a PDB code. Please download the new version of Table S1, which contains the PDB code, at the following link: 

.

The PDB ID is 4NPY. 

