# Supplementary figures and images for: The Effects of Somatic Hypermutation on Neutralization and Binding in the PGT121 Family of Broadly Neutralizing HIV Antibodies
Source: PLoS Pathog. 2013 Nov 21;9(11):e1003754. doi: 10.1371/journal.ppat.1003754 (PMC3836729; doi:10.1371/journal.ppat.1003754)

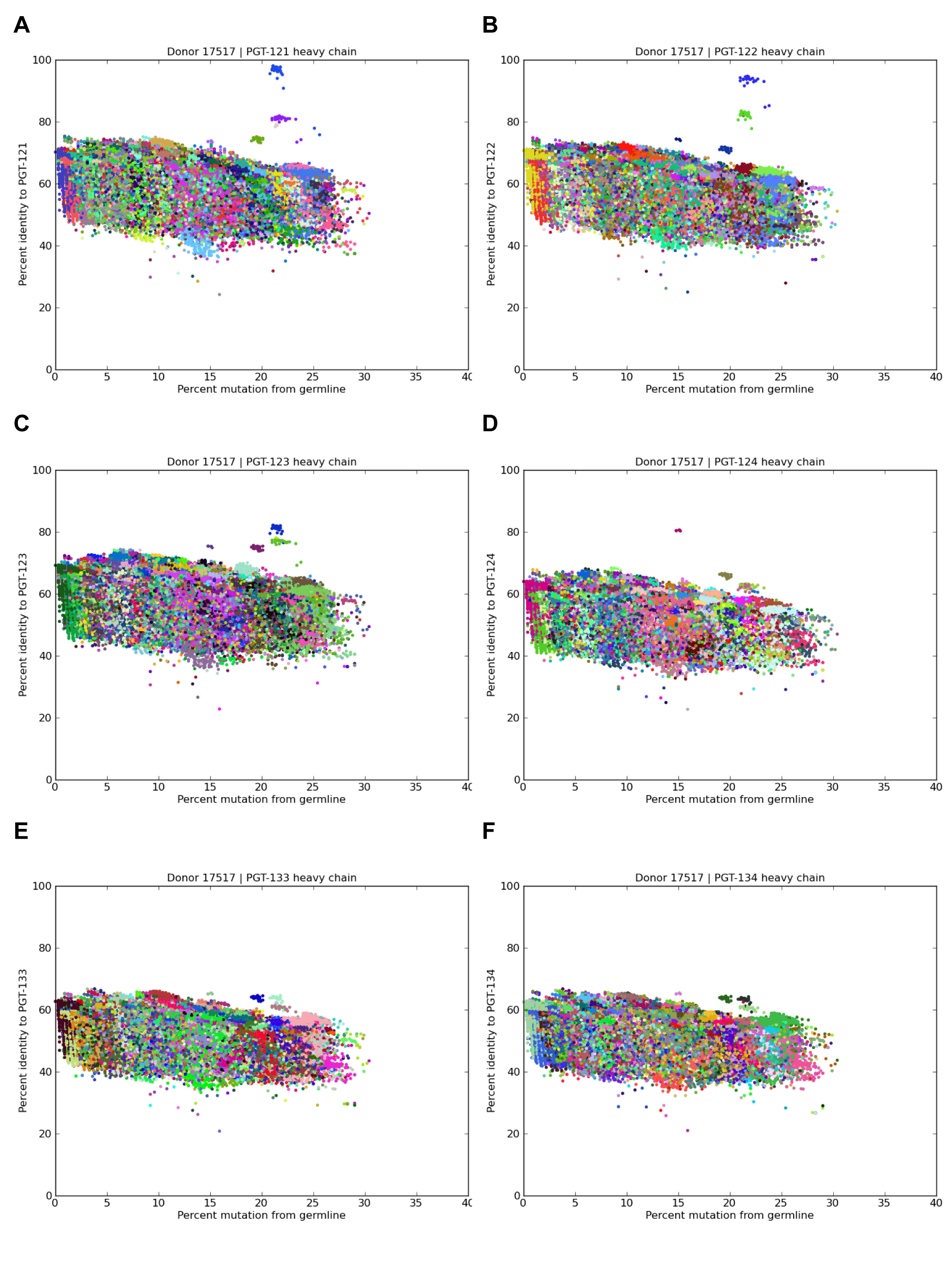

Supplement: Figure S1 — PGT121–134 heavy chain divergence plots. Data generated from 454 sequencing were graphed in identity (PGT bNAb) vs. divergence (germline) plots. Each color represents a unique clone, which was determined using the procedures outlined in the methods section. Divergence plots were created for (A) PGT121, (B) PGT122, (C) PGT123, (D) PGT124, (E) PGT133, and (F) PGT134. (TIF) [file ppat.1003754.s001.tif]

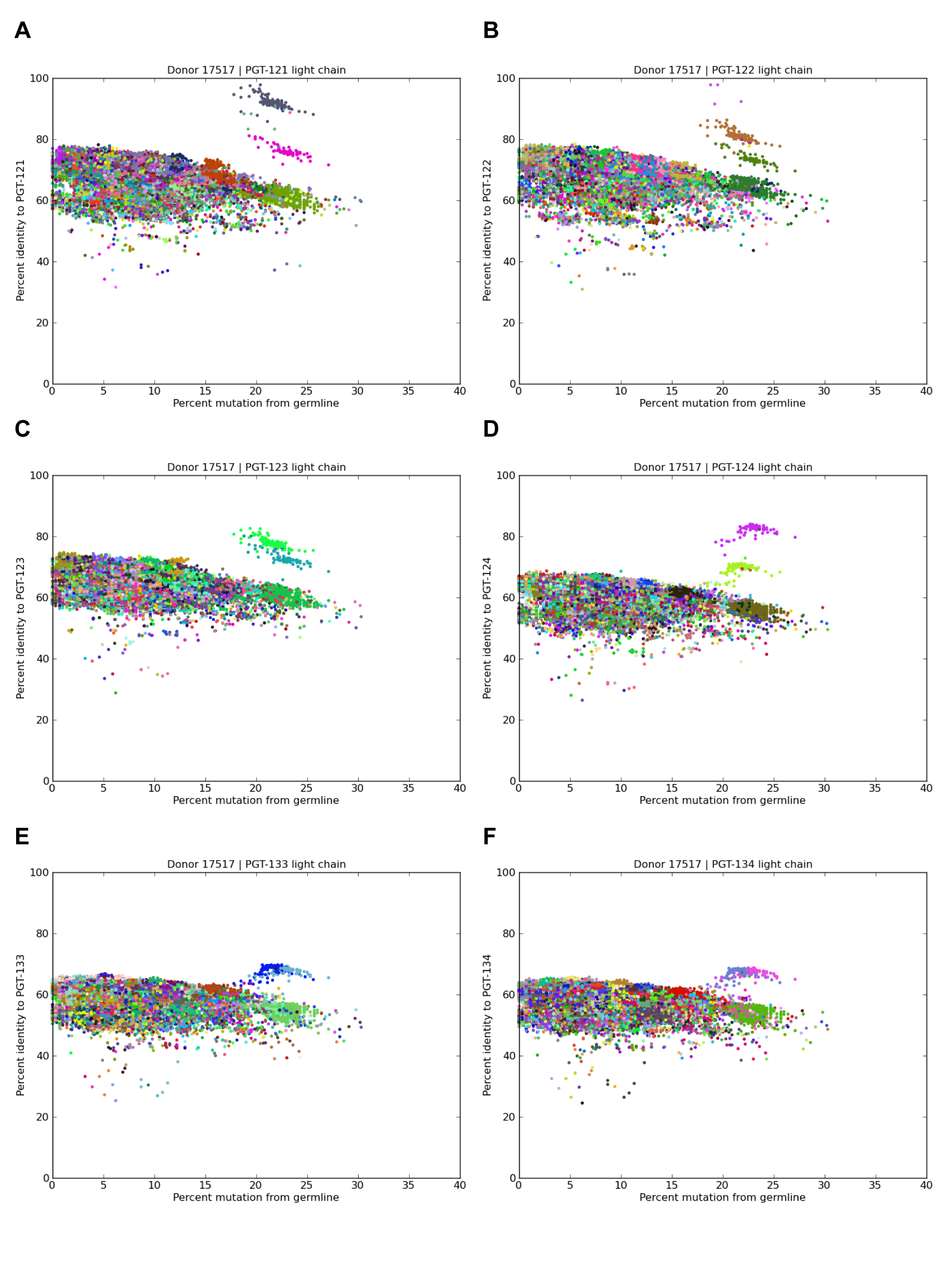

Supplement: Figure S2 — PGT121–134 light chain divergence plots. Data generated from 454 sequencing were graphed in identity (PGT bNAb) vs. divergence (germline) plots. Each color represents a unique clone, which was determined using the procedures outlined in the methods section. Divergence plots were created for (A) PGT121, (B) PGT122, (C) PGT123, (D) PGT124, (E) PGT133, and (F) PGT134. (TIF) [file ppat.1003754.s002.tif]

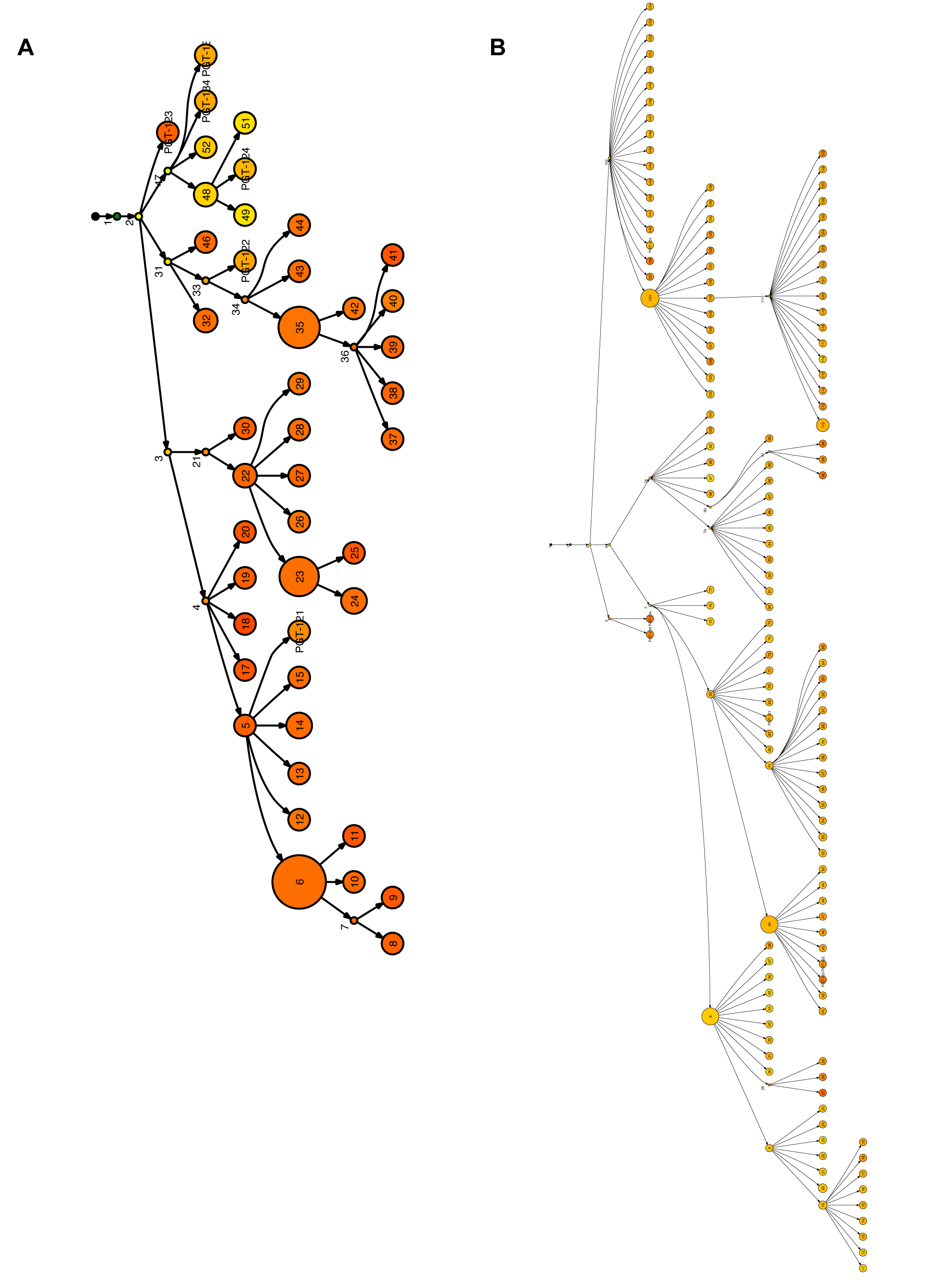

Supplement: Figure S3 — ImmuniTree without 454 sequencing error correction. (A) Heavy chain SHM phylogeny inferred by the ImmuniTree algorithm without correcting for 454 sequencing errors. (B) Light chain SHM phylogeny inferred by the ImmuniTree algorithm without correcting for 454 sequencing errors. Trees were generated using the same set of sequences to build the trees in Figure 2. (TIF) [file ppat.1003754.s003.tif]

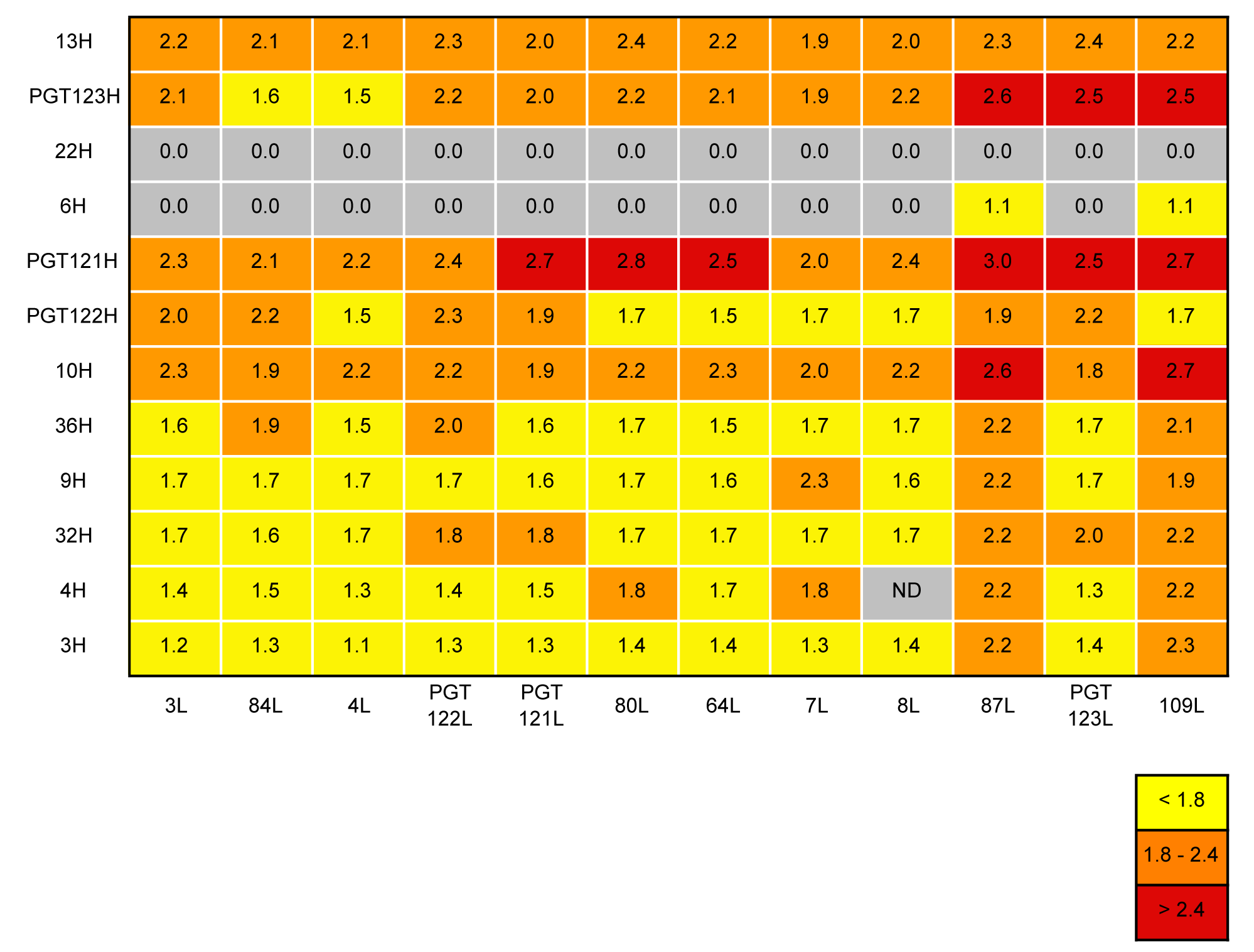

Supplement: Figure S4 — Neutralization score of each pair tested on 6-virus panel. Each antibody of different heavy and light chain pairs was produced in 293T cells and tested for neutralization activity on a cross-clade 6-virus panel. Neutralization score was calculated using the formula: mean(log10(10/IC50)). Boxes were colored as follows: score≤1.7, yellow; 1.8≤score ≤2.5, orange; score>2.5, red. (TIF) [file ppat.1003754.s004.tif]

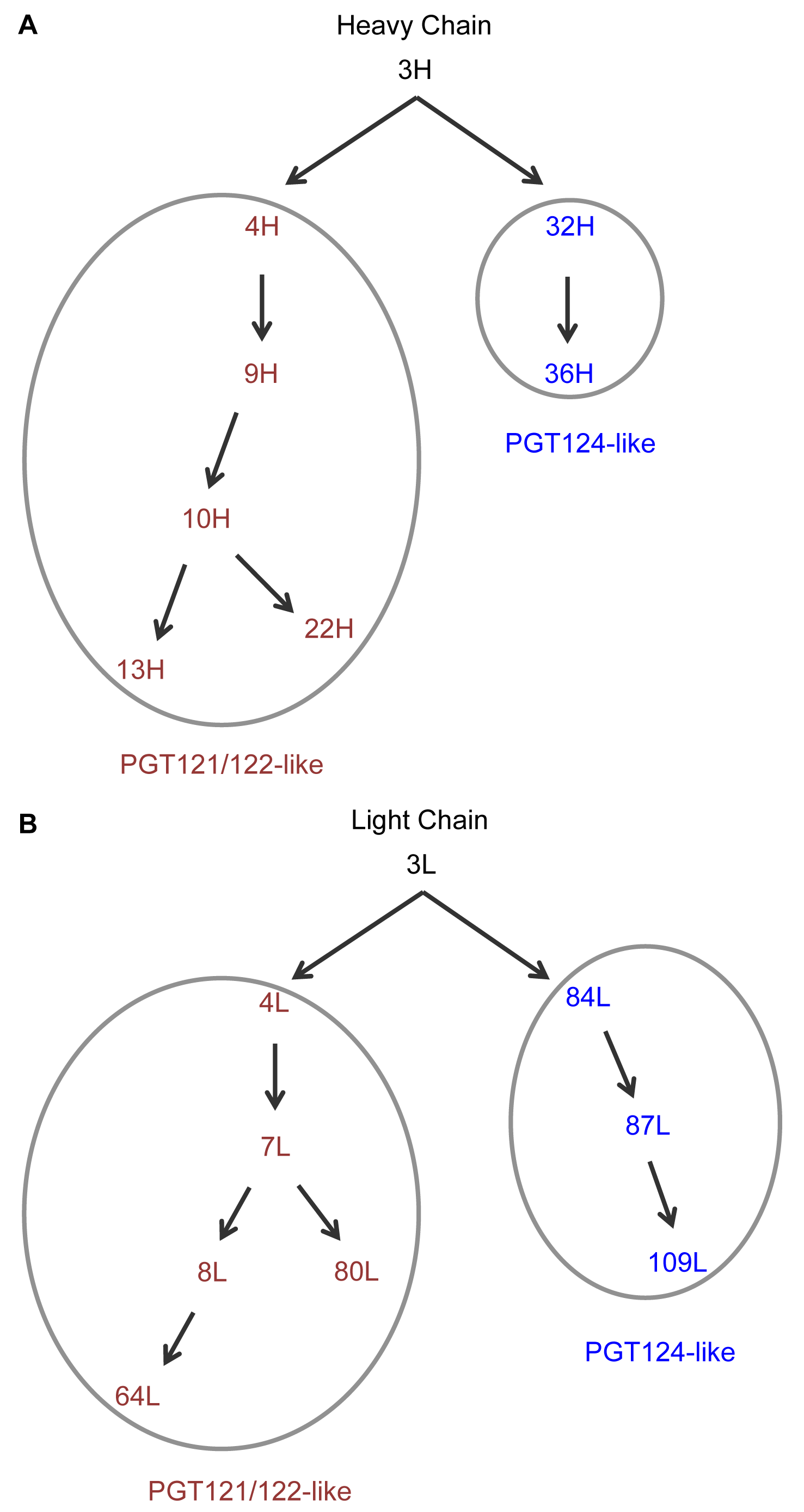

Supplement: Figure S5 — Nodes can be classified as more PGT121-like or more PGT124-like based on ImmuniTree clustering. (A) Heavy chain nodes selected for pairing and characterization are shown divided into PGT121-like or PGT124-like branches based on ImmuniTree. (B) Light chain nodes selected for pairing and characterization are shown divided into PGT121-like or PGT124-like branches based on ImmuniTree. (TIF) [file ppat.1003754.s005.tif]

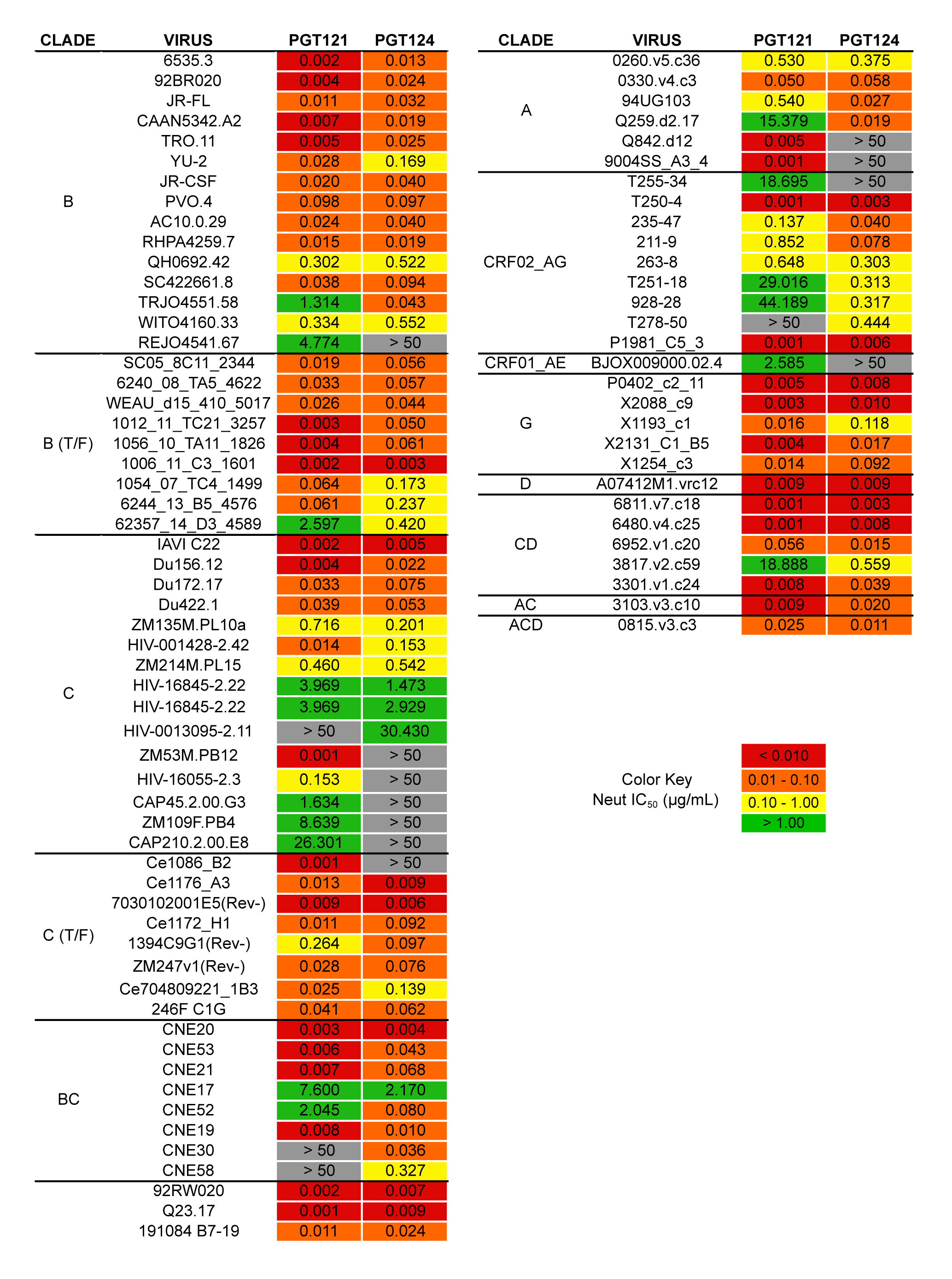

Supplement: Figure S6 — Comparison of neutralization profile between PGT121 and PGT124. PGT121 and PGT124 were tested on 87 cross-clade isolates to determine neutralization breadth and potency. Listed in colored boxes are IC50 values (µg/ml) of each isolate neutralized. (TIF) [file ppat.1003754.s006.tif]

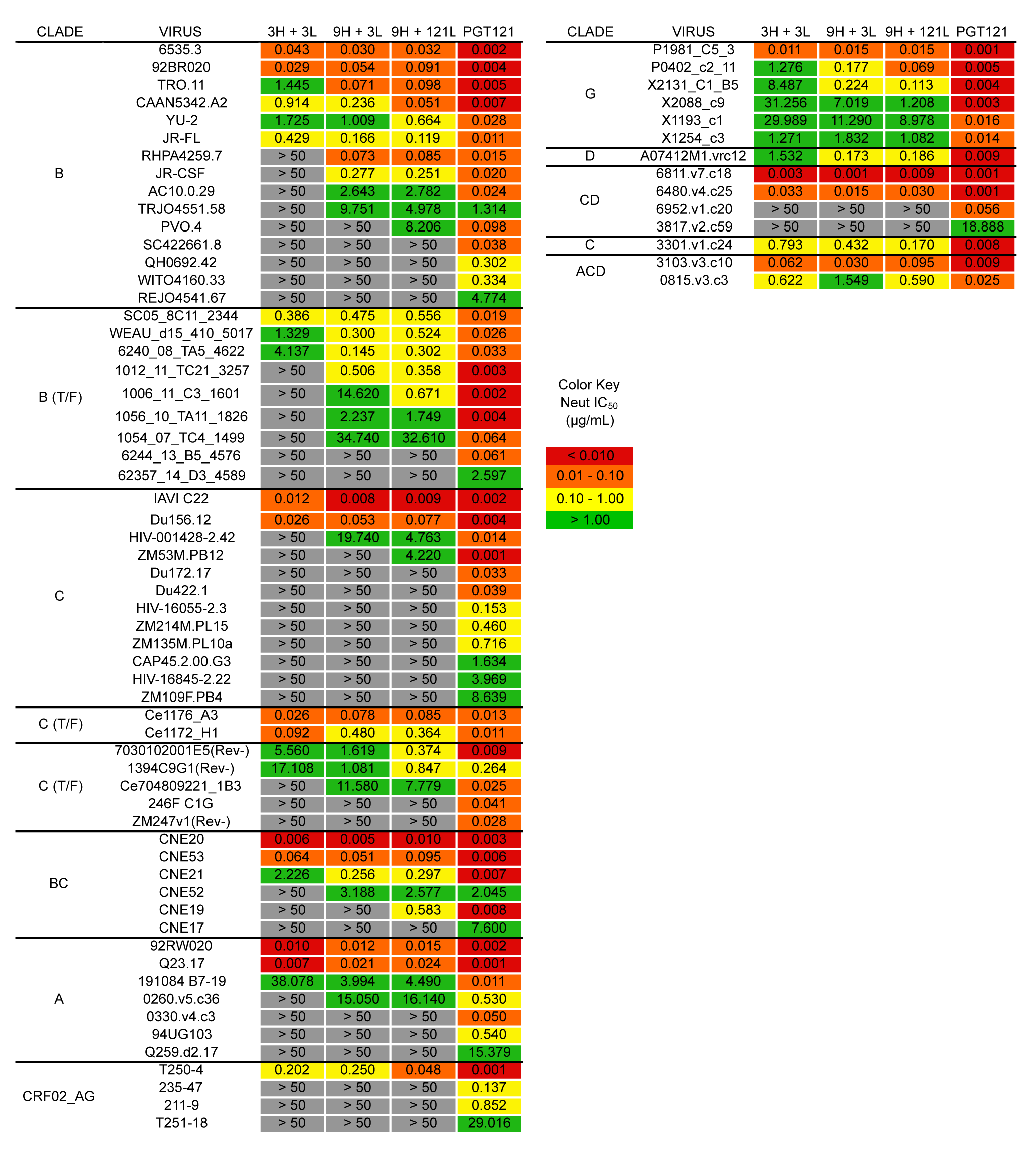

Supplement: Figure S7 — Inferred intermediates of PGT121 were evaluated for neutralization breadth and potency. Heavy and light chain nodes leading to mAb PGT121 were paired and tested on a 74-virus panel of PGT121-sensitive viruses. Listed in colored boxes are IC50 values (µg/ml) of each isolate neutralized. (TIF) [file ppat.1003754.s007.tif]

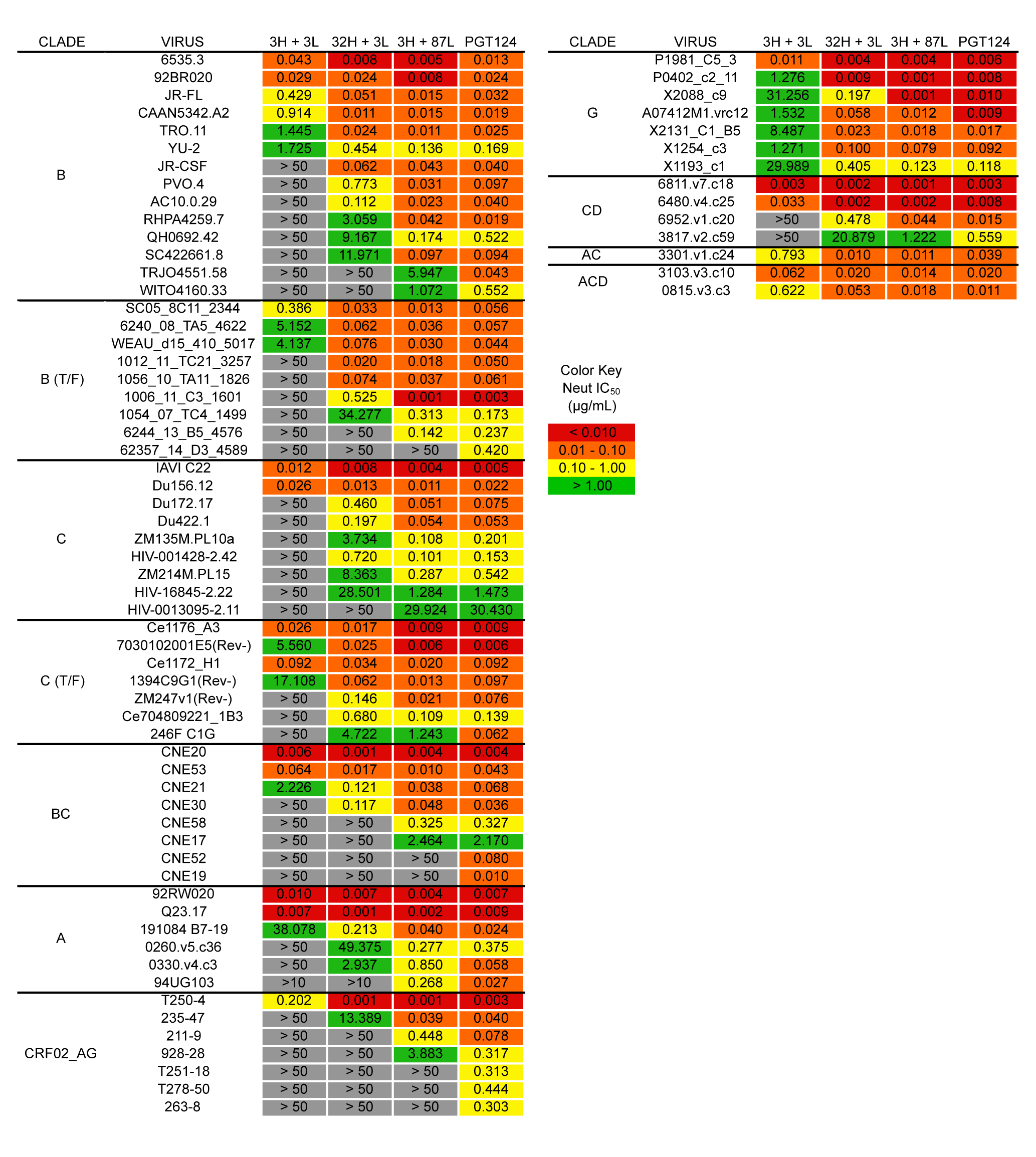

Supplement: Figure S8 — Inferred intermediates of PGT124 were evaluated for neutralization breadth and potency. Heavy and light chain nodes leading to mAb PGT124 were paired and tested on a 74-virus panel of PGT124-sensitive viruses. Listed in colored boxes are IC50 values (µg/ml) of each isolate neutralized. (TIF) [file ppat.1003754.s008.tif]

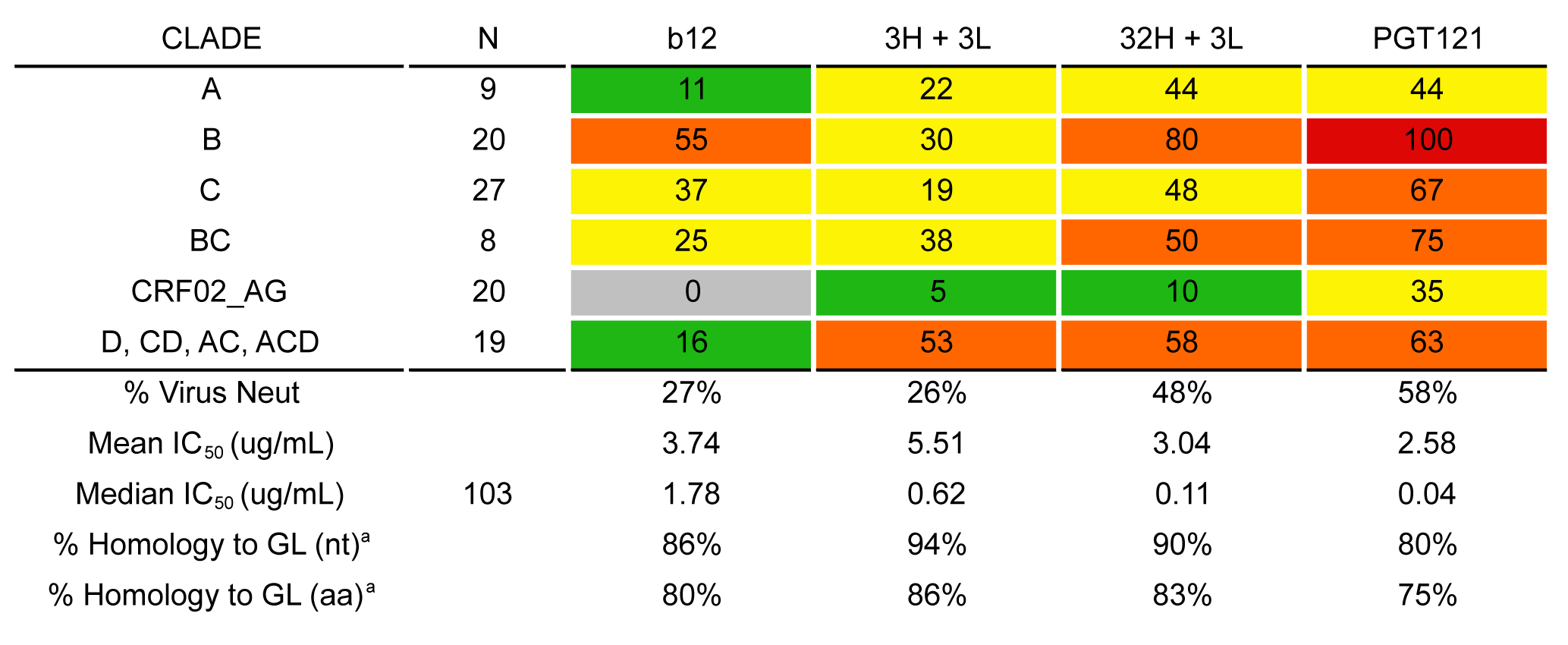

Supplement: Figure S9 — Neutralization table summary of putative intermediates in comparison to b12. Putative intermediates 3H+3L and 32H+3L were tested in comparision to PGT121 and b12 in TZM-bl neutralization assays on a cross-clade 103 pseudovirus panel. Listed in colored boxes are percentages of each clade neutralized. Boxes are colored as follows: percent of viruses neutralized <25% (green), percent of viruses neutralized: 25–50% (yellow), percent of viruses neutralized: 50–100 90% (red). aMutation frequency was calculated over the V-gene and J-gene as nucleotides (nt) or amino acids (aa) differing from the putative germline sequence. The CDR3 regions and insertions and deletions were excluded from the analysis. (TIF) [file ppat.1003754.s009.tif]

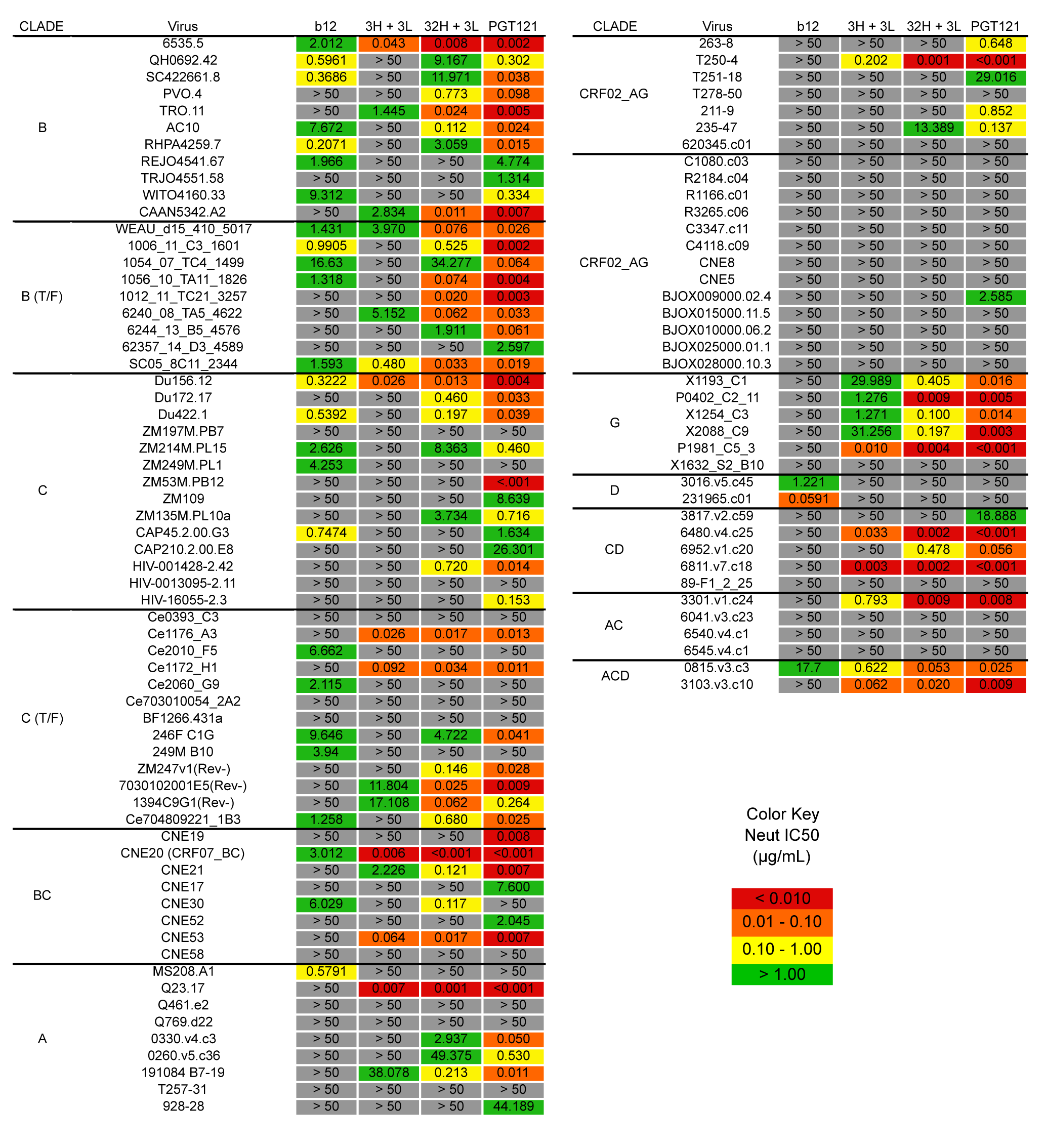

Supplement: Figure S10 — Neutralization panel of putative intermediates in comparison to b12. Inferred intermediates 3H+3L, and 32H+3L were tested in comparision to PGT121 and b12 in TZM-bl neutralization assays on a cross-clade 103 pseudovirus panel. Listed IC50 values are in ug/mL. (TIF) [file ppat.1003754.s010.tif]

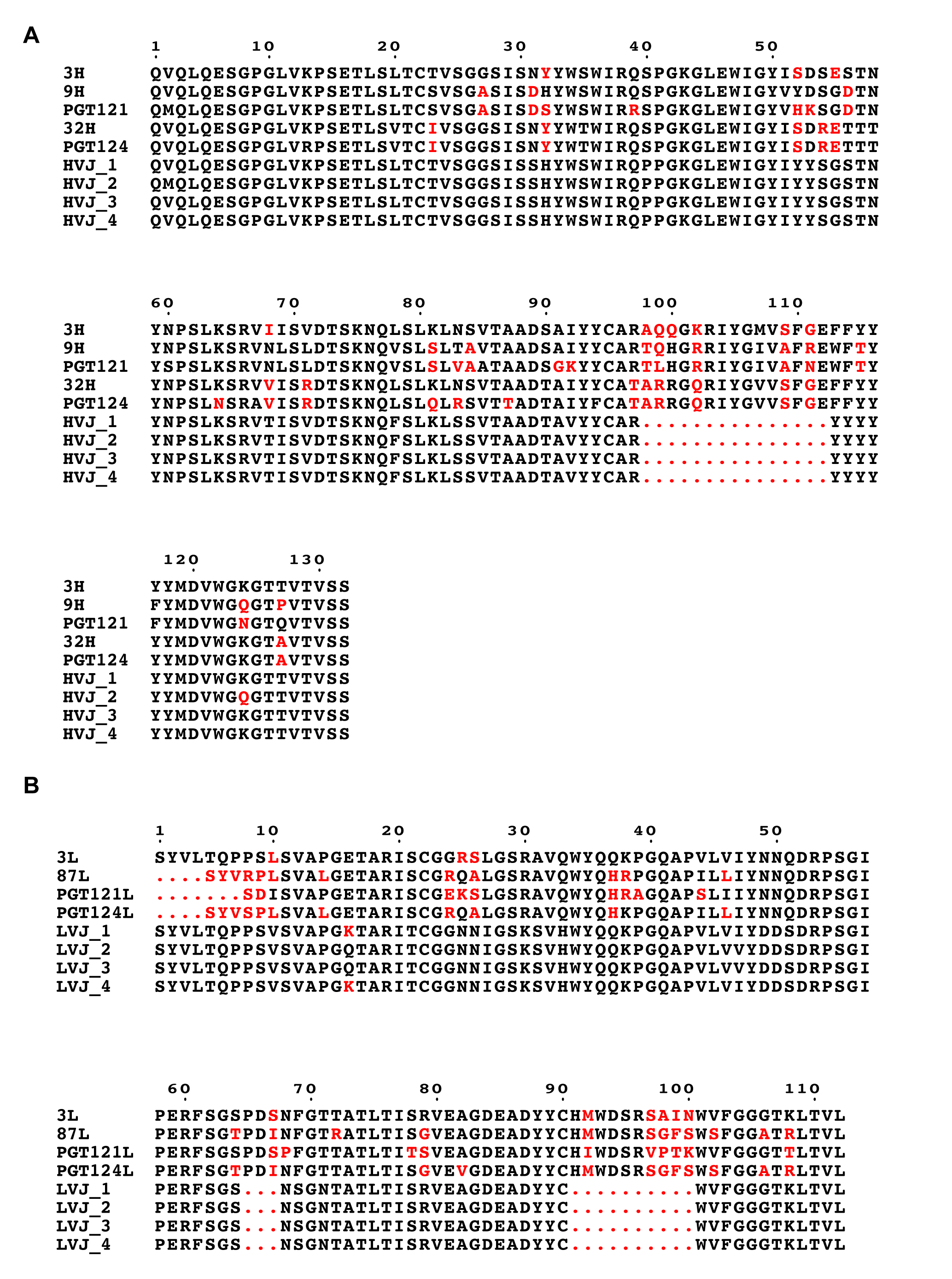

Supplement: Figure S11 — Alignment of intermediate and mature antibodies to germline. Genomic DNA from the PGT121–123 donor was extracted from CD4+ T cells, which were enriched through anti-CD4 antibody coated magnetic beads. (A) Primers designed to amplify the IGHV4-59 and the IGHJ6*03 gene families were used to generate heavy chain libraries. (B) Primers designed to amplify the IGLV3-21 and IGLJ3*02 gene families were used to produce light chain libraries. Both libraries were then TOPO cloned into vectors and individual colonies were subsequently Sanger sequenced to determine germline sequences. Alignments were made using ClustalW. (TIF) [file ppat.1003754.s011.tif]

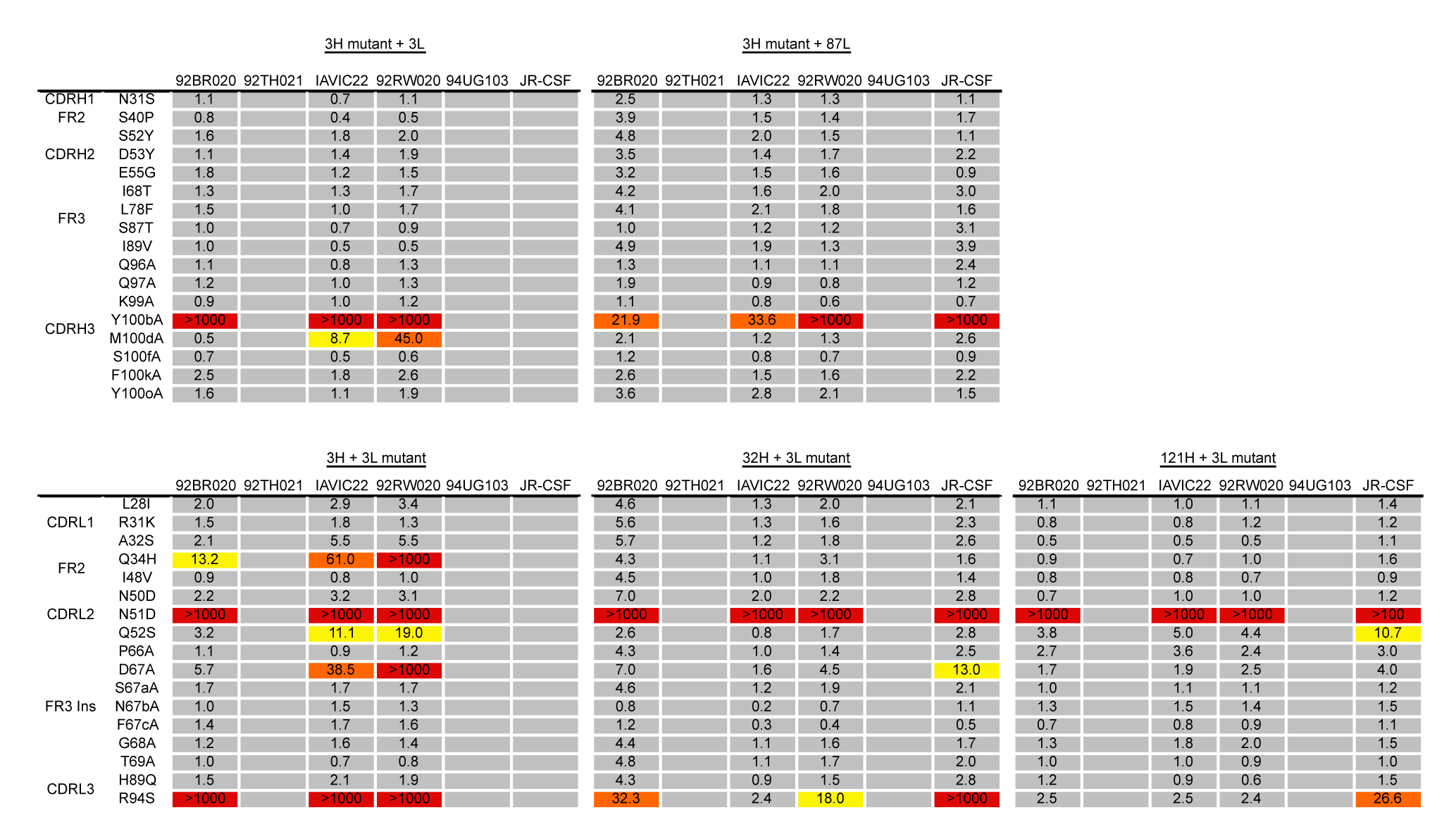

Supplement: Figure S12 — Paratope mapping of 3H and 3L paired with heavy and light chains of different maturation levels. Alanine scanning mutagenesis of heavy chain 3H paired with less-mutated light chain 3L and highly-mutated light chain 87L as well as alanine scanning mutagenesis of 3L paired with less-mutated 3H, moderately-mutated 32H, and highly-mutated PGT121H. Values represent fold-changes in IC50 using formula: Mutant(IC50)/WT(IC50). Empty gray boxes represent isolates for which the antibody did not neutralize (TIF) [file ppat.1003754.s012.tif]

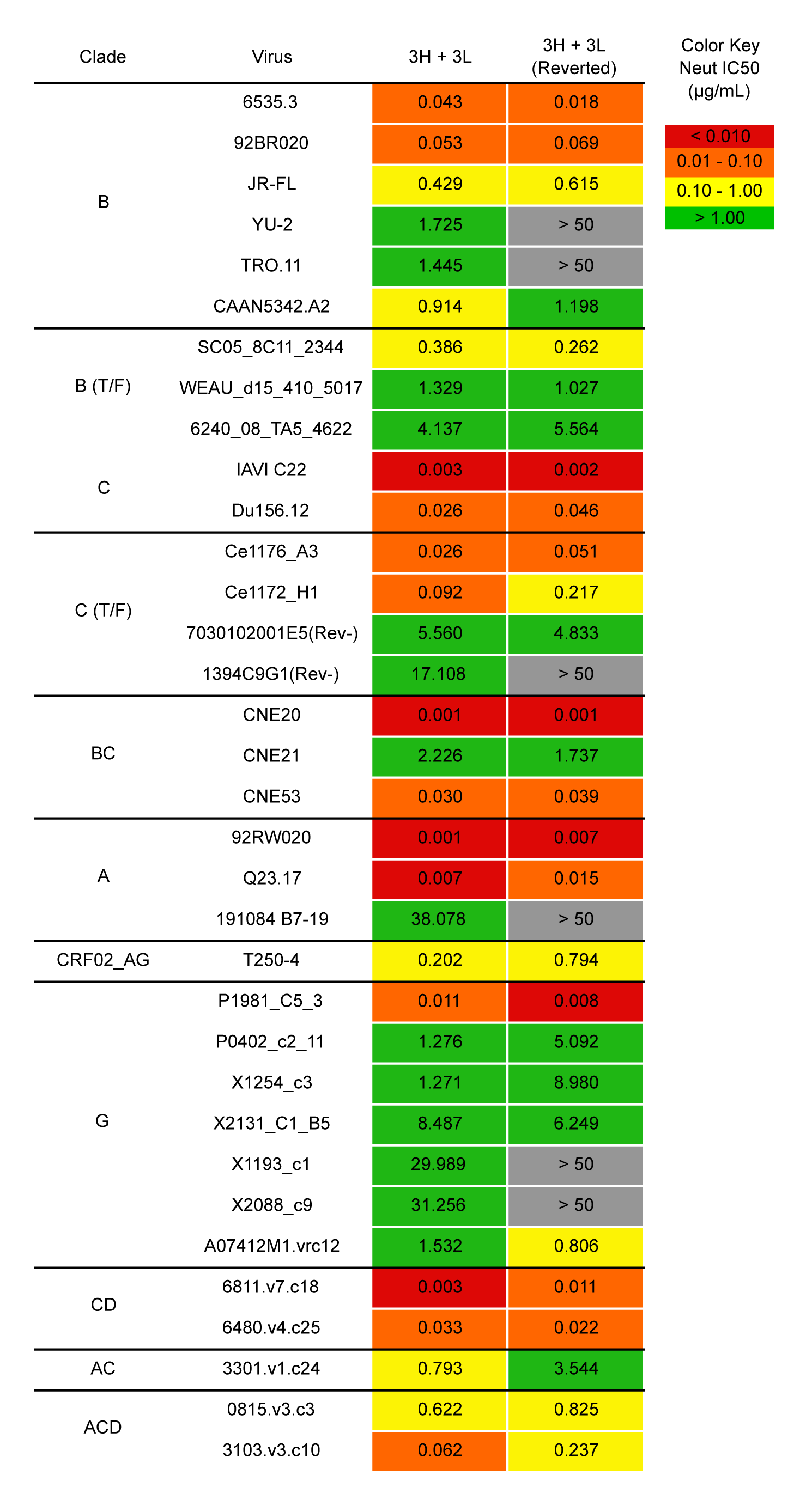

Supplement: Figure S13 — Reversion of all residues in 3H except CDRH3 results in loss of neutralization for 20% of isolates. Alanine scanning mutagenesis of heavy chain 3H paired with 3L were tested in TZM-bl neutralization assays on viruses previously determined to be neutralized by 3H+3L. Listed IC50 values are in ug/mL. (TIF) [file ppat.1003754.s013.tif]

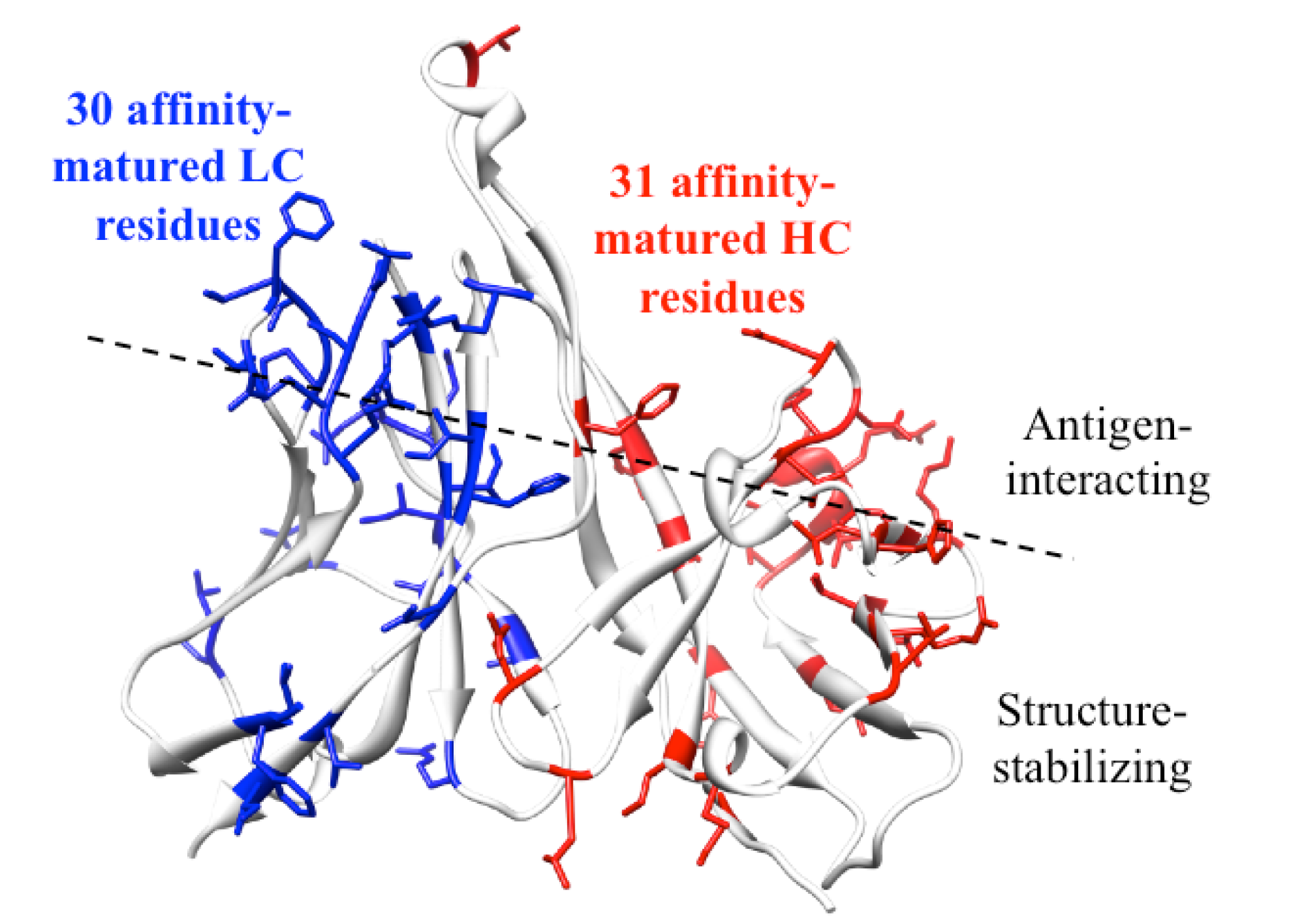

Supplement: Figure S14 — Many of the residues from SHM may play a role in stabilizing antibody structure and conformation. Structural representation of the mutations that occurred in PGT123 during affinity maturation from a putative germline antibody. Residues thought to have mutated are shown in blue and red for the light and heavy chains, respectively. Mutations are seen to have taken place across all regions of the Fv domain and contribute to both paratope evolution and antibody architecture. (TIF) [file ppat.1003754.s014.tif]

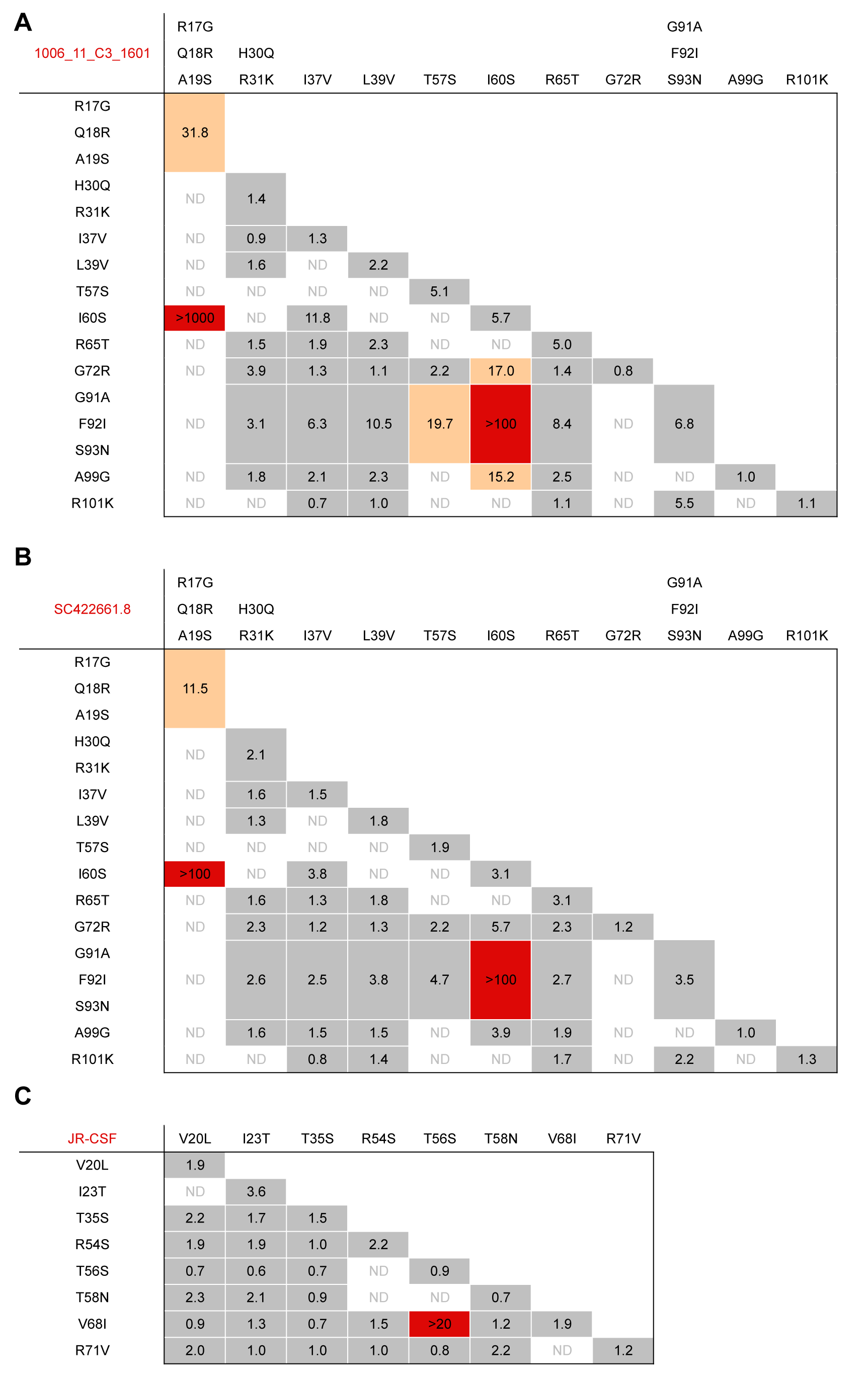

Supplement: Figure S15 — Single and double mutant summary of inferred intermediate antibodies 3H+87L to 3H+3L and 32H+3L to 3H+3L. Reversion of single and double residues of light chain 87L to light chain 3L and paired with heavy chain 3H. WT and reverted mutants were tested on isolate (A) 1006_11_C3_1601 and (B) SC422661.8. (C) Reversion of single and double residues of heavy chain 32H to heavy chain 3H and paired with light chain 3L. WT and reverted mutants were tested on isolate JR-CSF. Values represent fold-changes in IC50 using formula Mutant(IC50)/WT(IC50). ND represent double mutants that were not determined. (TIF) [file ppat.1003754.s015.tif]

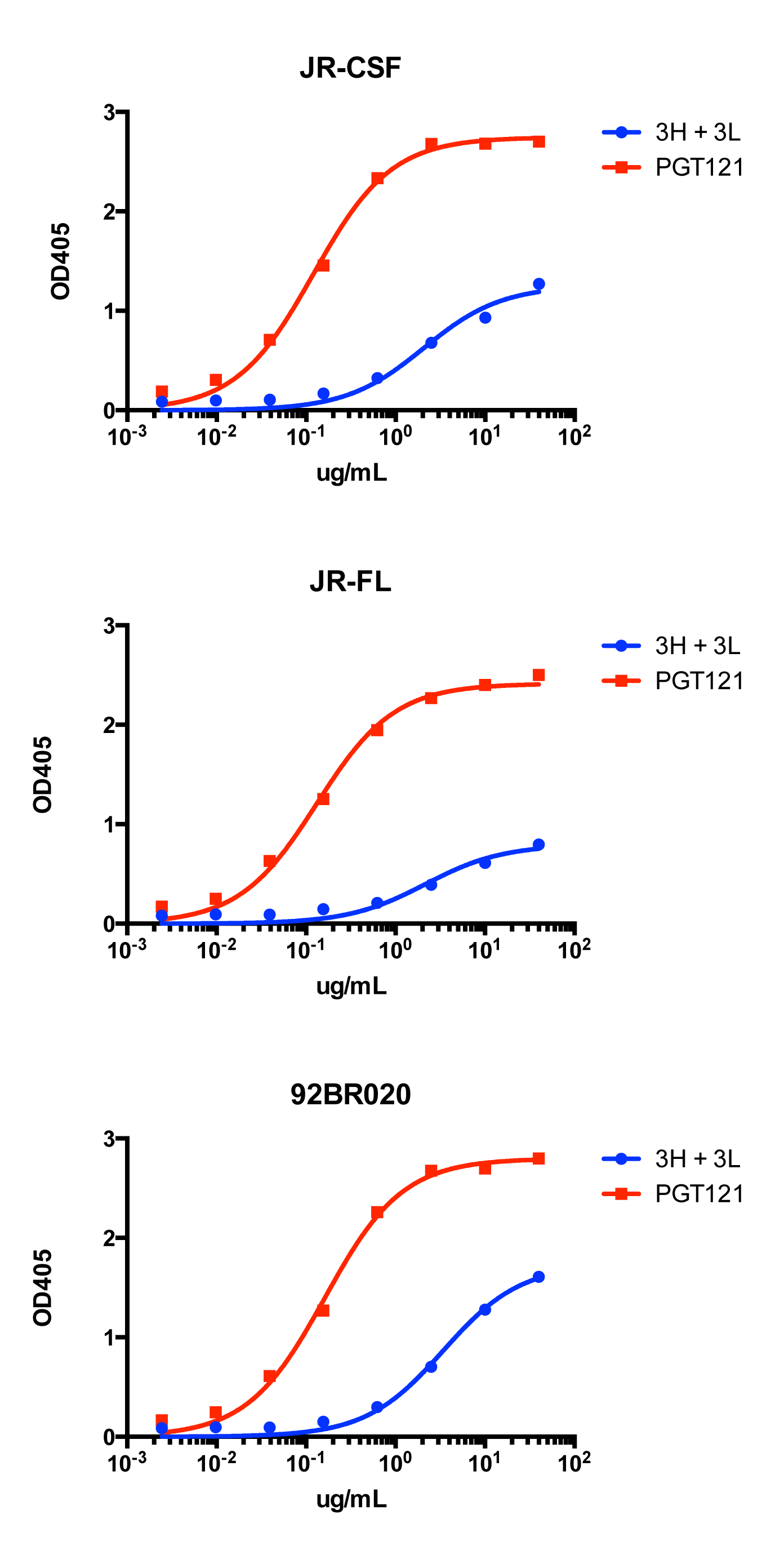

Supplement: Figure S16 — The least mutated antibody variant 3H+3L binds recombinant gp120 less well than the affinity mature PGT121. Recombinant gp120s were produced in 293F cells and purified by lectin column before use in ELISA binding assays. ELISA values are reported in optical density at 405 nm (OD405). (TIF) [file ppat.1003754.s016.tif]

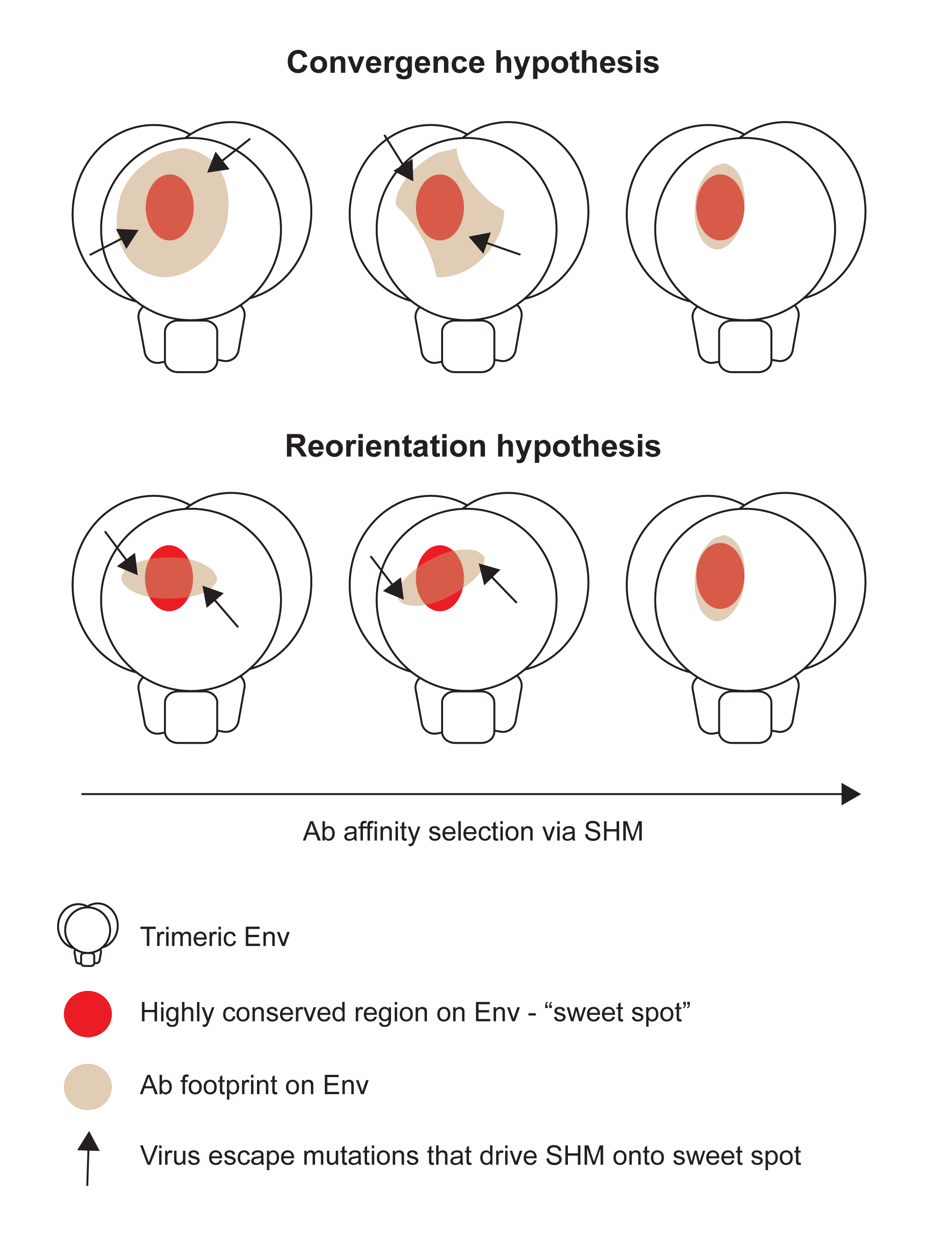

Supplement: Figure S17 — Illustration of role of somatic hypermutation in the development of neutralization breadth. Models for development of neutralization breadth via SHM by targeting the “sweet spot” on HIV Env. Breadth can either develop by converging onto or by reorienting onto a highly conserved region on HIV Env as illustrated. This convergence or reorientation is directed by virus escape mutations, which select for antibodies that interact with conserved regions (glycans, conserved side chains and main chain atoms) and less dependency on variable residues (variable side chains). By this process, affinity selection can lead to greater neutralization breadth and potency. (TIF) [file ppat.1003754.s017.tif]

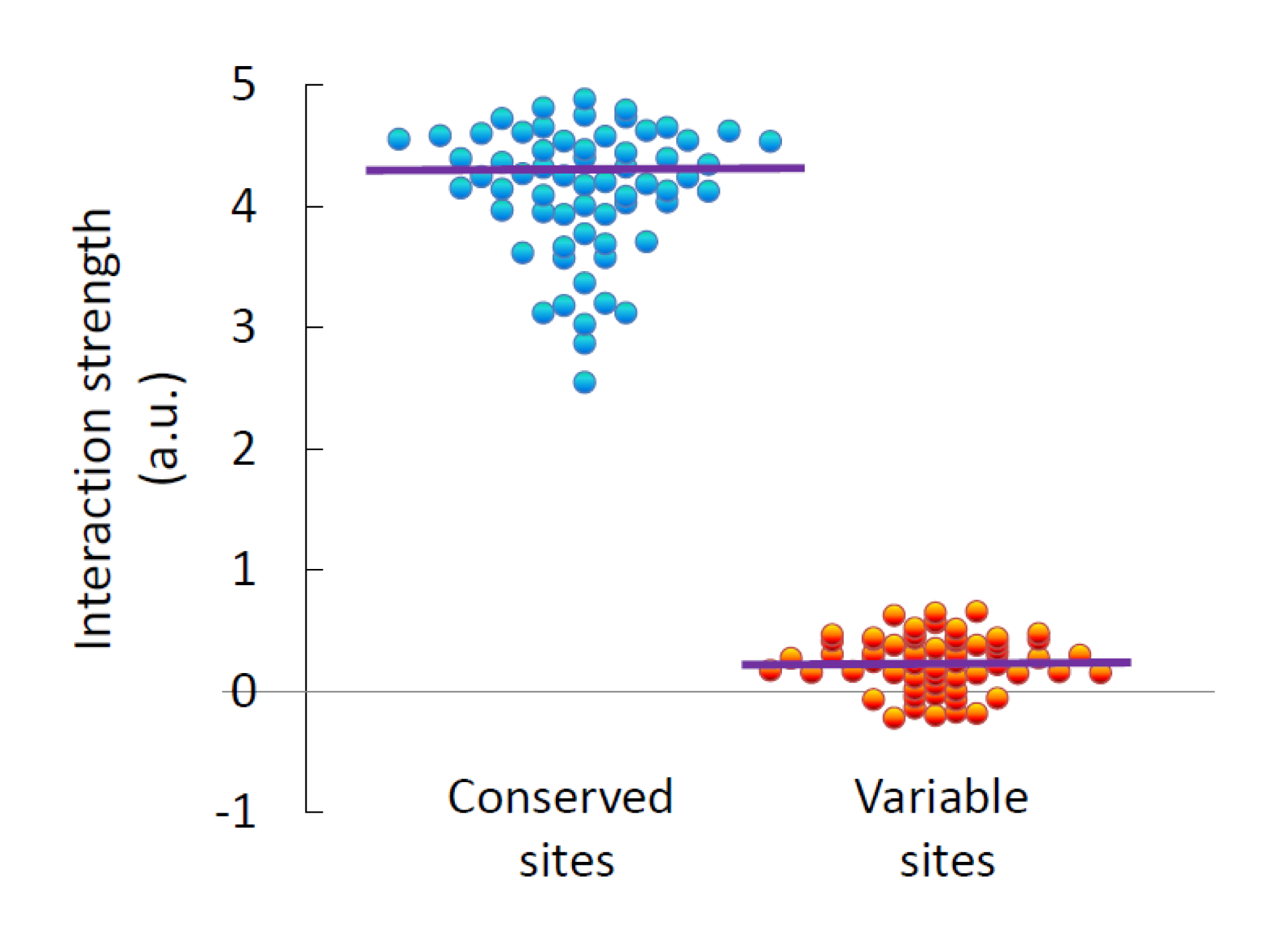

Supplement: Figure S18 — In silico experiments show that bNAbs form stronger contacts with conserved sites than with variable sites on Env. Each bNAb is represented by a pair of points, one red point and one blue point. The red point represents the average interaction strength of residues on the bNAb with variable sites of the antigen, and the blue point represents the average interaction strength with conserved antigen sites. The 63 red and blue points shown correspond to the 63 bNAbs that emerged in silico. The purple bars indicate the mean value averaged over all the selected antibodies. A significant difference between the mean values (purple bars) is apparent (P<0.001). (TIF) [file ppat.1003754.s018.tif]

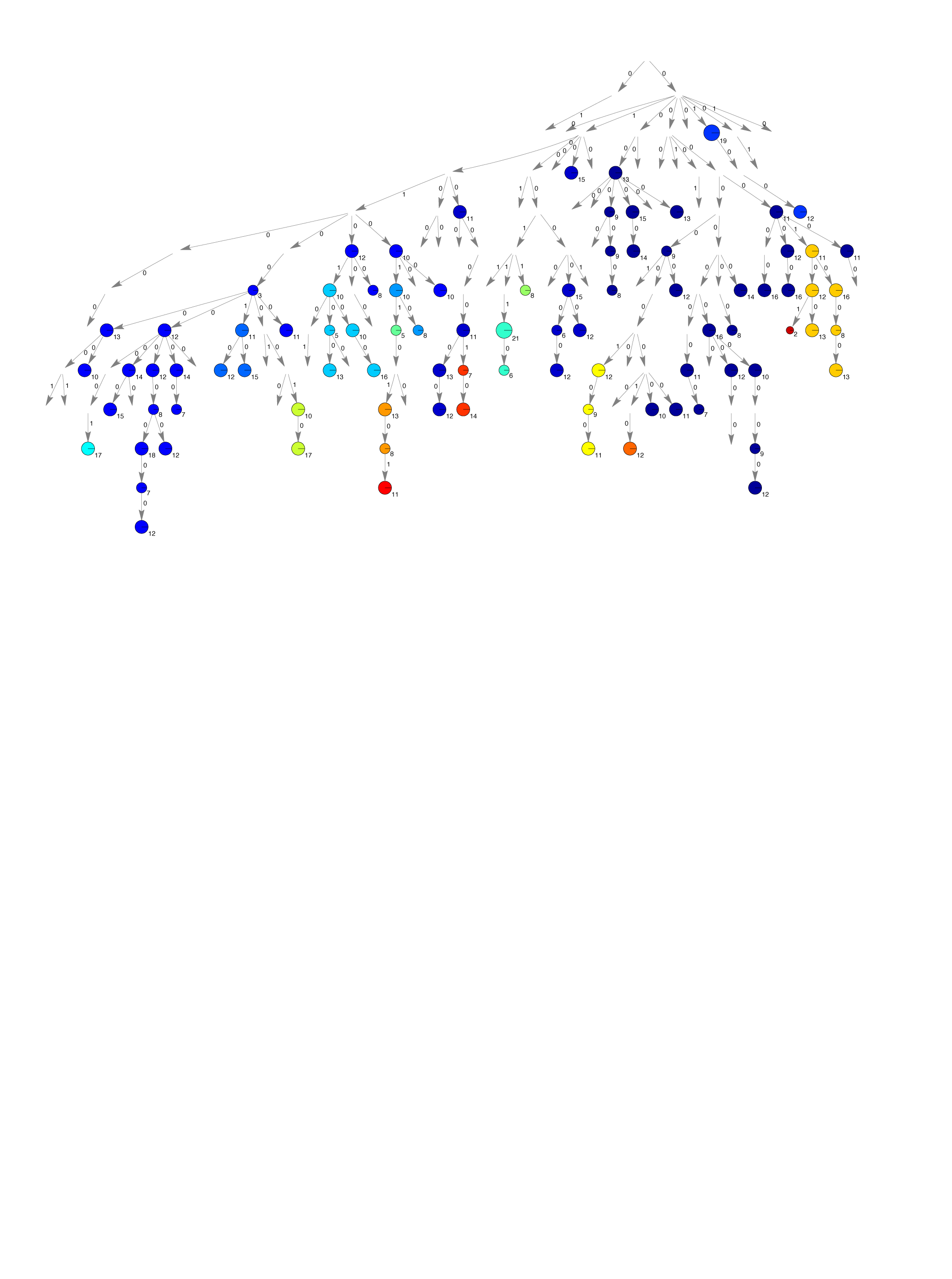

Supplement: Figure S19 — A draw from the generative model of a synthetic clone producing 1000 reads. Each node is a cell. The size of a cell is proportional to the number of reads it produced (with noise). which is also listed next to it. Each edge is a birth event and next to it is listed the mutation distance between parent and child. Cells with identical sequences are marked with same color. (TIF) [file ppat.1003754.s019.tif]

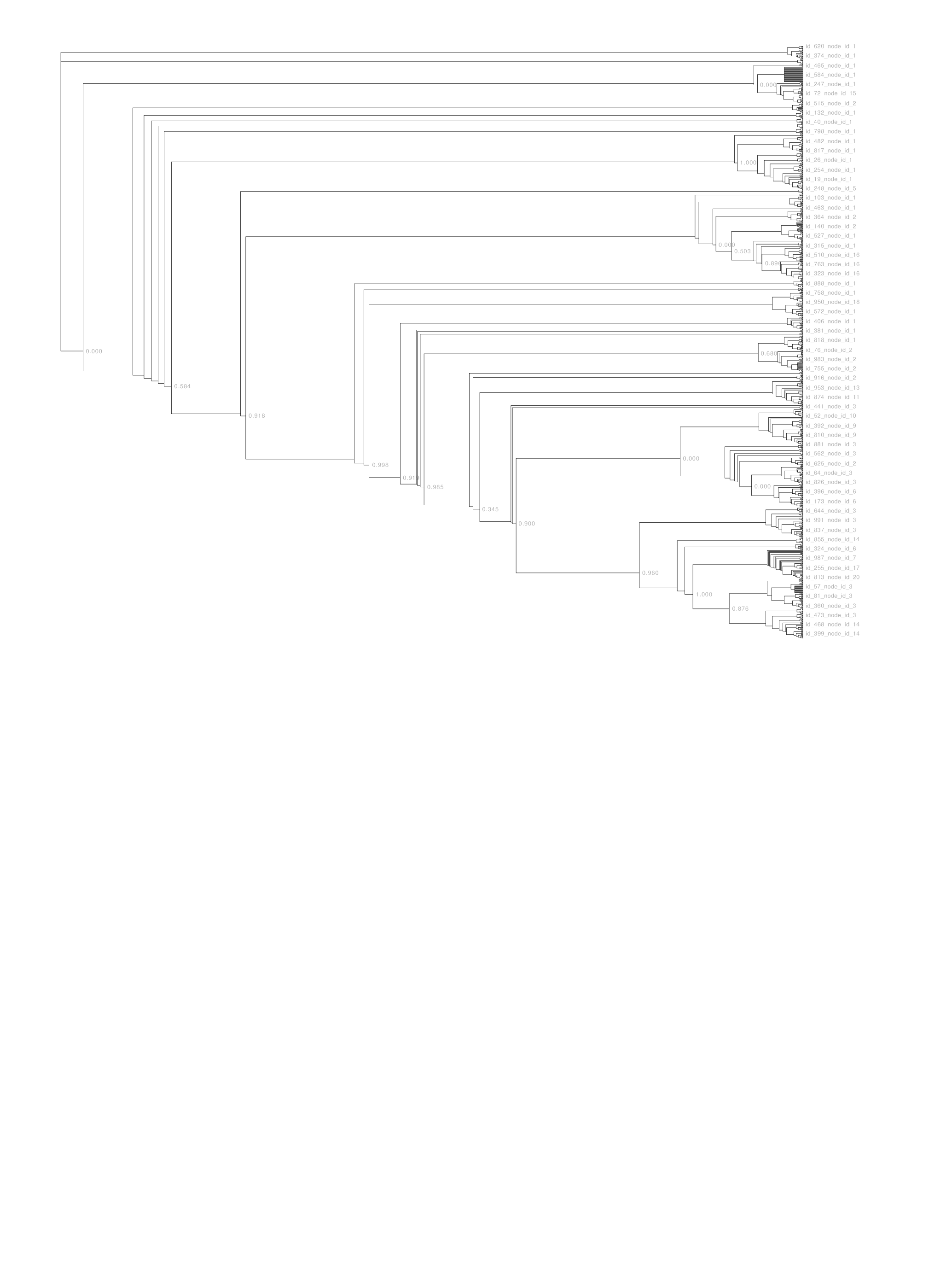

Supplement: Figure S20 — A binary tree by FastTree 2.0. A binary tree was generated using 1000 synthetic reads of Figure S19 and visualized using Archeopteryx. (TIF) [file ppat.1003754.s020.tif]

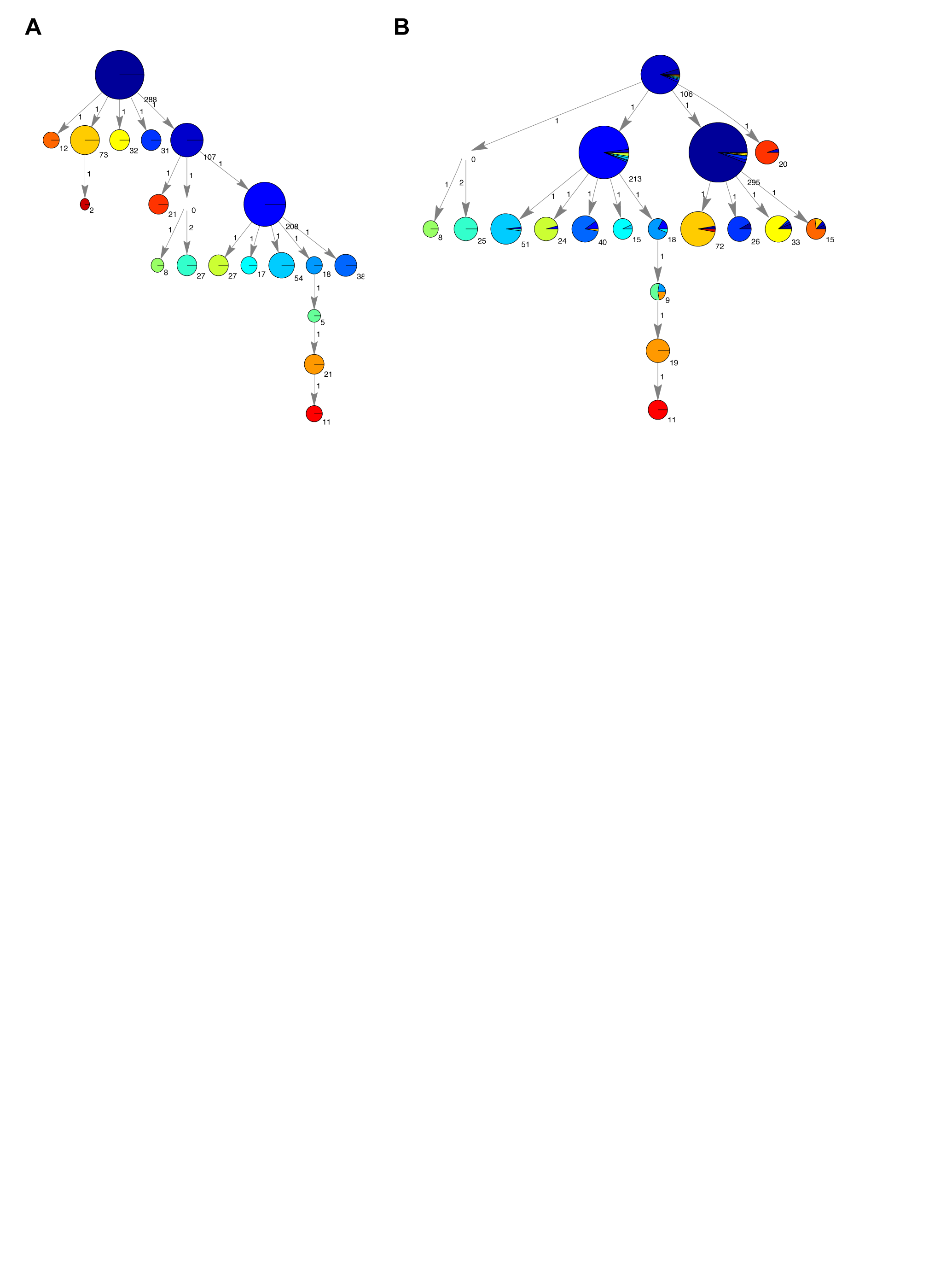

Supplement: Figure S21 — Mutation trees for the synthetic clone are obtained by collapsing edges marked with 0. (A) The true mutation tree for Figure S1. For tracking purposes, we color the reads of each cell by the cell's color. (B) The mutation tree returned by running ImmuniTree on the 1000 synthetic reads. Each cell is a pie chart showing the color composition of its reads. Almost each node in the reconstructed tree retained its identity, and the trees have an almost identical topology. (TIF) [file ppat.1003754.s021.tif]

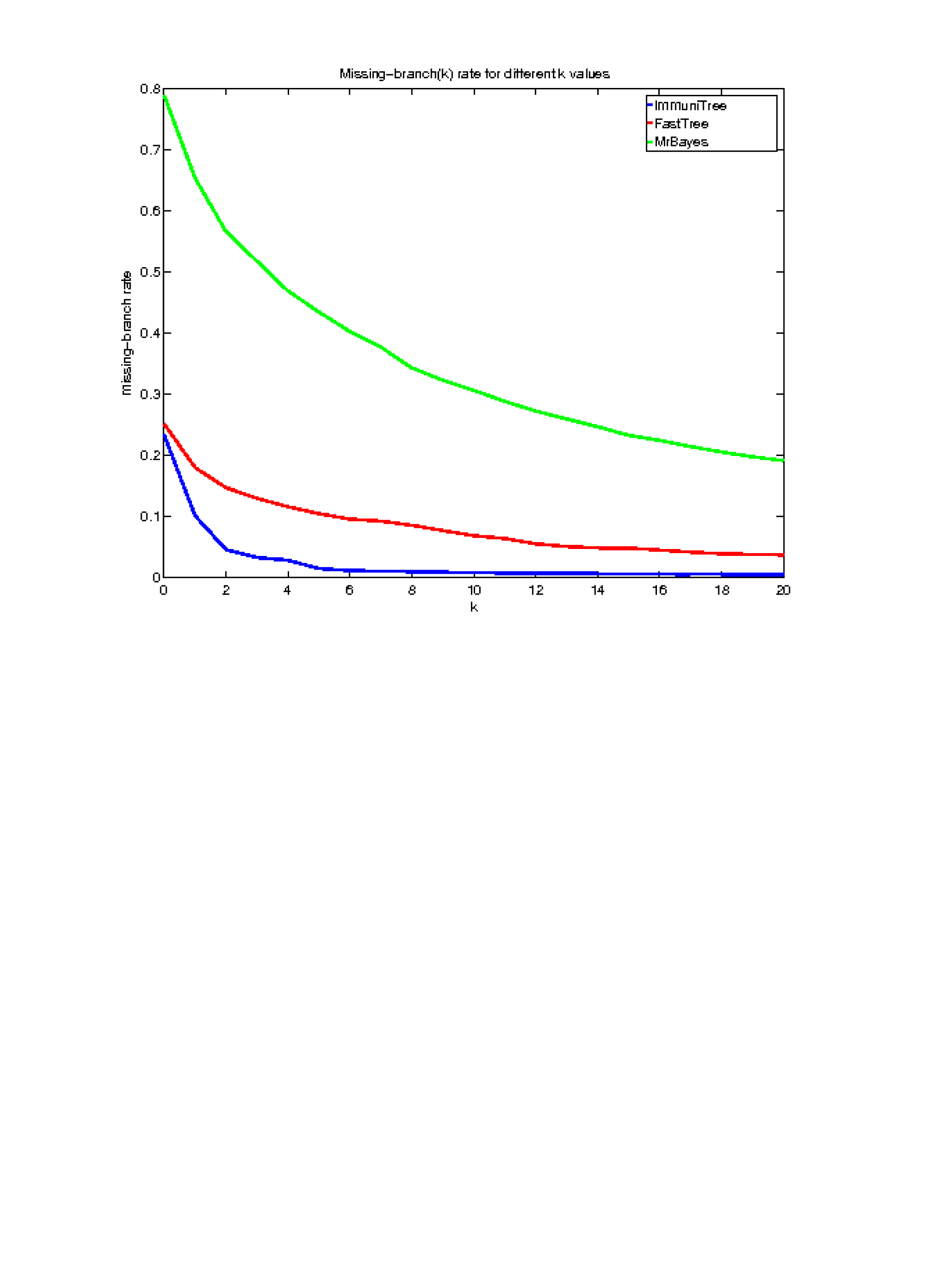

Supplement: Figure S22 — Comparison of the average missing-branch ( k ) rate for different values of k between FastTree and ImmuniTree over 100 synthetic clones. Comparison of the average missing-branch(k) rate, for different values of k, between FastTree and ImmuniTree, over 100 synthetic clones. From each synthetic clone we simulated 1000 reads, and let ImmuniTree and two other tree construction methods (FastTree and MrBayes) reconstruct the true tree that generated those reads. Each tree branch induces a partition of the reads into two sets (based on which side of the branch they are at). For each branch of the true tree we found the branch in the reconstructed tree with the minimum number of reads that need to switch sides in order for the induced partitions to match. Missing-branch(k) is the fraction of branches in the true tree for which the above minimum was greater than k. (TIF) [file ppat.1003754.s022.tif]

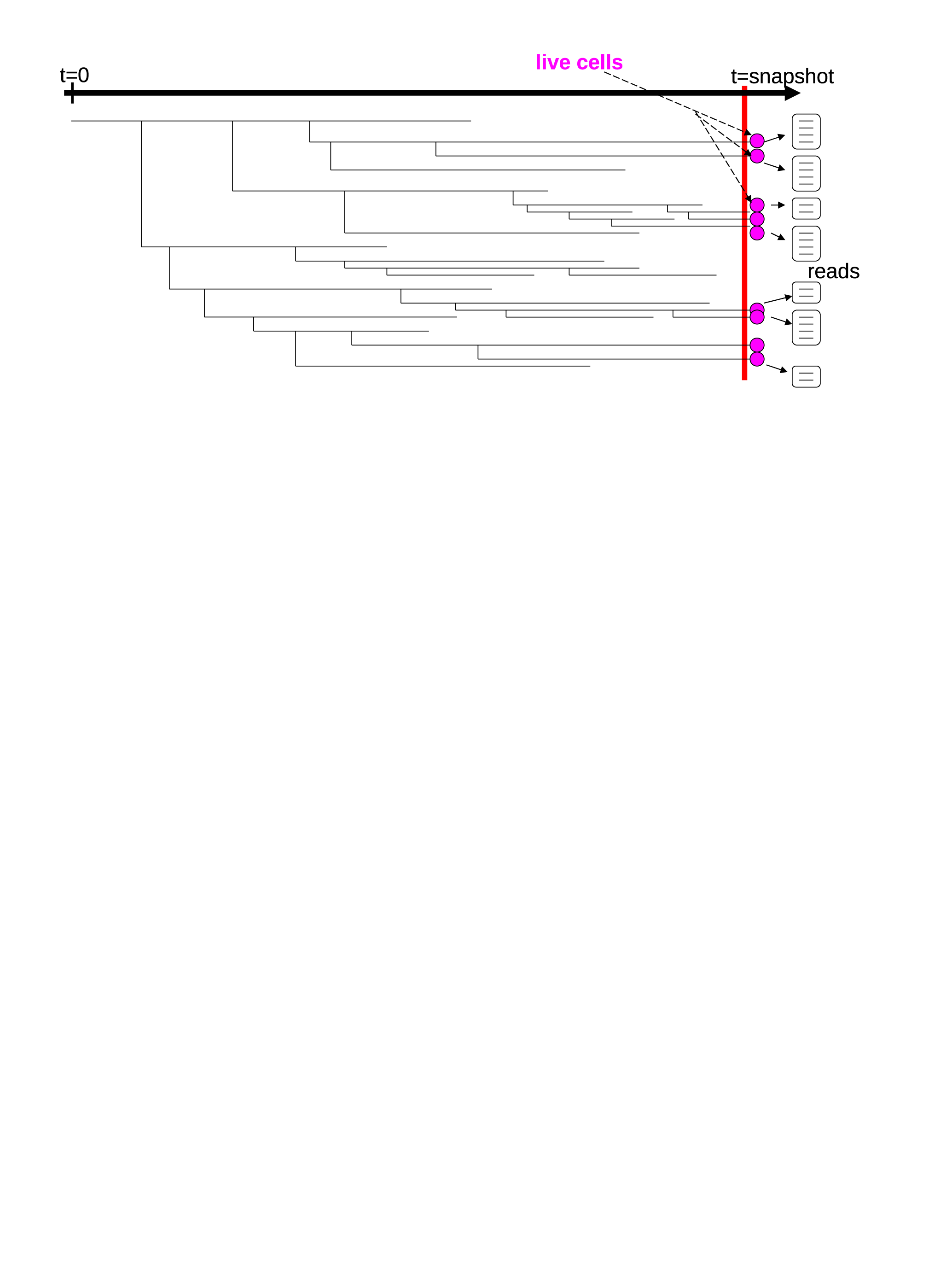

Supplement: Figure S23 — An illustration of the generative model. First, a tree of cells is generated, each cell has a parent cell, a birth time and a death time. Then, the sequences of these cells are filled in from top to bottom, each sequence is a copy of its parent, with a slight chance of mutation. Last, reads are generated from the cells alive at the end of the process, with added sequencing noise. (TIF) [file ppat.1003754.s023.tif]

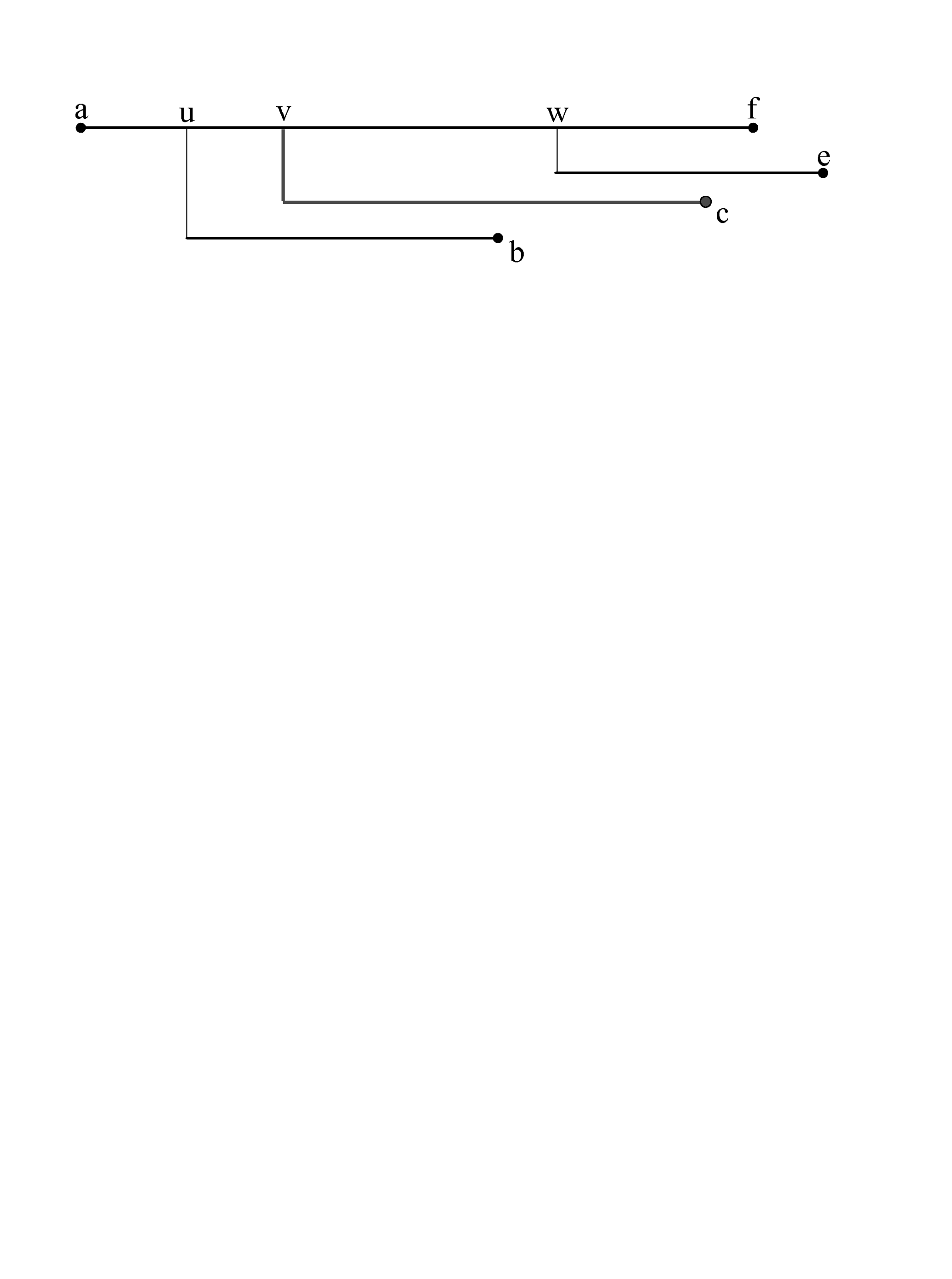

Supplement: Figure S24 — A subgraph of an internal node. Each horizontal line is a cell. Let the top cell be called R. v is a birth event in R's life, and nodes u and w are the previous and next events (we assume here they are both birth events). The nodes f, e, b, c represent the next event in each cell's life, either birth or death. Similarly, the node a represents the event preceding u on R's life, which could be either another birth or R's own birth. One of our MCMC moves proposes to disconnect the edge v→c from the tree, and reconnect it to a new position on this subgraph. (TIF) [file ppat.1003754.s024.tif]

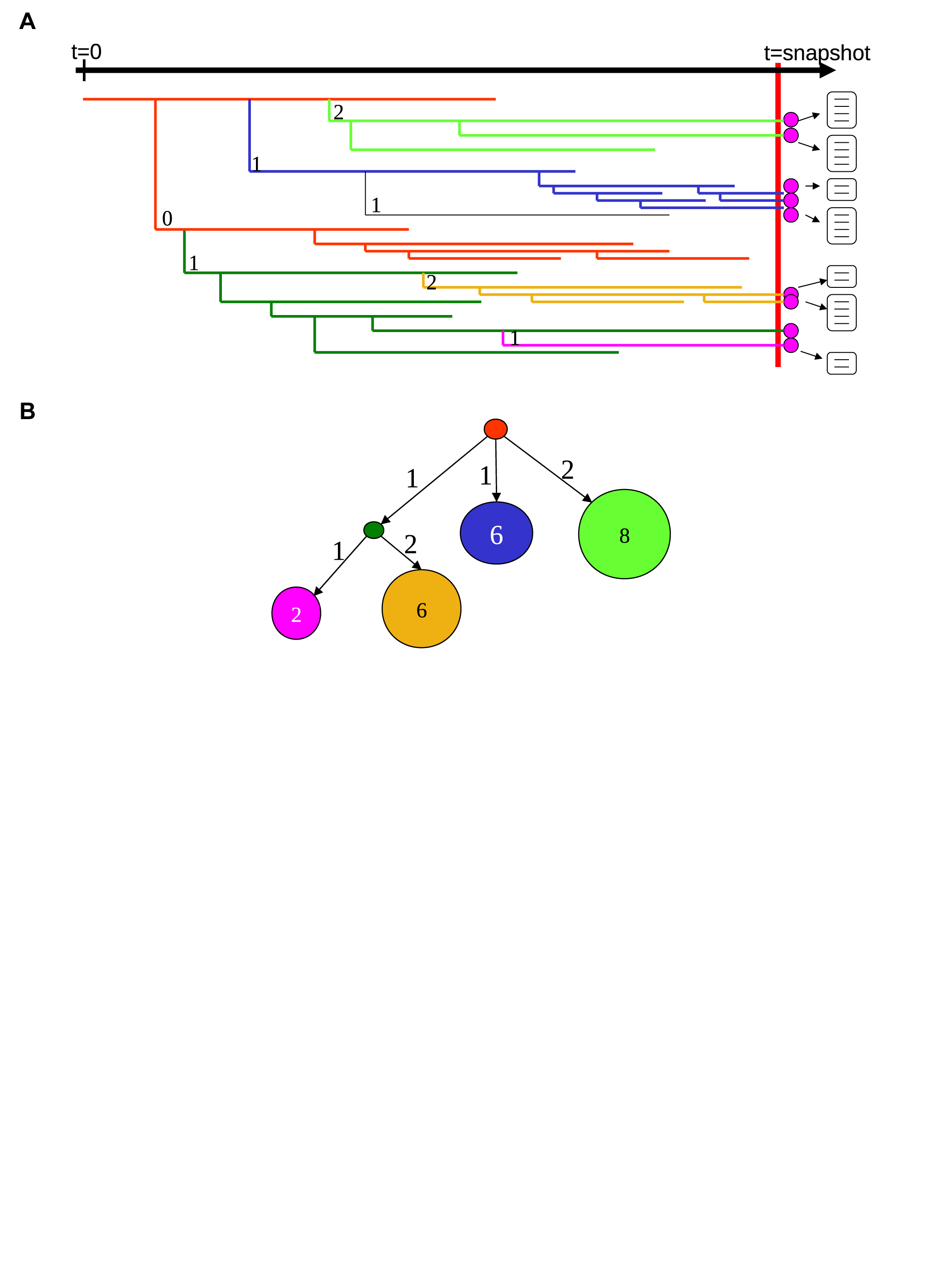

Supplement: Figure S25 — Comparison between binary tree and output from ImmuniTree. (A) A binary tree sampled from the algorithm. Horizontal lines represent lifetimes of cells. Vertical lines represent birth events. Numbers indicate the mutation distance between the child and its parent (0 if not indicated). Cells with identical sequences are marked by the same color. (B) The reported canonical tree after collapsing identical sequences to a single node (of matching color). Number inside the nodes indicate the number of reads associated with that node. (TIF) [file ppat.1003754.s025.tif]

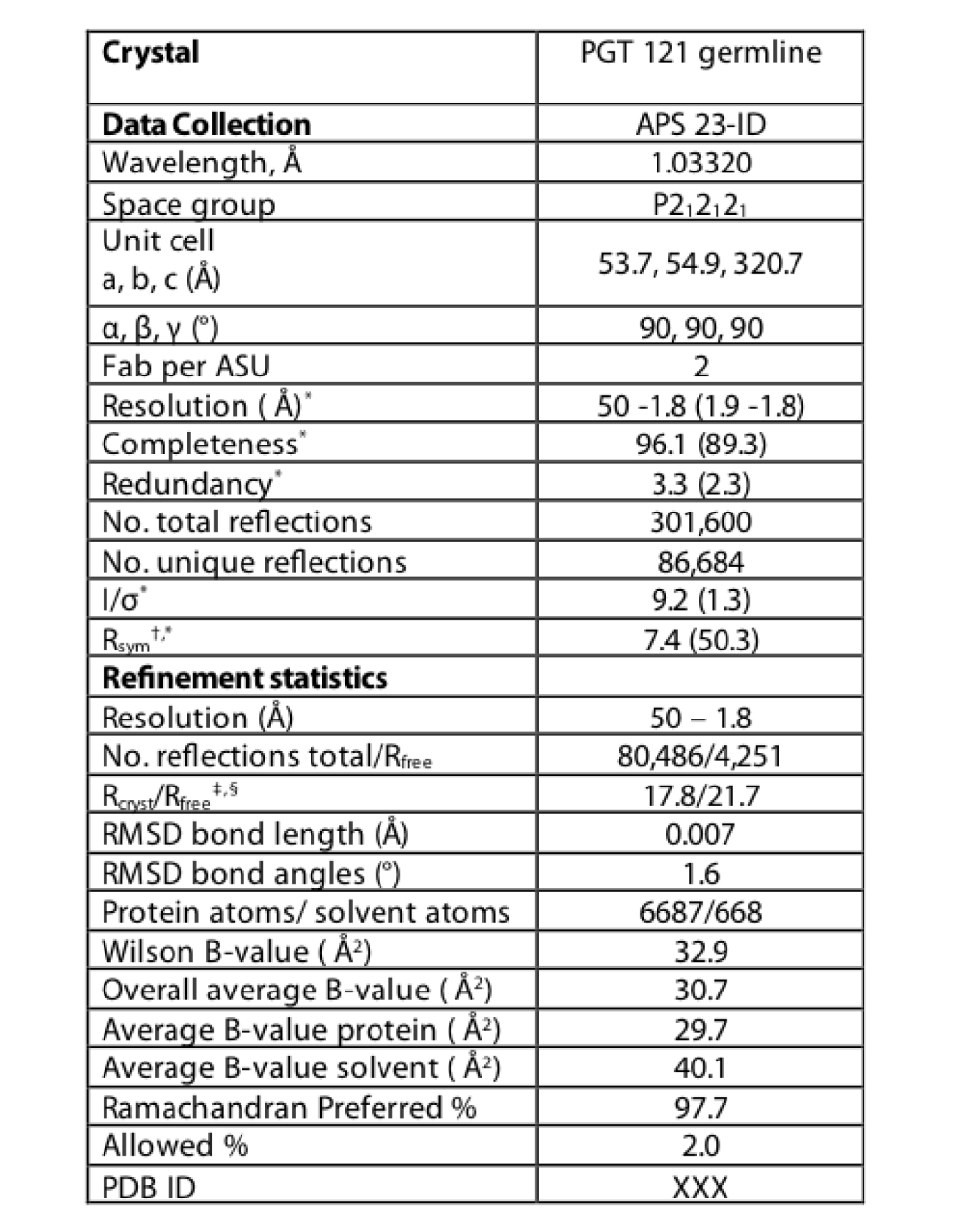

Supplement: Table S1 — X-ray crystallography statistics of PGT121germline. (TIF) [file ppat.1003754.s026.tif]
