# Supplementary material for: The Effects of Somatic Hypermutation on Neutralization and Binding in the PGT121 Family of Broadly Neutralizing HIV Antibodies
Source: PLoS Pathog. 2013 Nov 21;9(11):e1003754. doi: 10.1371/journal.ppat.1003754 (PMC3836729; doi:10.1371/journal.ppat.1003754)
Supplement: Text S1 — Supplementary text describing ImmuniTree. Description of a novel Bayesian method called ImmuniTree, which models antibody somatic hypermutation while accounting for sequencing error. Supplementary text includes in silico experiments and additional details on how the algorithm was designed. (PDF) [file ppat.1003754.s027.pdf]

Supplementary Text

ImmuniTree — A Detailed Description

1 Background

There are a few reasons why the current phylogeny algorithms are ill-equipped for high-throughput HIV sequencing.

- **Sequencing Noise** All currently existing phylogenetic algorithms use a single model of mutation to explain the diversity of the reads, neglecting the variance induced by the noise in the sequencing technology, which is a substantial source of noise precisely in the scenarios that deal with this volume of sequences to analyze. In addition, a different model for sequencing noise would allow to collapse all the sequences who would otherwise be identical to a single representative in the tree, thus simplifying the user output.

- **Emphasis on Long Time-Frames** The standard output of a phylogenetic tree construction algorithm is a binary tree, in which the input sequences are at the leaves. This description exhibits the belief that the mutational process occur continuously over a long time frame, and our observed data only tells us the state at the end of time. However, in a scenario where we get million of reads from a single environmental sample, it is often the case that the whole taxonomy was generated in a short time-frame and the genome population has representatives from all phases of the process. In other words, we might be sequencing grandparents and grandchildren at the same time. In addition, in such an environment, a single sequence might be the parent of more than one immediate child, and the order of the children is irrelevant and practically impossible to infer. Hence, a more useful description of the taxonomy will allow the input sequences to reside at all levels of the tree, and would also lift the ban on non-binary trees. Hence, the long-term continuous mutation model is not only a miss for this type of data, but it also generates outputs that are harder to interpret.

Our algorithm, called ImmuniTree, addresses all of these issues. Our main advantage over the traditional phylogeny methods is that we model the biological phenomena and the experimental setup carefully — and jointly.

First, we model sequencing noise explicitly. This allows us to detect read errors and with that we can assign those reads that differ only due to noise to the same node. Doing so not only simplifies the user output, but it also saves us the needless computational effort of structuring those reads in a tree, thus greatly reducing our search space.

Second, we use a discrete mutation model, in which every edge in the tree represents a single cell copy event. This is a better fit to short time-frame lineages. Third, our method's output tree is not necessarily binary, and the reads are partitioned among all its nodes. This description paints a much better picture on the dominant sequences in the population and how they are related.

Our model is unique in that it represents the cell lineage directly. Each node in the tree represents a particular cell, and the mutation model we use encodes the mutations likely to occur in a single cell division event. This makes our model closer than other models to the actual biological processes that drive the data. However, as with many models, our model is but an approximation to the biological truth, whatever it may be in a particular scenario. In our modeling assumptions we use a birth and death rate that are uniform throughout the tree although in truth some sequences have better fitness and hence proliferate faster. We do not analyze the stability of the sequences in terms of their secondary or ternary structure. We also assume that in each genomic site mutations occur independently.

Example

Consider the clone depicted in Figure S19, generated synthetically using the model we will introduce in Section 3. Each node in the tree is a cell, whose parent cell is indicated by the incoming edge. The number next to the edge is the number of mutations that the child cell has compared to the parent (i.e. the Hamming distance between their sequences). We colored cells that have identical sequences with the same unique color.

We cannot see it in this figure, but not all the cells are alive during sequencing time, some of them might have died before that. We collected 1000 synthetic reads from the "surviving" synthetic cells. The number of reads each

cell produced is indicated by the size of the cell, as well as the number next to it. The synthetic reads are not an exact copy of the cell's sequence; they are produced with an added sequencing noise. For this example we set the noise probability to be roughly 3.5% per base (only base-substitutions, no indels).

If we run one of the existing tree-construction algorithms (here we use FastTree[1]), we will get the result in Figure S20. It is hard to tell from that figure the *story* behind that clone. Even if we were to collapse all the nodes below a certain cutoff distance from the leaves, it is not clear what this cutoff should be, and it is probably not the same cutoff across the entire tree. Moreover, even if we somehow collapsed a whole subtree to a single node, how would we decide what is the sequence of that node?

In contrast, the story of the clone is clearly seen in Figure S21a. The tree there is obtained from the true tree in Figure S19 by collapsing all the edges marked with 0, i.e. collapsing cells with identical sequences to a single node. We call this collapsed tree a *mutation tree*. The size of each node (and the number next to it) is now the total number of reads that were produced by cells with that exact sequence. To see what happens to these reads we now "color" all the reads that belong to a node in by the unique color of the the node as it appears in Figure S21a.

In Figure S21b we see what happens to the "colored" reads after we let ImmuniTree reconstruct a mutation tree for them. The colors inside each node represent the colors of the reads that ImmuniTree decided are associated with that node. You can quickly identify which node in the reconstructed tree corresponds to which node in the true tree based on this composition. From this we can see how closely the reconstructed tree is similar to the correct one. The topology is almost identical (we did not give ImmuniTree information regarding the root, so it got it wrong). While few of the reads are associated with the wrong node (recall, the reads have additional noise that might accidentally make them more similar to a sequence with a different color), most nodes have a strong color consensus corresponding to a single node in the correct mutation tree. We note that the red node with two reads on the left of the true mutation tree, was mistakenly absorbed by its parent, as the algorithm decided those two reads are more likely to be the result of noise.

2 Synthetic Experiments

To test the validity of the tree-construction method, we ran a series of 100 synthetic experiments. In each experiment a synthetic clone, like the one in Figure S19, was randomly drawn, based on the probabilistic model outlined in Section 3. From this synthetic clone, 1000 reads were produced. Each read was copied directly from the sequence of one of the surviving cells in the clone, chosen uniformly, with the addition of sequencing noise.

The probability of a base-change due to sequencing noise was set to 0.006 divided evenly over the 3 possible other bases. The probability of a base transition due to mutation was set to 0.001. For the birth and death rates we set $\lambda = 0.8$ and $\delta = 0.5$.

For each experiment we obtain the true *mutation tree* from the synthetic clone, by collapsing edges with no mutations, as in Figure S21a. In our experiments, the number of nodes in the mutation trees ranged between a single node to 545 nodes, with an average of 84 nodes. We then run ImmuniTree for 2000 iterations on each read set, and the output is a mutation tree, analogous to Figure S21b. We also run FastTree[1] and MrBayes[2] on the same sets, and their output is a binary tree, analogous to Figure S20. MrBayes does not return a single tree but rather many sampled trees, and we chose the tree of the 100,000th sample for these experiments.

We compare the methods using the *missing-branch rate*, defined as the fraction of branches in the true mutation tree that are missing from the reconstructed tree. A branch is viewed as a partition of the reads into two sets (a cut). However, considering the nature of our data, the missing-branch metric might be too harsh, since even a single read at the wrong side of the branch can cause the branch to be "missed". Hence we soften the notion of missing-branch by allowing a limited number of reads to be misplaced before we declare the branch "missing". We define the *missing-branch(k) rate* as the fraction of branches that are missing from the true tree up to k misplaced reads. Hence, missing-branch(0) corresponds to the original missing-branch definition.

The output trees of ImmuniTree and the other methods are different in nature: recall that while our method returns a number of branches comparable to the true tree, FastTree and MrBayes will always return a tree with 1998 branches (for 1000 reads), hence more branches have a chance to "compete" for each true branch. Still, the missing-branch measure is suitable for comparing the trees, because it operates with respect to the same coordinate system in both cases — the branches of the true tree. We computed the average missing-branch(k) rate over the 100 experiments, for different values of k. The results are in Figure S22.

We can see that ImmuniTree is having a better missing-branch(k) rate than the other methods for every value of k. ImmunTree and FastTree start close at $k = 0$ with 23.4% for ImmuniTree and 25.1% for FastTree, but for $k = 2$ ImmuniTree's rate is already at 4.6% (it takes $k = 16$ for FastTree to get that low), and once 6 misplaced reads are allowed, ImmuniTree reaches 1.1%.

To further test the validity of our tree construction algorithm, we used another measure for the compactness of the resulting mutation tree, which we call *tree weight*. The weight of a mutation tree is the total number of mutations (between parent-child pairs) plus the total number of disagreements between reads and the sequences of the nodes they are assigned to. We note that the tree with the minimum weight is not necessarily the most likely tree, since other factors, such as the mutation and noise models, might favor less compact outcomes. Still, it is a sanity check that our trees return reasonable results.

Out of the 100 experiments we conducted, in 22 cases the weight of the reconstructed tree was equal to the true tree. In 42 cases the reconstructed tree was worse than the true tree (by 9.0 units on average). In 36 cases the reconstructed tree was better than the correct tree (by 2.4 units on average).

3 Details of Tree Construction Algorithm

The input for this section is a set of reads that have the same length, fully aligned to each other. Some of the reads may have gaps. The alignment of the reads is given and kept fixed throughout the algorithm. The output is a tree where each node is associated with one sequence, and the reads are associated with the nodes of the tree. The disagreements between the sequence of a node and its parent represent new hypermutations. The disagreements between a read and the sequence of its node represent sequencing errors.

The algorithm relies on a probabilistic model that describes both the expansion process of a clone and the sequencing process. It iteratively explores this probabilistic space, producing a new hypothetical tree in each iteration, aimed at describing how the data was generated. The mechanics of the algorithm guide the search to high-likelihood areas of the space. We return the tree with the highest likelihood.

In our use of Immunitree, we first performed traditional neighbor-joining multiple alignment to align the input reads, and introduced gap characters to represent aligned indel positions. Next, Immunitree was parameterized to treat gap characters like nucleotide characters; prior probabilities were parameterized to recognize that 454-sequencing has high indel rates, whereas SHM has low indel rates. We subsequently sent the gap aligned sequences into Immunitree for MCMC-based inference. After the MCMC chain of phylogenetic trees was generated, we performed a brief optimization on each sample tree. In the per-tree optimization, we collapsed together nodes having identical consensus sequences into the one that was earliest born. Reads and children nodes were all transferred. From these optimized sample trees, we selected the one with the highest log-likelihood.

3.1 Generative Model

The process goes as follows. First, a tree of cells is generated, each cell i has a parent cell $u(i)$, a birth time b_i and a death time d_i. Then, the sequences of these cells are filled in from top to bottom, each sequence $\mathbf{y}_i$ is a copy of its parent, with a slight chance of mutation. Last, reads are generated from the cells alive at the end of the process. Each read j is assigned to a cell s_j and its sequence $\mathbf{x}_j$ is copied from the cell sequence taking into account sequencing noise. We denote by $\mathbf{b}, \mathbf{d}, u(\cdot), \mathbf{s}, Y, X$ the set of all variables of the respective type.

As an example, consider the directed binary tree shown in Figure S23. Edges represent cells; each internal node is a birth event, and has an out-degree of 2 — one outgoing edge represents the parent cell (that continues to live after the birth), and the second outgoing edge is the new cell. Leaves are either death events, or cells alive at time T. The x-axis represents time.

3.1.1 Tree Generation

Cells are being generated using a birth-death process, starting with a single live cell at time $t = 0$. A live cell gives birth to a new cell at a rate λ, and dies after exponential(δ) time from birth. The process stops when $t = T$ ("snapshot time"). We set $T = 10$.

We denote by N the total number of cells generated in the above process, excluding the root. For $i = 0 \dots N$, let b_i and d_i be the respective birth and death time of cell number i (with the root at $i = 0$). Let $u(i)$ be the parent of cell i for $i = 1 \dots N$. We denote by N_{dead} the number of cells that died before $t = T$, i.e. those with $d_i < T$. For the other N_{alive} cells we truncate the d_i value to $d_i = T$.

The likelihood of a given lineage then comes down to:

$$P(u(\cdot), \mathbf{b}, \mathbf{d} | \lambda, \delta) = \lambda^N \cdot \delta^{N_{dead}} \cdot \exp\left\{ -(\lambda + \delta) \sum_{i=0}^{N} (d_i - b_i) \right\} \tag{1}$$

We put a Gamma($k_\lambda, 1$) prior on the birth rate λ and a Gamma($k_\delta, 1$) prior on the death rate δ. In our algorithm we set $k_\lambda = 1$ and $k_\delta = 0.75$.

3.1.2 Sequence Generation

We associate a sequence y_i for each cell in the tree. We assume that the length L of the nucleotide sequences is given, as well as the sequence of the root cell $\mathbf{y}_0$. For cells $1 \dots N$, ordered topologically, seqeunce $\mathbf{y}_i$ is drawn based on a mutation model $\mathcal{M}$ parameterized by the parent sequence $y_{u(i)}$, and global parameters $\theta_{\mathcal{M}}$ (omitted sometimes for reasons of clarity):

$$\mathbf{y}_i \sim \mathcal{M}(\mathbf{y}_{u(i)}, \theta_{\mathcal{M}}) \quad i = 1 \dots N$$

3.1.3 Read Generation

We assume that the number of reads M is given. We associate each read j to a live cell s_j by choosing uniformly from the N_{alive} alive cells. Copy the read's letters from the sequence of the cell they were assigned to, using a sequencing noise model $\mathcal{R}$ parameterized by that sequence $\mathbf{y}_{s_j}$ and $\theta_{\mathcal{R}}$ (sometimes omitted):

$$\mathbf{x}_j \sim \mathcal{R}(\mathbf{y}_{s_j}, \theta_{\mathcal{R}}) \quad j = 1 \dots M$$

Note that the number of live cells now plays a role in the joint likelihood:

$$P(\mathbf{s}|\mathbf{d}) = \frac{1}{(N_{alive})^M} \tag{2}$$

3.1.4 Joint Likelihood

Given the tree structure, the root sequence, and the read assignments, the probability over the cell sequences and reads is given by

$$P(Y, X | u(\cdot), \mathbf{s}, \mathbf{y}_0, \theta_{\mathcal{M}}, \theta_{\mathcal{R}}) = \prod_{i=1}^{N} \mathcal{M}(\mathbf{y}_i; \mathbf{y}_{u(i)}) \prod_{j=1}^{M} \mathcal{R}(\mathbf{x}_j; \mathbf{y}_{s_j}) \tag{3}$$

when we adopt standard notation $\mathcal{M}(y; y')$ for the probability of y if drawn from $\mathcal{M}(y')$, and similarly for $\mathcal{R}$. We describe in the next sections the details of the models $\mathcal{M}$ and $\mathcal{R}$, as well as the prior for their parameters $\theta_{\mathcal{M}}$ and $\theta_{\mathcal{R}}$.

The variables $\xi \triangleq (X, Y, \mathbf{b}, \mathbf{d}, u(\cdot), \mathbf{s}, \theta_{\mathcal{M}}, \theta_{\mathcal{R}}, \lambda, \delta)$ describe the full state of the model. The overall probability for a particular state is simply the product of equations (1), (2), and (3), and the priors over $\lambda, \delta, \theta_{\mathcal{M}}, \theta_{\mathcal{R}}$ in case they are not fixed by the user.

3.1.5 A Prior Over Transition Matrices

When we discuss of mutational processes, one often uses the notion of a *transition matrix.* Such a matrix M tells the probability of a base transition from each parent base j to each child base i. That is, M_{ij} is the probability of letter j changing to i. Hence, a transition matrix is always square in the size of the alphabet, and each column's values represent a distribution and hence sum to 1. Since we will use transition matrices heavily in the coming chapters, we decided to introduce some notation for it here.

Let $\hat{M}$ be a d-dimensional square matrix with positive entries. We say that $M \sim \text{Matrix}(\hat{M})$ if every column of M is drawn independently from a Dirichlet distribution whose parameters are the respective column in $\hat{M}$. To put it in MATLAB notation:

$$M_{:,j} \sim \text{Dirichlet}(\hat{M}_{:,j}) \quad j = 1 \dots d$$

The log likelihood of M is then given by:

$$p(M|\hat{M}) \propto \prod_{i,j} (M_{ij})^{\hat{M}_{ij}-1} \tag{4}$$

Let $\mathcal{I}_d$ be the identity matrix of dimension d. We define

$$\text{Transition}(d, \epsilon, \xi) = \xi(\epsilon + (1 - d\epsilon)\mathcal{I}_d)$$

to be the square matrix of dimension d where each column sums to ξ and each off-diagonal entry is equal to $\xi\epsilon$. For example, $\text{Transition}(4, 0.01, 100)$ is

$$\begin{bmatrix} 97 & 1 & 1 & 1 \\ 1 & 97 & 1 & 1 \\ 1 & 1 & 97 & 1 \\ 1 & 1 & 1 & 97 \end{bmatrix}.$$

We can now draw a d-dimensional transition matrix $M \sim \text{Matrix}(\text{Transition}(d, \epsilon, \xi))$ that on average exhibits an ϵ chance of mutation, and the deviation from this average is reduced by increasing ξ.

3.1.6 Description of $\mathcal{M}$

Our mutation model for $\mathcal{M}$ is nucleotide based, where a 4x4 transition matrix $M^{(k)}$ controls the mutation probabilities between any two bases for each of the sequence positions $k = 1..L$. The indexes 1..4 correspond to the letters ACGT.

Thus, the likelihood over the cell sequences $\mathbf{y}$ can be given in terms of $n_{ij}^{(k)}$ counting the number of mutations in the entire tree from letter j to letter i in location k:

$$P\left(Y|u(\cdot),\mathbf{M}\right) = \prod_{k=1}^{L}\prod_{i=1}^{4}\prod_{j=1}^{4}\left(M_{ij}^{(k)}\right)^{n_{ij}^{(k)}} \tag{5}$$

If M is not given by the user, we put a prior over it:

$$M \sim \text{Matrix}\left(\text{Transition}(4, \epsilon_M, \xi_M)\right) \tag{6}$$

In our implementation, we used $\epsilon_M = 0.04$, $\xi_M = 630$.

3.1.7 Description of $\mathcal{R}$

We use a simple nucleotide 4x4 transition matrix $\theta_{\mathcal{R}} = R$, to copy the reads from their respective cells, hence if $\mathbf{x} \sim \mathcal{R}(\mathbf{y};\theta_{\mathcal{R}})$ it means that

$$x_l \sim \text{Multinomial}(R_{:,y_l}) \quad l = 1 \dots L$$

where L is the length of the reads (in nucleotides). The likelihood over the read sequences X can be thus given in terms of m_{ij} counting the number of transitions from letter j to letter i across all the reads:

$$P(X|Y,\mathbf{s},R) = \prod_{i=1}^{4}\prod_{j=1}^{4}(R_{ij})^{m_{ij}} \tag{7}$$

If R is not given by the user, we put a prior over it:

$$R \sim \text{Matrix}\left(\text{Transition}(4, \epsilon_R, \xi_R)\right) \tag{8}$$

In our implementation, we used $\epsilon_R = 0.008$, $\xi_R = 6.3 \cdot 10^6$.

3.2 Inference

Inference is done using the Markov Chain Monte Carlo (MCMC)*(37)* algorithm. The algorithm generates particles, each corresponds to a full assignment to all the hidden variables of the model. Each particle contains a representation of the induced tree, the sequences associated with the nodes, and the assignment of the N reads to these nodes. We can compute the probability to each such particle using our probabilistic model.

Let ξ be our current particle, and ξ' the proposed particle. Let $\mathcal{T}(\xi \to \xi')$ be the probability for proposing ξ' given that the current particle is ξ. According to the Metropolis-Hastings framework, we should accept the transition to ξ' with probability

$$\min\left[1, \frac{P(\xi')\mathcal{T}(\xi' \to \xi)}{P(\xi)\mathcal{T}(\xi \to \xi')}\right] \tag{9}$$

Usually, the transition distribution $\mathcal{T}$ does not support all particles, but only a small subset $\mathcal{I}_\xi$. As long as for any two particles there exist some combination of moves that can take you with a positive probability from one to the other, the distribution over the particles converges to the correct posterior distribution over the hidden variables.

The computation of the acceptance probability becomes even easier if we set

$$\mathcal{T}(\xi \to \xi') = \frac{P(\xi')}{Z_\xi}$$

for each supported particle $\xi' \in \mathcal{I}_\xi$. That is, $\mathcal{T}$ gives each particle a weight proportional to its probability and $Z_\xi = \sum_{\xi' \in \mathcal{I}_\xi} P(\xi)$ is the normalization constant. Since the transition makes only a local change, the particles in $\mathcal{I}_\xi$ will have many common factors that will eventually cancel out in $\mathcal{T}$ and hence need not be computed. The acceptance probability then becomes

$$\min\left[1, \frac{Z_\xi}{Z_{\xi'}}\right]. \tag{10}$$

If the set of particles supported by $\mathcal{T}$ does not change between ξ and ξ', i.e. $\mathcal{I}_\xi = \mathcal{I}_{\xi'}$, then $Z_\xi = Z_{\xi'}$ and the acceptance probability becomes 1. This is how Gibbs sampling is derived.

Notation: when the current particle is clear from context we might just write $\mathcal{T}(\xi')$ instead of $\mathcal{T}(\xi \to \xi')$. Also, if the transition changes only a fixed subset of the particle variables, we might just write those variables instead of ξ'.

3.2.1 Sequence Update

Here the tree and the assignments of reads to the nodes of the tree are fixed, and we sample the sequences of the nodes. In other words, we are interested to sample from $P(Y \mid X, u(\cdot), \mathbf{s})$. Consider the simple case where each sequence is just one letter. Examining equation (3), we notice that the problem can be formulated as a Markov Random Field (MRF) over a factor tree, which allows for an efficient joint sampling of all the sequences Y. Our MRF then has these factors:

Singletons, for each node $i = 0 \dots N$:	$\psi(\mathbf{y}_i) \triangleq \prod_{j:s_j=i} \mathcal{R}(\mathbf{x}_j; \mathbf{y}_i)$
Pairwise, for each child-parent pair:	$\phi(\mathbf{y}_i, \mathbf{y}_{u(i)}) = \mathcal{M}(\mathbf{y}_i; \mathbf{y}_{u(i)})$

A standard message-passing algorithm is used to pass messages from the bottom towards the root. Each node $i = 1 \dots N$ sends a message $m_{i \to u(i)}(\mathbf{y}_{u(i)})$ to its parent, defined recursively as follows. Let i be a node in the tree with a parent p and K children $v_1 \dots v_K$. Let $m_{v_k \to i}(\mathbf{y}_i)$ be the messages sent to i from its children. Then:

$$\tau_{i \to p}(\mathbf{y}_i, \mathbf{y}_p) \triangleq \phi(\mathbf{y}_i, \mathbf{y}_p)\psi(\mathbf{y}_i) \prod_{k=1}^{K} m_{v_k \to i}(\mathbf{y}_i) \tag{11}$$

$$m_{i \to p}(\mathbf{y}_p) \triangleq \sum_{\mathbf{y}_i} \tau_{i \to p}(\mathbf{y}_i, \mathbf{y}_p) \tag{12}$$

It can be shown using induction that

$$\tau_{i \to p}(\mathbf{y}_i, \mathbf{y}_p) = P(\mathbf{y}_i, \{x_j : s_j \text{ is descendant of } i\} \mid \mathbf{y}_p, u(\cdot), \mathbf{s})$$

From which if follows that:

$$P(\mathbf{y}_i \mid \mathbf{y}_p, X, u(\cdot), \mathbf{s}) \propto \tau_{i \to p}(\mathbf{y}_i, \mathbf{y}_p) \tag{13}$$

We use (13) to sample the sequences from top to bottom (recall that the root sequence $\mathbf{y}_0$ is given). For the case where the sequences are longer than one letter, we notice that all the factors are independent over the letter positions, and hence we simply apply the above algorithm to each position independently.

3.2.2 Local Tree Manipulation

Let v be an internal node in the binary tree. Consider the following environment of a node v in the tree (See Figure S24).

Each horizontal line is a cell. We will call the top cell R. v is a birth event in R's life, and nodes u and w are the previous and next events (we assume here they are both birth events). The nodes f, e, b, c represent the next event in each cell's life, either birth or death. Similarly, the node a represents the event preceding u on R's life, which could be either another birth or R's own birth. Our move (inspired by Holder[3] proposes to disconnect the edge $v \to c$ from the tree, and reconnect it to a new position on this subgraph, chosen uniformly based on the edges length. The only constraint is that event c's time is fixed, hence the new location of node v must occur before c in time. If u or w are leaves then we reduce the reconnection domain to include only the part of the subgraph that actually exists.

Note that this move is reversible, which permits the use of the Metropolis-Hastings framework. To calculate the likelihood difference between the current particle and the proposed one, all we need is the new length of the edge $v \to c$, and (if v changed parent) the likelihood of the sequences of the cell born at v given its parent sequence.

3.2.3 Global Tree Manipulation

Let i be a cell. This move changes $u(i)$, the parent cell of i, keeping everything else fixed. Essentially, it is a Gibbs move over $u(i)$. Of course, we only need to consider as parents the cells alive at time b_i. The probability assigned to each potential parent i', $p(u(i) = i' \mid \text{everything else})$, is proportional to $\mathcal{M}(\mathbf{y}_i; \mathbf{y}_{i'})$. Here the acceptance probability is 1.

3.2.4 Change Death Time

For each cell i with no reads, we sample a new death time d_i. Let $\text{children}(i) = \{i' : u(i') = i\}$ and define $f = \max(\{b_{i'} \mid i' \in \text{children}(i)\} \cup \{b_i\})$ to be the time of the last recorded event in i's life. We sample $r \sim \text{exponential}(\lambda + \delta)$, and propose $d'_i = \min(f + r, T)$. We observe the acceptance probability of this proposal in the following four cases:

Case 1: $d_i, d'_i < T$. In that case the likelihood difference is given in terms of equation (1).

$$\frac{p(\xi')}{p(\xi)} = \exp\{-(\lambda+\delta)(d'_i - d_i)\} = \frac{(\lambda+\delta)\exp\{-(\lambda+\delta)(d'_i - f)\}}{(\lambda+\delta)\exp\{-(\lambda+\delta)(d_i - f)\}}$$
$$= \frac{p(r = d'_i - f)}{p(r = d_i - f)} = \frac{\mathcal{T}(d'_i)}{\mathcal{T}(d_i)}$$

and hence we accept with probability 1.
Case 2: $d_i = d'_i = T$. In that case nothing changed.
Case 3: $d_i < d'_i = T$. The number of live cells grew from N_{alive} to $N_{alive}+1$. The likelihood difference is now based on equations (2) and (1):

$$\frac{p(\xi')}{p(\xi)} = \frac{1}{\delta}\exp\{(\lambda+\delta)(T-d_i)\} \cdot \left(\frac{N_{alive}}{N_{alive}+1}\right)^N$$

$$\frac{\mathcal{T}(d'_i)}{\mathcal{T}(d_i)} = \frac{p(r > T - f)}{p(r = d_i - f)} = \frac{\exp\{-(\lambda+\delta)(T-f)\}}{(\lambda+\delta)\exp\{-(\lambda+\delta)(d_i - f)\}}$$
$$= \frac{\exp\{-(\lambda+\delta)(T-d_i)\}}{\lambda+\delta}$$

And the acceptance probability is:

$$\min\left[1, \frac{P(\xi')\mathcal{T}(\xi' \to \xi)}{P(\xi)\mathcal{T}(\xi \to \xi')}\right] = \min\left[1, \frac{\lambda+\delta}{\delta}\left(\frac{N_{alive}}{N_{alive}+1}\right)^M\right]$$

where N_{alive} is the number of live cells in the current particle (ξ) and M is the number of reads.
Case 4: $d'_i < d_i = T$ is the opposite of Case 3, and using the same derivation the acceptance probability is

$$\min\left[1, \frac{\delta}{\lambda+\delta}\left(\frac{N_{alive}}{N_{alive}-1}\right)^M\right]$$

Notice that with many reads, it will be harder to accept a proposal to create a new live cell. This is because the reads are assigned to the live cells via a uniform distribution. Adding a new live cell increases this uniform space of assignment by an order of magnitude, and hence reduces the likelihood of the current assignment, as can be seen from (2).

3.2.5 Spawn/Absorb A Leaf Node

Let i be a cell. This move either creates a new child of i or erases one of its current children. There are two cases, depending on whether i is a live cell or not.

Case 1: The cell i is dead ($d_i < T$). Let $\mathcal{C}$ be the children of i that have no children of their own and no reads assigned to them. Let $|\mathcal{C}|$ be the size of $\mathcal{C}$. If $\mathcal{C}$ is empty, propose to create a new cell i' whose parent is i. Otherwise, with probability $1-\rho$ create a new cell as above, and with probability ρ delete one of the cells in $\mathcal{C}$, chosen uniformly. In the case we proposed to create a new child i', we also sample its sequence content $\mathbf{y}_{i'}$, its birth time $b_{i'}$ and death time $d_{i'}$ from the prior, except we condition on $b_{i'} < T$:

$$\begin{aligned}
\mathbf{y}_{i'} &\sim \mathcal{M}(\mathbf{y}_i)\\
r_1 &\sim \text{Exponential}(\lambda)\\
b_{i'} &= b_i + (r_1 \mod (T - b_i))\\
r_2 &\sim \text{Exponential}(\delta)\\
d_{i'} &= \min(b_{i'} + r_2, T)
\end{aligned}$$

Notice that $b_{i'} - b_i$ is distributed Exponential(λ) with the added condition that $b_{i'} - b_i < T - b_i$, or in other words $p(b_{i'} - b_i = x) = \frac{p(r_1 = x)}{p(r_1 < T - b_i))}$.

What is the acceptance probability for the above proposal? Let ξ' be the new particle with the new cell i', and ξ the original particle. Let's assume for now (it will not matter later) that the new cell came out dead ($d_{i'} < T$) and that $|\mathcal{C}| > 0$. In this case:

$$\begin{aligned}\mathcal{T}(\xi \to \xi') &= (1-\rho)\cdot \mathcal{M}(\mathbf{y}_{i'};\mathbf{y}_i)\cdot p(b_{i'})\cdot p(d_{i'} \mid b_{i'})\\ &= (1-\rho)\cdot \mathcal{M}(\mathbf{y}_{i'};\mathbf{y}_i)\cdot \frac{p(r_1 = b_{i'} - b_i)}{p(r_1 < T - b_i)}\cdot p(r_2 = d_{i'} - b_{i'})\\ &= (1-\rho)\cdot \mathcal{M}(\mathbf{y}_{i'};\mathbf{y}_i)\cdot \frac{\lambda \exp\{-\lambda(b_{i'} - b_i)\}}{1-\exp\{-\lambda(T-b_i)\}}\cdot \delta \exp\{-\delta(d_{i'} - b_{i'})\}\\ \mathcal{T}(\xi' \to \xi) &= \frac{\rho}{|\mathcal{C}|+1}\end{aligned}$$

$$\frac{p(\xi')}{p(\xi)} = \mathcal{M}(\mathbf{y}_{i'};\mathbf{y}_i)\cdot \lambda \cdot \delta \cdot \exp\{-(\lambda+\delta)(d_{i'} - b_{i'})\}$$

Combining the above equations we get that:

$$\frac{P(\xi')\mathcal{T}(\xi' \to \xi)}{P(\xi)\mathcal{T}(\xi \to \xi')} = \frac{\rho \exp\{-\lambda(b_i + d_{i'} - 2b_{i'})\}\,(1-\exp\{-\lambda(T-b_i)\})}{(1-\rho)(|\mathcal{C}|+1)} \tag{14}$$

whose minimum with 1 forms the acceptance probability for this case. The acceptence probability for the reverse move (deleting a cell) is given by replacing the numerator and the denominator in the above equation.

Case 2: The cell i is alive ($d_i = T$). Let $\mathcal{C}$ be the children of i that have no children of their own (contrary to the previous case, we allow them to have reads). We use exactly the same procedure as for case 1 to decide whether we create a new cell or delete one of the cells in $\mathcal{C}$. If we delete a child of i, we assign all its associated reads to i. If we create a new cell i', then we determine $b_{i'}, d_{i'}$ and $\mathbf{y}_{i'}$ like before, and for each read j currently assigned to i, we set

$$s_j = \begin{cases} i' & \text{with probability } \frac{\mathcal{R}(\mathbf{x}_j;\mathbf{y}_{i'})}{\mathcal{R}(\mathbf{x}_j;\mathbf{y}_i)+\mathcal{R}(\mathbf{x}_j;\mathbf{y}_{i'})}\\ i & \text{otherwise}\end{cases}$$

How does this effect the acceptance probability? The quantity $\mathcal{T}(\xi \to \xi')$ of case 1 now needs to be multiplied by the product of the above probabilities (for each read use the probability that produced its outcome). The quantity $\frac{p(\xi')}{p(\xi)}$ now needs to be multiplied by $\frac{\mathcal{R}(\mathbf{x}_j;\mathbf{y}_{i'})}{\mathcal{R}(\mathbf{x}_j;\mathbf{y}_i)}$ for every read j that moved to i'.

3.2.6 Assign Reads To Cells

For every read j we need to set s_j to the identity of one of the live cells. Naively one could use Gibbs updates on each s_j by considering all the live cells and assigning read j to cell i with probability

$$p(s_j = i|\text{all other variables}) \propto \mathcal{R}(\mathbf{x}_j;\mathbf{y}_i).$$

This computation is expensive ($O(MN_{alive})$). Instead we use slice sampling[4], which is a special class of Metropolis-Hastings moves.

In Slice sampling we first choose a likelihood threshold r (less than the likelihood of the current particle), and then consider only particles with likelohood greater than r. The next particle ξ' can be obtained from the current particle ξ by choosing uniformly from a range of particles around ξ that shrinks with every rejection until we finally draw a particle above the threshold. This combined approach of rejection sampling and shrinking sampling range really speeds up the more traditional Gibbs sampling, because we only compute the likelihood of a small subset of candidate values.

The procedure goes as follows. First, we reindex the live cells using a pre-order traversal on the tree (i.e. cells are numbered in the order they are visited by a depth-first traversal). This would roughly give close indexes to cells that are close to each other on the tree. The reason we want similar nodes to be indexed similarly is in order to increase the mixing. This way, even as we narrow our range around the current particle, there is still a high likelihood of accepting a new, different, particle. Then, for every read j:

1. Choose a threshold $r \sim$ Uniform $\left([0, \mathcal{R}(\mathbf{x}_j;\mathbf{y}_{s_j})]\right)$
2. Set $\alpha = 1; \beta = N_{alive}$
3. Sample $s'_j \sim \text{Uniform}(\alpha, \dots, \beta)$
4. *while* $\mathcal{R}(\mathbf{x}_j;\mathbf{y}_{y_{s'_j}}) < r$
   (a) *if* $s'_j < i$ set $\alpha = s'_j + 1$ *else* set $\beta = s'_j - 1$

(b) Sample $s'_j \sim \text{Uniform}(\alpha, \ldots, \beta)$

5. set $s_j = s'_j$

This essentially works in $O(M)$ since the *while* loop typically has very few iterations. Each such iteration narrows the interval of cells allowed for assignment and also focuses on cells that lie closer on the tree to the current cell, and hence have a higher likelihood to pass the threshold r. This also results in better mixing of the Markov Chain.

3.2.7 Update Birth/Death Rates

Recall that $\lambda \sim \text{Gamma}(k_\lambda, 1)$ and a $\delta \sim \text{Gamma}(k_\delta, 1)$. We use Gibbs sampling to sample both λ and δ from their posteriors (given all other variables), which turn out to be Gamma distributions as well. Recalling that

$$\text{Gamma}(x; k, \theta) \propto x^{k-1} \exp^{-\frac{x}{\theta}},$$

and using equation (1), the posterior over λ is:

$$\begin{aligned} P(\lambda \mid \text{all other variables}) &\propto P(u(\cdot), \mathbf{b}, \mathbf{d} \mid \lambda, \delta) \cdot \text{Gamma}(\lambda; k_\lambda, 1) \\ &\propto \lambda^{N+k_\lambda-1} \cdot \exp\left\{-\lambda\left(1 + \sum_{i=0}^{N}(d_i - b_i)\right)\right\} \\ &\propto \text{Gamma}\left(\lambda; N + k_\lambda, \left(1 + \sum_{i=0}^{N}(d_i - b_i)\right)^{-1}\right) \end{aligned}$$

Similarly, the posterior over δ is $\text{Gamma}\left(N_{dead} + k_\delta, \left(1 + \sum_{i=0}^{N_{dead}}(d_i - b_i)\right)^{-1}\right)$.

3.2.8 Updating Mutation And Noise Model Parameters

The parameters $\theta_{\mathcal{M}}$ for the mutation model $\mathcal{M}$ are simply the values of the matrix M, which is 4x4. If M is not given by the user, then each column of M is sampled from a Dirichlet distribution, with the parameters of each distribution stored in the corresponding column of the matrix $\hat{M} = \text{Transition}(4, \epsilon_{\mathcal{M}}, \xi_{\mathcal{M}})$.

Let n_{ij} count the number of base mutations from base j to base i among the cells in the tree of the current particle, and construct from those values the matrix $\hat{n} = [n_{ij}]_{i,j=1\ldots4}$. The conjugacy between the Dirichlet distribution and the multinomial makes the posterior over M given all the other variables yet another Dirichlet that we can easily sample M from:

$$M|Y, u(\cdot) \sim \text{Matrix}(\hat{n} + \hat{M})$$

Similarly, the parameters $\theta_{\mathcal{R}}$ for the read noise model $\mathcal{R}$ are simply the values of the matrix R, which is 4x4. Here again, there is a simple form for the posterior over R:

$$R|X, Y, bs \sim \text{Matrix}(\hat{m} + \hat{R})$$

where $\hat{R} = \text{Transition}(4, \epsilon_{\mathcal{R}}, \xi_{\mathcal{R}})$, and $\hat{m} = [m_{ij}]_{i,j=1\ldots4}$ is a matrix that consists of the counts m_{ij}, the number of tranisitions from letter j (in the cell) to the letter i (in the read).

3.3 Output

At every iteration, the algorithm described above produces a tree with the node sequences fully specified and reads associated with them. For each such tree we perform a local optimization to a canonical tree as follows: (1) erase all the subtrees that have no reads at all; (2) collapse all edges where the sequences of the parent and child are identical; (3) remove nodes with out-degree 1 by joining their child and parent directly. We compute the likelihood of each of these canonical trees, and report the tree with the highest likelihood.

References

1. M. N. Price, P. S. Dehal, A. P. Arkin. FastTree 2–approximately maximum-likelihood trees for large alignments. *PloS one*, 5(3):9490, January 2010.

2. F. Ronquist, J. P. Huelsenbeck. MrBayes 3: Bayesian phylogenetic inference under mixed models. *Bioinformatics*, 19(12):71572–1574, August 2003.

3. M. Holder, P. Lewis, D. Swofford, and B. Larget. Hastings Ratio of the LOCAL Proposal Used in Bayesian Phylogenetics. *Systematic Biology*, 54(6):961965, December 2005.

4. R. M. Neal. Slice Sampling. *The Annals of Statistics*, 31(3):pp. 705741, June 2003.
