# Supplementary material for: Correction: The Effects of Somatic Hypermutation on Neutralization and Binding in the PGT121 Family of Broadly Neutralizing HIV Antibodies
Source: PLoS Pathog. 2013 Dec 30;9(12):10.1371/annotation/f1f8c791-61e9-45c6-a455-92c6dadf9f06. doi: 10.1371/annotation/f1f8c791-61e9-45c6-a455-92c6dadf9f06 (PMC3875691; doi:10.1371/annotation/f1f8c791-61e9-45c6-a455-92c6dadf9f06)
Supplement: Supplementary file 1 [file ppat.f1f8c791-61e9-45c6-a455-92c6dadf9f06.s001.docx]

| **Crystal** | PGT 121 germline |
| --- | --- |
| **Data Collection** | APS 23-ID |
| Wavelength, Å | 1.03320 |
| Space group | P2_1_2_1_2_1_ |
| Unit cell  a, b, c (Å) | 53.7, 54.9, 320.7 |
| α, β, γ (°) | 90, 90, 90 |
| Fab per ASU | 2 |
| Resolution ( Å) ^*^ | 50 -1.8 (1.9 -1.8) |
| Completeness^*^ | 96.1 (89.3) |
| Redundancy^*^ | 3.3 (2.3) |
| No. total reflections | 301,600 |
| No. unique reflections | 86,684 |
| I/σ^*^ | 9.2 (1.3) |
| R_sym_^†,*^ | 7.4 (50.3) |
| **Refinement statistics** |  |
| Resolution (Å) | 50 – 1.8 |
| No. reflections total/R_free_ | 80,486/4,251 |
| R_cryst_/R_free_^‡, §^ | 17.8/21.7 |
| RMSD bond length (Å) | 0.007 |
| RMSD bond angles (°) | 1.6 |
| Protein atoms/ solvent atoms | 6687/668 |
| Wilson B-value ( Å^2^) | 32.9 |
| Overall average B-value ( Å^2^) | 30.7 |
| Average B-value protein ( Å^2^) | 29.7 |
| Average B-value solvent ( Å^2^) | 40.1 |
| Ramachandran Preferred % | 97.7 |
| Allowed % | 2.0 |
| PDB ID | 4NPY |
